# Discovery and Anticancer Screening of Novel Oxindole-Based Derivative Bearing Pyridyl Group as Potent and Selective Dual FLT3/CDK2 Kinase Inhibitor

**DOI:** 10.3390/ph17050659

**Published:** 2024-05-20

**Authors:** Aya Soudi, Onur Bender, Ismail Celik, Amer Ali Abd El-Hafeez, Rumeysa Dogan, Arzu Atalay, Eslam B. Elkaeed, Aisha A. Alsfouk, Elshimaa M. N. Abdelhafez, Omar M. Aly, Wolfgang Sippl, Taha F. S. Ali

**Affiliations:** 1 Department of Medicinal Chemistry, Faculty of Pharmacy, Minia University, Minia 61519, Egypt; 2 Biotechnology Institute, Ankara University, Ankara 06135, Turkey; 3 Department of Pharmaceutical Chemistry, Faculty of Pharmacy, Erciyes University, Kayseri 38280, Turkey; 4 Department of Medicinal Chemistry, Institute of Pharmacy, Martin-Luther-University of Halle-Wittenberg, 06120 Halle, Germany; 5 Pharmacology and Experimental Oncology Unit, Department of Cancer Biology, National Cancer Institute, Cairo University, Cairo 11796, Egypt; 6 Department of Pharmaceutical Sciences, College of Pharmacy, AlMaarefa University, Riyadh 13713, Saudi Arabia; 7 Department of Pharmaceutical Sciences, College of Pharmacy, Princess Nourah bint Abdulrahman University, Riyadh 11671, Saudi Arabia; 8 Department of Medicinal Chemistry, Faculty of Pharmacy, Port Said University, Port Said 42511, Egypt

**Keywords:** oxindole, FLT3, CDK2, kinase inhibitor, dual inhibitor, colon cancer, leukemia

## Abstract

Protein kinases regulate cellular activities and make up over 60% of oncoproteins and proto-oncoproteins. Among these kinases, FLT3 is a member of class III receptor tyrosine kinase family which is abundantly expressed in individuals with acute leukemia. Our previous oxindole-based hit has a particular affinity toward FLT3 (IC_50_ = 2.49 μM) and has demonstrated selectivity towards FLT3 ITD-mutated MV4-11 AML cells, with an IC_50_ of 4.3 μM. By utilizing the scaffold of the previous hit, sixteen new compounds were synthesized and screened against NCI-60 human cancer cell lines. This leads to the discovery of a potent antiproliferative compound, namely **5l**, with an average GI_50_ value against leukemia and colon cancer subpanels equalling 3.39 and 5.97 µM, respectively. Screening against a specific set of 10 kinases that are associated with carcinogenesis indicates that compound **5l** has a potent FLT3 inhibition (IC_50_ = 36.21 ± 1.07 nM). Remarkably, compound **5l** was three times more effective as a CDK2 inhibitor (IC_50_ = 8.17 ± 0.32 nM) compared to sunitinib (IC_50_ = 27.90 ± 1.80 nM). Compound **5l** was further analyzed by means of docking and molecular dynamics simulation for CDK2 and FLT3 active sites which provided a rational for the observed strong inhibition of kinases. These results suggest a novel structural scaffold candidate that simultaneously inhibits CDK2 and FLT3 and gives encouragement for further development as a potential therapeutic for leukemia and colon cancer.

## 1. Introduction

Cancer is an extremely diverse disease, encompassing over 200 distinct types, and its origins are quite intricate. Globally, cancer is widely regarded as the most prevalent and concerning disease that impacts human health. It is referred to as a collection of diseases that are characterized by the uncontrolled proliferation and division of cells. Cancer has hallmarks such as exploiting growth signals, evading suppressive mechanisms, resisting cell death, inducing migration and angiogenesis. In addition, emerging phenotypes of cancer continue to be defined especially related to cell metabolism [1,2,3]. Cancer diagnosis and treatment protocols are based on the elements of these features. In such a complex disease, efforts are focused on the development of functional agents such as small molecule combinations, immunochemotherapeutics, dual inhibitors, multi-target peptides and smart drugs [4,5,6]. Thus, pharmacologically active sources, novel designs, repurposing, biological inhibitors, various manipulators and artificial intelligence are used for multifunctional therapeutic formulations and rationalizations [7,8,9,10,11,12].

Protein kinase enzymes play a crucial part in the signal transduction pathways that regulate numerous cellular functions. The majority of cancer types are caused by protein kinases malfunction, with protein kinases being responsible for more than 60% of all oncoproteins and proto-oncoproteins (the main contributors to the cancer condition) [13]. One of these protein kinases is the Fms-like receptor tyrosine kinase 3 (FLT3), which belongs to the class III receptor tyrosine kinase (RTK) group. FLT3 plays a crucial role in regulating hematopoiesis and is commonly found to be overexpressed in the majority of acute leukemia patients. The FLT3 pathway is pivotal for the proliferation and specialization of hematopoietic progenitor cells (HPCs). Multiple studies have demonstrated a strong association between AML and other hematologic malignancies and aberrant FLT3 pathways. Similar to other receptor tyrosine kinases (RTKs), the FLT3 receptor forms dimers upon binding to the FLT3 ligand. This leads to autophosphorylation and the subsequent activation of downstream signaling cascades, including RAS/MEK, PI3K/AKT/mTOR, and JAK/STAT [14]. These pathways have crucial functions in controlling the cell cycle, cell death, and cell specialization [15]. Cyclin-dependent kinases (CDKs) are another type of protein kinases that specifically phosphorylate serine and threonine residues. They play a vital role in regulating the course of the cell cycle and promoting cell proliferation. Blocking the activity of CDKs has been discovered to restrict the uncontrolled cellular proliferation observed in certain forms of malignancies [16]. CDK2 plays a critical role in promoting cancer progression when it is overexpressed. Therefore, suppressing the excessive activity of CDK2 may lead to the reversal of the malignant characteristics of tumor cells. CDK inhibitors, including AT-7519, flavopiridol, AMG-925, and palbociclib, are now undergoing clinical development for the treatment of several types of solid tumors and hematological malignancies. Nevertheless, the majority of CDK inhibitors were terminated during phase II trials due to significant toxicities or restricted clinical efficacy as standalone treatments.

Combination therapies that target multiple signaling pathways can potentially overcome resistance and prolong the short-term response to single FLT3 inhibitors. Although certain combination therapies have exhibited encouraging clinical outcomes, their development may be impeded by unforeseeable drug–drug interactions, pharmacokinetic characteristics, and cumulative toxicity. Multi-target drugs can address the limitations of combination therapies by simultaneously inhibiting multiple pathways. Multiple preclinical multi-target inhibitors display potential in overcoming resistance to FLT3 inhibitors [17].

**Figure 1:**
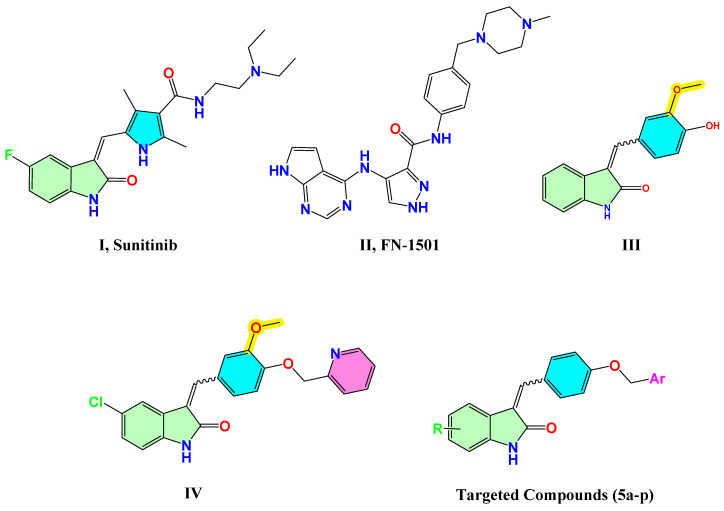
Structure of sunitinib, FN-1501, III, IV, and targeted compounds (**5a–p**).

Over the past few decades, there has been a growing focus on kinase enzyme inhibitors due to their current application in the treatment of cancer. The intricate nature of tumor formation, which encompasses numerous molecular pathways, justifies the use of multi-targeted therapy for cancer. Agents with the ability to suppress various molecular targets, affecting different cell types (such as endothelial cells, pericytes, fibroblasts, and tumor cells), are theoretically more likely to achieve success. Oxindoles, which serve as a fundamental component in the multikinase inhibitor **I**, sunitinib (Figure 1), have also been employed in the development of several kinase inhibitors. Furthermore, compound **II**, FN1501, is currently being tested in phase I/II clinical trials (NCT03690154) as a multiple-kinase inhibitor for advanced solid tumors [18]. It has demonstrated strong inhibitory effects against FLT3 (IC_50_: 0.27 nM), CDK2 (IC_50_: 2.47 nM), CDK4 (IC_50_: 0.85 nM), and CDK6 (IC_50_: 1.96 nM) [19]. Additionally, it has shown remarkable effectiveness in treating leukemia in mouse models with MV4-11 cell xenografts. Our earlier research involved examining a range of synthetic oxindole-based drugs that specifically target FLT3 and had favorable outcomes in treating AML. Compound **III**, which is based on oxindole, demonstrated remarkable effectiveness against FLT3 kinase, with IC_50_ values of 2.49 μM. Furthermore, it selectively targeted FLT3 ITD-mutated MV4-11 AML cells, with an IC_50_ of 4.3 μM after 72 h [20,21]. In addition, we have documented a sequence of substances exhibiting encouraging anticancer properties specifically against the MCF-7 breast cancer cell line. Out of these compounds, **IV** (Figure 1) demonstrated selectivity towards the MCF-7 breast cancer cell line (IC_50_ = 17.01 μM) while having no impact on the MCF-12A normal breast cell line [22].

In this current study, we further investigated the anticancer potential of oxindole-based pyridyl derivatives of compound **IV**. Instead of employing vanillin as a linker, we employed 4-hydroxybenzaldehyde. In addition, different pyridyl groups were attached to this linker to act as an allosteric binding moiety. The objective of our research is to discover novel lead compounds that exhibit promising antiproliferative activity.

## 2. Results and Discussion

### 2.1. Chemistry

**Scheme 1:**
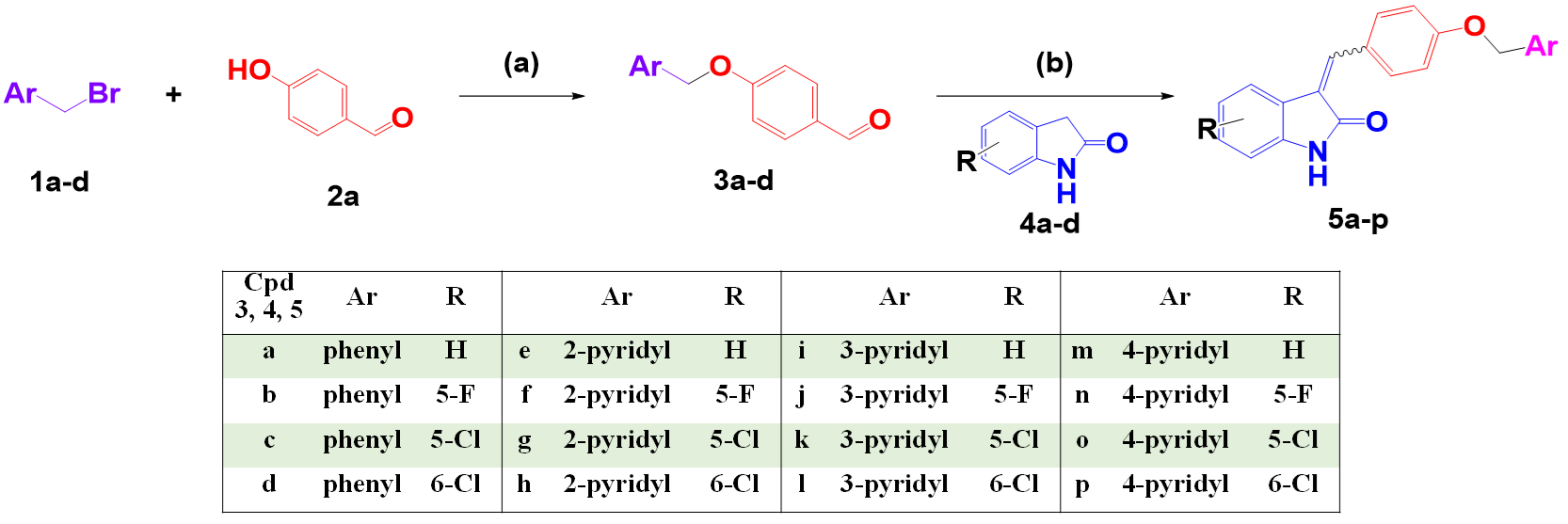
Synthetic approach of compounds **5a–p**. Reagents and conditions: (**a**) K_2_CO_3_, ACN, reflux, 2 h; (**b**) Piperidine, EtOH, reflux, 5 h.

The preparation methodologies adopted to synthesize the target compounds **5a–p** are outlined in Scheme 1. The structures of the final compounds were supported by various spectral and elemental analyses. The final compounds were obtained as a mixture of E and Z isomers, and the spectral data were recorded for the major isomer.

The outline for synthesizing the target compounds **5a–p** is illustrated in Scheme 1. The alkylated p-hydroxy benzaldehyde derivatives **3a–d** were obtained via an S_N_2 substitution reaction of various alkyl halides **1a–d** with p-hydroxy benzaldehyde’s nucleophilic hydroxyl group. The alkylated p-hydroxy benzaldehyde derivatives **3a–d** underwent a base-catalyzed Knoevenagel condensation reaction with the oxindole derivatives **4a–d** to afford the compounds **5a–p** in a good yield ranging (55–91%). The reported compounds **3a–d** were confirmed through measuring the melting point and were compared to the reported result while the newly prepared compounds **5a–p** were characterized using various spectroscopic methods. NMR data supported the success of the used route.

In the ^1^H NMR of all the target compounds, **5a–p** showed an almost characteristic singlet signal at δ = 7.6–7.9 ppm for the ethylenic protons; furthermore, the NH protons were noticed as a singlet signal at δ = 9.83–10.74 ppm. An additional singlet signal was observed at δ = 5.1–5.2 ppm for the CH_2_ protons.

Similarly, the ^13^C NMR assured the formation of the target scaffolds, showing characteristic ethylenic carbons at δ = 139–140 ppm, the aliphatic-CH_2_ carbon at δ = 67.20–71.00 ppm and carbonyl carbons of CONH groups at δ = 160.30–169.40 ppm. All the aromatic protons and carbons belonging to the oxindole, phenyl, and benzyl scaffolds were identical with the proposed structures of the compounds.

All the synthesized compounds of **5a–p** were obtained as the mixtures of *E* and *Z*-diastereomers in different ratios as identified with vinyl -H and 2′ and 6′ Hs chemical shifts (ppm) of *E* and *Z* (Table 1). The separation of these diastereomers is not possible, as reported in many previous works that acknowledged that the dynamic interconversion between the two isomers is dependent on solvent, time, and light [23,24,25,26].

^1^H NMR spectroscopy was employed to validate the assignment of the *E*/*Z*-diastereomers; in this analysis, the vinylic protons demonstrated a marginally greater chemical shift in the *Z*-diastereomer in comparison to the *E*-diastereomer. The observed variation in chemical shift can be explained by the impact of the carbonyl group situated at position 2 on the oxindole ring. Additionally, it was observed that the *E*-diastereomers exhibited a more pronounced upfield shift for the 2′ and 6′ ortho-benzylidene protons for the same underlying reason. The ^1^H NMR spectrum of compound **5l** revealed the chemical shifts associated with the principal *Z*-diastereomer. The signal was 7.80 ppm for vinylic H, 7.44 ppm for the H4 of the oxindole ring, and 8.47 ppm was the signal of H2′,6′. The corresponding signals of the minor *E*-diastereomer exhibited resonances at 7.63, 7.02, and 7.62 ppm. The ratio of *E*- to *Z*-diastereomers for each molecule was ascertained by utilizing the total integration value of the vinylic hydrogen present in both isomers as a reference. In general, it is postulated that a vinylic-H integration of 1 in both *E*- and *Z*-diastereomers signifies the complete presence of both *E*- and *Z*-diastereomers. Hence, when the upfield-shifted vinylic-H-H integration equals 0.4, it signifies that the proportion of the *E*-diastereomer is 40%. In a similar fashion, a value of 0.6 for the vinylic-H integration that is further downfield-shifted indicates a 60% *Z*-diastereomer ratio. NMR data and elemental analysis were employed to validate the purity of the target compounds.

### 2.2. High Throughput Anticancer Screening for Compounds ***5a–p***

In a comprehensive test, compounds were assayed at a single dose concentration (10^−5^ M) in the full panel of NCI-60 cancer cell lines. The detailed anti-tumor activities on the growth inhibition of NCI-60 human cancer cell lines at the single dose of 10 µM are shown in Table 2. The tested compounds showed a diverse but strong antiproliferative effect on the evaluated panel of cell lines and most of the compounds exhibited more than a 50% inhibition of tumor growth at a micromolar concentration. As revealed from the results of 16 tested compounds, six compounds with a wide range in growth percentage displayed strong growth inhibitory activity (−37.79 to 152.57%) at a 10 µM concentration (Table 3).

**Figure 2:**
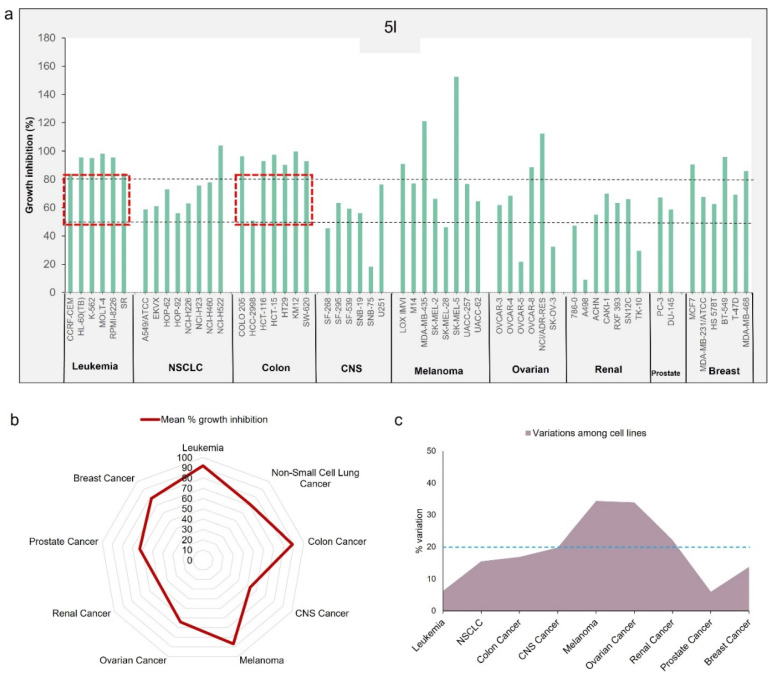
Dynamics of compound **5l** on 60 cancer cell lines from 9 different cancer panels. (**a**) % growth inhibition exerted by compound **5l** at 10 µM concentration over NCI-60 cancer cell lines. Red squares indicate the highest number of consistent inhibitory activity. (**b**) Radar representation of the inhibitory activity of compound **5l** on nine types of cancers. The red line is shaped in relation to increasing activity outward from the radar center. (**c**) Variations in **5l** inhibitory effect among cell lines in each panel of cancer in NCI-60. The turquoise line shows 20% as the threshold.

The 3-pyridyl oxindole hybrids demonstrated superior activity compared to 2-pyridyl and 4-pyridyl oxindole hybrids. The hybridization of 6-Cl oxindole with 3-pyridyl moiety in compound **5l** showed powerful activity with cytotoxic effects towards 4 cell lines and with cytostatic effects towards 44 cell lines. Moreover, the replacement of 6-Cl oxindole with 5-fluoro oxindole and the hybridization with 3-pyridyl moiety in compound **5j** gives **a** cytotoxic effect towards only 1 cell line (SK-MEL-5) and a cytostatic effect towards 32 cell lines. The hybridization of 5-Cl oxindole with 2-pyridyl moiety in compound **5g** showed powerful activity with cytotoxic effects towards 1 cell line (OVCAR-4), with a growth inhibition of 101.81% and with cytostatic effects towards 17 cell lines. Compound **5g** indicated better cytostatic activity than the hybridization of 6-Cl oxindole, 5-F oxindole, and oxindole with 2-pyridyl moiety in the series of compounds **5h**, **5f** and **5e**. Also, the replacement of 3-pyridyl moiety with 4-pyridyl moiety in compounds **5m**, **5o**, **5p**, and **5n** demonstrated only cytostatic effects towards 14, 28, 21 and 26 cell lines, respectively. On referring to the total number of sensitive cell lines for tested compounds, it has been found that most of the target compounds exhibited a broad spectrum of anti-tumor activity, covering different cancer subpanels. Amongst these sensitive cell lines, the melanoma cell line (SK-MEL-5) was found to be highly sensitive with a negative growth percentage value (lethal effect) for derivatives **5l**, **5j**, and **5n**. Also, ovarian cancer (OVCAR-4) cell lines were noticed to be the most sensitive cell lines for derivatives **5g** and **5h**. Moreover, MCF7 (breast cancer) also were proved to be the most responsible cells to compound **5o**. Moreover, compounds **5a**, **5b**, **5c** and **5d** displayed weak antiproliferative activity. Compound **5l** has been shown to have interesting growth inhibitory activity in the preliminary single dose screen, showing effectiveness toward numerous cell lines that belong to different tumor subpanels (Figure 2a). Figure 2b shows the radar chart of **5l** activity, indicating that **5l** has more overall potent activity (over 90% inhibition) in leukemia, colon cancer and melanoma. Figure 2c expresses the variations resulting from the average of activities on cell lines in each type of cancer. A lower variation in a cancer type indicates consistent inhibition across the cell lines in that panel. While the variations were observed to be over 20% in CNS, melanoma, ovarian and renal cancers, it was observed to be quite low in leukemia, colon and melanoma. Using all this data, it was then evaluated for the advanced 5-dose (10^−4^–10^−8^ M) testing mode against the full panel for **5l.**

### 2.3. Dose-Dependent Effects of Compound ***5l*** against NCI 60 Cancer Cell Panel

**Figure 3:**
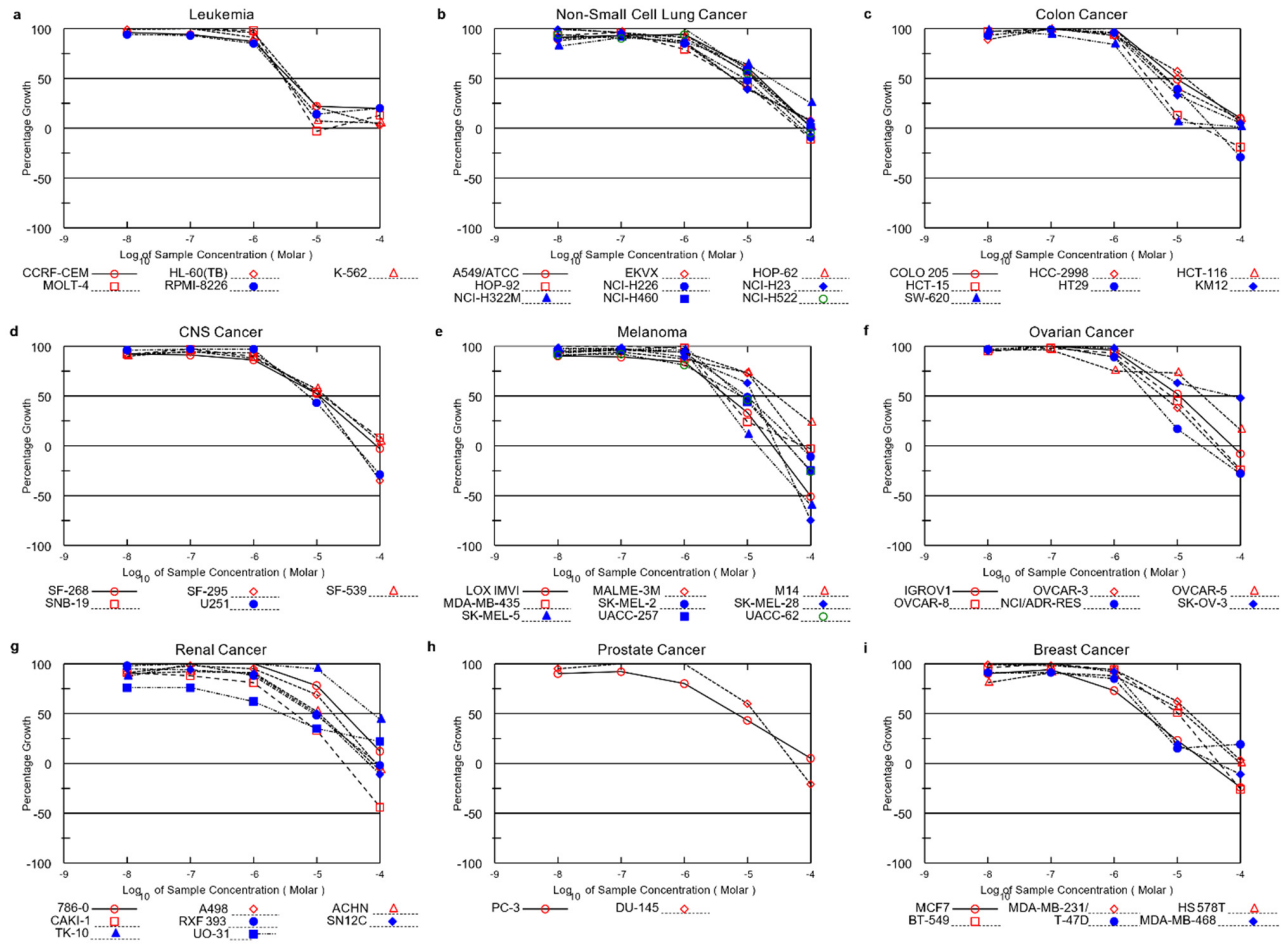
Five dose (0.01 to 100 μM) assay with compound **5l** against NCI-60 cell lines. Cells were treated with five different doses of **5l** for 48 h. Charts represent percentage growth between −100 and 100. Each cell line is indicated with different shapes and colors under the corresponding cancer chart. (**a**) Leukemia; (**b**) Non-small cell lung cancer; (**c**) Colon cancer; (**d**) CNS (central nervous system) cancer; (**e**) Melanoma; (**f**) Ovarian cancer; (**g**) Renal cancer; (**h**) Prostate cancer; (**i**) Breast cancer.

Compound **5l** has shown an interesting growth inhibitory activity in the preliminary single dose screen, showing an effectiveness toward numerous cell lines that belong to different tumor subpanels (Figure 2a–c), and the advanced compound **5l** was subjected to a 5-dose (10^−4^–10^−8^ M) testing mode against the full panel (Figure 3).

As shown in Table 4, compound **5l** showed a high antiproliferative activity at a moderate micromolar concentration on almost all evaluated human cancer types with GI_50_ values of 2.70–77.10 μM. Interestingly, compound **5l** exhibited superior micromolar activity against leukemia (MOLT-4, HL-60, CCRF-CEM, K-562, RPMI-8226), colon (HCT-15, SW-620), melanoma (LOX IMVI, MDA-MB-435, SK-MEL-5), renal cancer (CAKI-1 and UO-31), and breast cancer cell lines (MCF7, T-47D and MDA-MB-468), with a GI_50_ value range between 2.70 and 4.70 μM. In addition, compound **5l** demonstrated a high cytostatic action at a low micromolar concentration against ovarian cancer cell lines (IGROV1 and OVCAR-3) with TGI values of 3.32 and 2.26 μM. Compound **5l** showed no cytostatic effect against the left-over cancer cells (TGI > 100 μM). Conversely, compound **5l** appeared as a non-lethal agent, which has an LC_50_ greater than 100 μM for most of the examined cancer cells, excluding melanoma cell lines (LOX IMVI, SK-MEL-5, SK-MEL-28) (LC_50_ = 96.90, 65.60, and 71.50 μM), respectively, (Table 4).

Regarding the sensitivity towards various tumor cell lines, compound **5l** exhibited a potent growth inhibitory activity against the whole NCI panel, with a median GI_50_ against the full panel (MG-MID) of 12.23 μM, and effective sub-panel median GI_50_ (MG-MID) values between 3.39 and 29.43 μM. The leukemia sub-panel was the most susceptible cancer type to **5l** [GI_50_ (MG-MID) = 3.39 μM] (Table 5). Moreover, the ratio of the MG-MID of the full-panel to its individual sub-panels offers the selectivity index. Notably, **5l** showed the best selectivity index (3.61) for the leukemia cell sub-panel (Table 5).

### 2.4. Kinase Screening of Compound ***5l***

**Figure 4:**
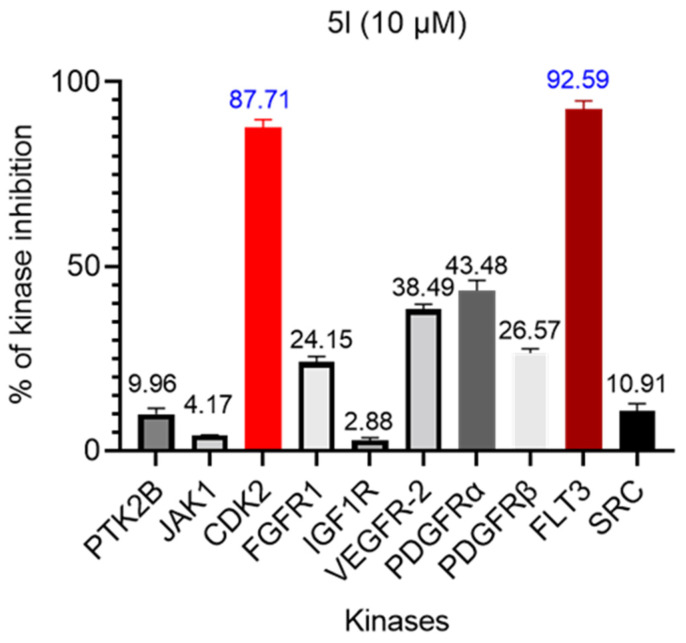
The percentage of inhibition of the tested compound **5l** (10 µM) against PTK2B, JAK1, CDK2, FGFR1, IGF1R, VEDFR-2, PDGFRα, PDGFRβ, FLT3, and SRC kinases.

The percentage of inhibition of the target compound **5l** (10 µM) was tested, versus a panel of ten kinases representing various signaling cascades including PTK2B, JAK1, CDK2, FGFR1, IGF1R, VEDFR-2, PDGFRα, PDGFRβ, FLT3, and SRC kinases, according to Z’-LYTE^®^ technology for all kinases, except where the VEGFR-2, VEGFR-2/KDR ELISA kit is used (Figure 4).

**Figure 5:**
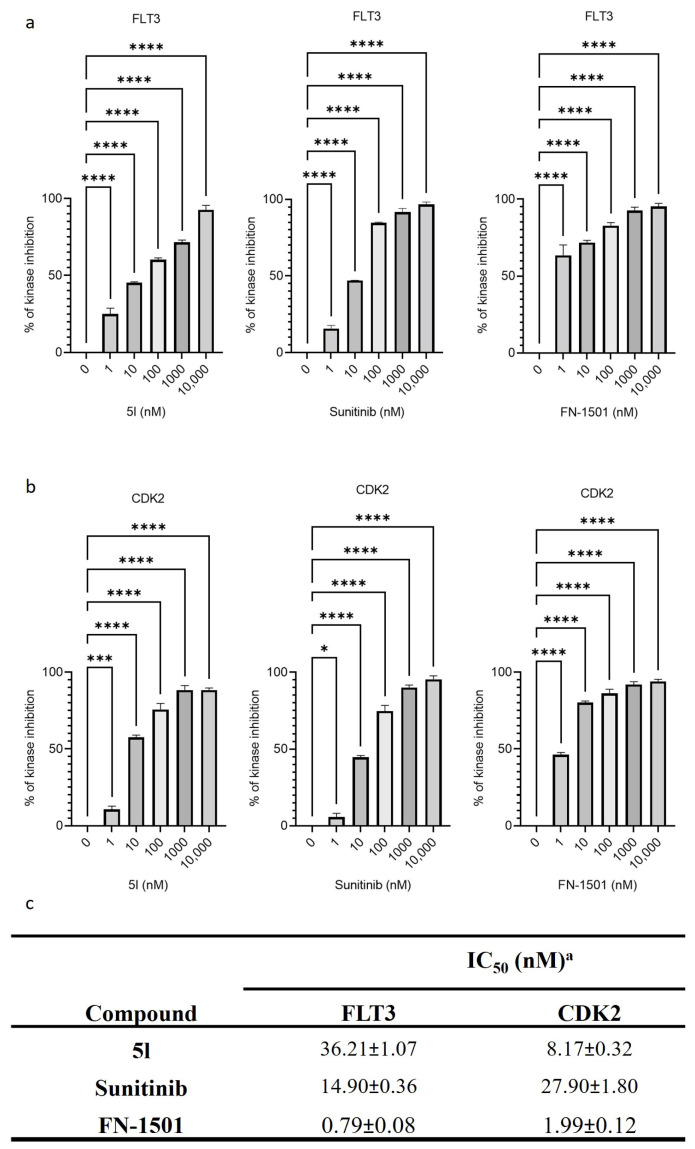
Dose titrations of **5l** compared to sunitinib and FN-1501 against (**a**) FLT3 and (**b**) CDK2 kinases from 1 to 10,000 nM. (**c**) The half maximal inhibitory concentration (IC_50_) of compound **5l** against FLT3 and CDK2 kinase activity compared to sunitinib and FN-1501. ^a^ IC_50_ values are of three separate experiments ± SD. * *p* ≤ 0.05, *** *p* ≤ 0.001, **** *p* ≤ 0.0001.

Interestingly, compound **5l** exhibited remarkable inhibition percentages on CDK2 and FLT3 kinases with values of 87.71 and 92.59%, respectively. On the contrary, it did not show any promising inhibitory percentages versus the rest. Therefore, as a consequence of the highly promising activities of compound **5l** against CDK2 and FLT3, the IC_50_ value of this compound has to be evaluated versus these two mentioned kinases. Compound **5l** was titrated for its potential IC_50_ value against FLT3 (Figure 5a) and CDK2 (Figure 5b), using sunitinib as a reference. The outcomes were reported as IC_50_ and are represented in Figure 5c, indicating that compound **5l** was presented as being a potent CDK2 inhibitor, showing a single digits nanomolar with an IC_50_ value (IC_50_ = 8.17 ± 0.32 nM), compared to sunitinib (IC_50_ of 27.90 ± 1.80 nM) and FN-1501 (IC_50_ of 0.79 ± 0.08 nM) as the reference. In contrast, compound **5l** possessed moderate FLT-3 inhibitory activity with an IC_50_ value (IC_50_ = 36.21 ± 1.07 nM), compared to sunitinib (IC_50_ of 14.90 ± 0.36 nM) and FN-1501 (IC_50_ of 1.99 ± 0.12 nM) as the reference.

### 2.5. Molecular Modeling Studies

In the endeavor to expand the arsenal of targeted treatments for colorectal cancer and acute myeloid leukemia (AML), compound **5l** emerges as an indole-2-one derivative with a structural analogy to sunitinib, a potent tyrosine kinase inhibitor. The rationale behind selecting the specific targets for molecular docking studies stems from the direct inhibitory action of sunitinib on the kinase enzymes CDK2 and FLT3 and biological kinase screening. These targets are critically involved in the mechanisms driving cancer cell proliferation and survival, making them prime candidates for therapeutic intervention. To inform and enhance the docking strategy for compound **5l**, the crystal structures of the protein CDK2 (PDB ID: 3TI1) [27] were utilized, each of which has sunitinib co-crystallized within. These structures provide a foundational understanding of the key interactions and conformations necessary for effective inhibition. For FLT3, the structure (PDB ID: 6JQR) [28] was selected to explore the binding interactions in silico as in our previous study [20], given the absence of a co-crystallized complex with sunitinib. Through these structural analyses, compound **5l** is evaluated to ascertain its potential in replicating or surpassing the inhibitory capabilities of sunitinib, thereby aiding in the design of new inhibitors that could offer enhanced therapeutic benefits for patients suffering from colorectal cancer and AML. To validate the docking protocol for compound **5l**, co-crystal ligands were re-docked into the active sites of the CDK2 and FLT3 proteins, resulting in RMSD values of 0.76 and 0.79, respectively. These low RMSD values, which were well below the commonly accepted threshold of 2 Å, indicated a high degree of accuracy in the alignment between the docked conformations and the original co-crystal structures, underscoring the effectiveness of the docking process. The *Z* isomer of compound **5l** exhibited a docking interaction energy of −8.593 kcal/mol with CDK2, whereas its *E* isomer showed a lesser interaction energy of −6.827 kcal/mol. Furthermore, the *Z* isomer of **5l** demonstrated a docking interaction energy of −9.398 kcal/mol with FLT3, compared to −8.922 kcal/mol for the *E* isomer. Given that sunitinib, as the reference compound, is also active in the *Z* configuration, these findings provide a rationale for focusing future molecular docking and dynamics studies on the *Z* isomer of **5l**.

In the molecular docking studies of compound **5l**, a comprehensive interaction profile has emerged, delineating the compound’s engagement with several key proteins. These interactions suggest that compound **5l** could serve as a potent inhibitor, as evidenced by the specific binding modalities elucidated for each target protein.

**Figure 6:**
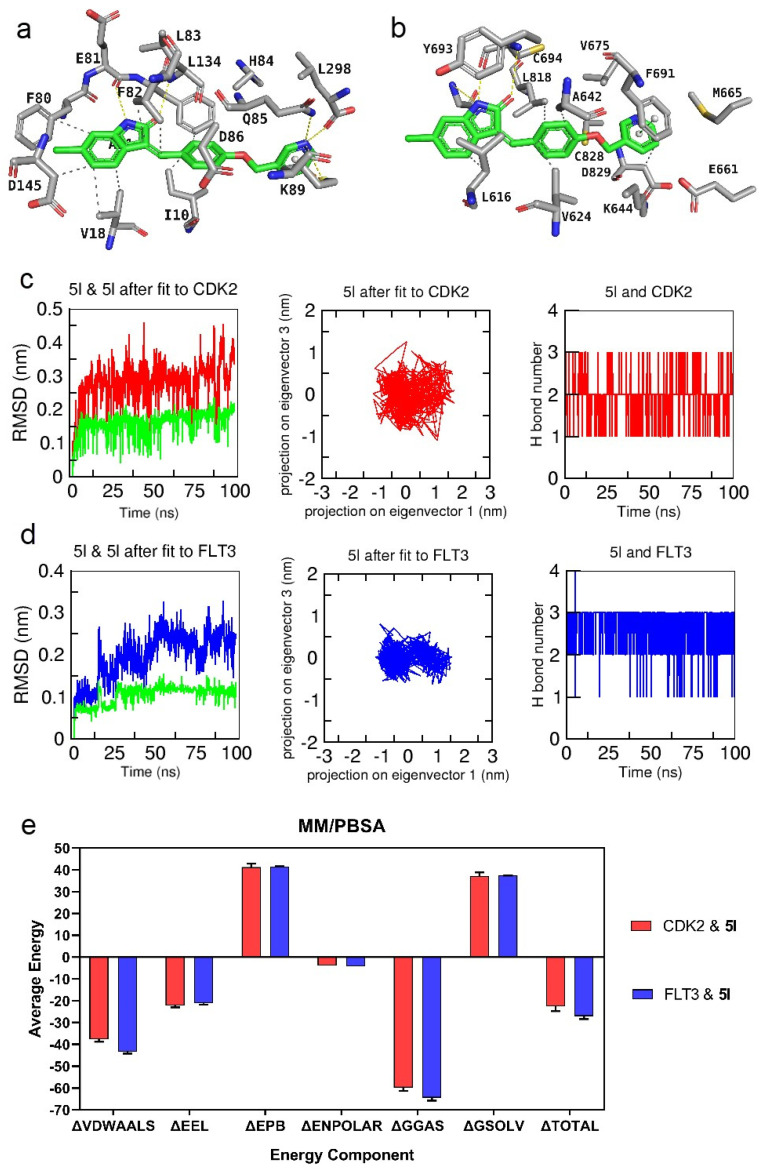
Docking interactions and dynamics simulations of compound **5l** with kinase targets related to colorectal cancer and AML. (**a**) The conformation of compound **5l** within CDK2 (PDB ID: 3TI1) active site, showing interaction points. (**b**) FLT3 (PDB ID: 6JQR) in complex with compound **5l**, presenting the compound’s orientation and contact regions. (**c**,**d**) Molecular dynamics simulation of compound **5l** with kinase targets over 100 ns. RMSD profiles for **5l** for each target (green), CDK2 (red line) and FLT3 (blue line) complexes, indicating deviations from initial conformations. Conformational space exploration of **5l** with each kinase, represented using PCA. Hydrogen bond count over the simulation period, reflecting interaction consistency for each kinase–ligand complex. (**e**) MM/PBSA binding free energy components for compound **5l** with kinase targets. The bar graph summarizes the van der Waals, electrostatic, and solvation energy contributions to the total binding free energy (ΔTOTAL) of **5l** with CDK2 and FLT3.

Starting with CDK2, as highlighted in Figure 6a, the docking profile is characterized initially by the formation of hydrogen bonds with Glu81 (2.44 Å), Leu83 (1.94 Å), Gln85 (2.25 Å), and Lys89 (2.47 Å). These interactions are complemented by hydrophobic contacts with residues Ile10, Val18, Phe80, Phe82, Leu134, and Asp145. Notably, a π-cation interaction with Lys89 is observed, further solidifying the ligand’s favorable orientation within the binding pocket. Finally, with FLT3, as illustrated in Figure 6b, compound **5l** establishes hydrogen bonds with Cys694 (1.98 Å and 2.47 Å) and Asp829 (2.57 Å). This bonding network is supported by hydrophobic interactions with Leu616, Val624, Ala642, Leu818, and Asp829. Additionally, the π-stacking with Phe691 contributes significantly to the binding stability, indicating a highly specific interaction scheme.

In the field of computational drug discovery, molecular dynamics (MD) simulations are invaluable for providing a dynamic perspective on the interactions between drug candidates and their target proteins. Such simulations offer detailed insight into the conformational flexibility, stability, and binding efficacy of compounds at an atomic scale. Within this framework, a 100 ns MD simulation was executed to investigate the binding dynamics of compound **5l** with two pivotal kinase targets—FLT3 and CDK2—each playing a crucial role in cancer pathophysiology and thus representing significant therapeutic targets. The primary goal of the simulation was to determine the stability of compound **5l** within the active sites of these kinases and to delineate the nature of its binding over the simulated timeframe. In addition, to compare compound **51** with sunitinib, the crystal structure of sunitinib with CDK2 and the protein–ligand complexes obtained by docking sunitinib to FLT3 were also compared using MD simulation. Through meticulous analyses of root-mean-square deviation (RMSD), principal component analysis (PCA), and hydrogen bond dynamics, valuable insights into the binding stability and conformational adaptations of compound **5l** were obtained, which are vital for its potential efficacy as a kinase inhibitor.

The RMSD findings revealed that the FLT3-**5l** complex displayed remarkable stability, with RMSD values persistently under 0.3 nm, denoting a consistently tight binding throughout the simulation (Figure 6d). In contrast, the CDK2-**5l** complex had slightly higher RMSD values, reaching peaks of up to 0.4 nm, suggesting a stable interaction with a degree of flexibility (Figure 6c). Complementary PCA analysis (Figure 6c,d) reflected these observations, with the FLT3-**5l** complex demonstrating a densely clustered pattern, indicative of a stable interaction with limited conformational change. The CDK2-**5l** complex exhibited a more scattered PCA profile, implying a broader range of ligand movements. The hydrogen bond analysis (Figure 6c,d) further substantiated the stability of the FLT3-**5l** interaction, with a relatively constant hydrogen bond count. The CDK2-**5l** complex, while maintaining a consistent number of hydrogen bonds, displayed greater variability. Moreover, according to sunitinib’s RMSD profile in the MD simulation (Figure S1), compound **5l** was found to be more stable than sunitinib with the target proteins, CDK2 and FLT3.

The stability of compound **5l** within the binding pockets of CDK2 and FLT3 is dynamically showcased through animation videos derived from the MD trajectories (Videos S1 and S3). These visualizations add a layer of depth to our understanding by displaying the robustness of the hydrogen bonding interactions in real time. They particularly highlight the persistence of hydrogen bonds with the indole-2-one moiety of compound **5l**, while also noting the variable nature of the interactions with the pyridine moiety across the simulation timeframe. Additionally, for the comparison of compound **5l** with sunitinib, the animation video obtained from the MD simulation of sunitinib performed with CDK2 is also presented in Videos S2 and S4, respectively.

Further to the dynamic analyses, the Molecular Mechanics/Poisson-Boltzmann Surface Area (MM/PBSA) method was employed to calculate the binding free energies of the complexes, providing an estimate of the binding affinity of compound **5l** to the kinases. MM/PBSA calculations were performed on 200 frames extracted between 80 and 100 ns of the simulation, resulting in the plot shown in Figure 6e. This plot offers a quantitative view of the energetic components contributing to the binding affinity, such as van der Waals interactions, electrostatic contributions, and solvation effects, thereby complementing the qualitative insights from the MD simulation. Integrating the MM/PBSA free energy calculations into our analysis further refines our understanding of the interaction between compound **5l** and the kinase targets. The MM/PBSA approach is instrumental in quantifying the binding affinities through various energetic contributions, offering a complementary perspective to the dynamic observations from MD simulations. According to MM/PBSA data, the binding free energies (ΔGMM/PBSA) are −22.52 ± 2.20 kcal/mol for CDK2-**5l**, and −27.09 ± 1.27 kcal/mol for FLT3-**5l**, indicating a hierarchy of binding affinities, with FLT3-**5l** exhibiting the strongest interaction. These values reveal that the FLT3-**5l** complex possesses the most favorable binding affinity with a ΔGMM/PBSA of −27.09 kcal/mol, which correlates with the lower RMSD values and the tighter clustering observed in PCA. The standard deviation of ±1.27 kcal/mol also indicates a consistent binding affinity throughout the sampled frames. The CDK2-**5l** complex, while displaying a ΔGMM/PBSA of −22.52 kcal/mol, shows the largest standard deviation, reflecting a broader range of interaction energies which may be indicative of the flexibility observed in the MD simulation. Overall, the MM/PBSA calculations align well with the stability trends observed in the MD simulations. The FLT3-**5l** complex stands out with the highest binding affinity and the most stable interaction, making it a promising candidate for further drug development efforts aimed at FLT3 inhibition. The consistency and favorability of these interactions provide a quantitative foundation for the potential efficacy of compound **5l** as a therapeutic agent, confirming the qualitative insights gained from the MD simulation and subsequent analysis.

### 2.6. Physicochemical, ADME, Pharmacokinetic Characteristics and Drug-Likeness Forecast

The Swiss Institute of Bioinformatics (SIB) hosts the freely accessible SwissADME tool, which serves as an invaluable computational resource for globally assessing the pharmacokinetic profiles and drug-likeness of small molecular scaffolds. This analysis is essential for confirming that newly synthesized compounds exhibit promising physiological and pharmacokinetic properties, thereby streamlining the drug development process in terms of cost, time, and efficacy [29].

Compound **5l**, a potent antiproliferative agent within the 3-substituted oxindole series, was characterized by its strong hydrophilicity, adequate gastrointestinal (GI) tract absorption, and competent blood–brain barrier (BBB) penetration. These attributes are quantified by a predicted log Po/w value of 3.10. When evaluated against the Lipinski and Veber rules for drug-likeness, compound **5l** demonstrated a profile marked by increased lipophilicity and low solubility. Notably, the compound exhibits a degree of unsaturation outside the optimal range, with a fraction of sp^3^ hybridized carbons (Fraction Csp3) value of 0.05, which is below the preferred threshold of 0.25, indicating potential challenges for its drug-likeness. Despite this, compound **5l** is poorly soluble in water and is classified within the category of hERG I inhibitors, which are known to pose cardiotoxic risks, as well as being associated with AMES toxicity and skin sensitization. In contrast, it does not exhibit hepatotoxic or hERG II channel inhibition, which is often related to adverse drug reactions.

**Figure 7:**
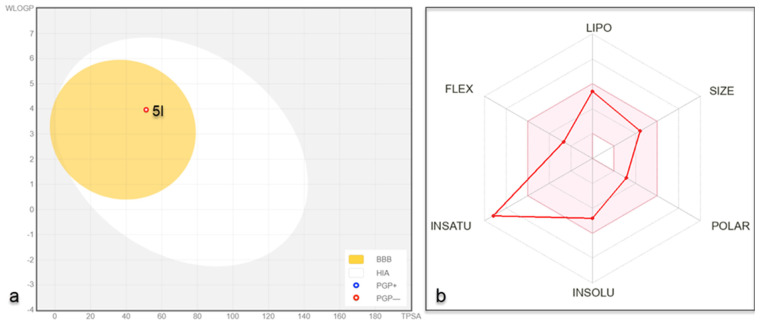
ADME estimation of active compound **5l.** (**a**) The BOILED-Egg plot showcases the predicted GI absorption and BBB permeability for **5l**, aligning with non-P-glycoprotein substrate characteristics (PGP−), and (**b**) the bioavailability radar chart, with the pink area depicting the optimal property values and the red lines representing the properties of **5l**, which largely fall within the desirable range, indicating good oral bioavailability.

The Brain or Intestinal Estimated permeation method (BOILED-Egg) was employed to graphically project and evaluate compound **5l**’s passive GI absorption and BBB penetration [30]. As visualized in Figure 7a, compound **5l** is aptly situated within the human intestinal absorption window and demonstrates BBB permeability, while also being identified as a non-substrate for P-glycoprotein (PGP-) efflux transporters, which suggests a reduced likelihood of resistance via an active efflux by cancer cells. Figure 7b further highlights the pharmacokinetic predictions where the lipophilicity (WLOGP) and Total Polar Surface Area (TPSA) are utilized to estimate the compound’s pharmacokinetic behavior. Red points in the bioavailability radar chart for compound **5l** are almost entirely encompassed within the ideal property range (pink area), which indicate a favorable oral bioavailability.

## 3. Materials and Methods

### 3.1. Chemistry

#### 3.1.1. General

All chemicals and organic solvents utilized to synthesize the desired compounds were of commercial grade, purchased from the Alfa Aesar (Cairo, Egypt), Sigma Aldrich (Darmstadt, Germany), and El-gomhoria (Cairo, Egypt) companies for pharmaceutical use, and were used without further purification. Reaction progress was monitored using pre-coated TLC plates (Kiesslgel 60 F_254_, Merck, Rahway, NJ, USA) and detecting spots was performed via UV lamp at a wavelength of 254 nm. Melting points were measured on as Stuart Electro-Thermal apparatus and recorded without correction. ^1^HNMR was carried out on a JOEL JNM-ECA400 (^1^HNMR: 400 MHz, ^13^CNMR: 100 MHz) spectrophotometer at Zagazig University, Egypt. Chemical shifts were recorded in parts per million (ppm), compared to the standard TMS using deuterated DMSO (δ: 2.5 ppm) as a solvent, and the coupling constant (*J*) was measured in hertz (Hz). Multiplicity was designated as: s, singlet; d, doublet; t, triplet; dd, doublet of doublet; and m for multiplet. Elemental analyses were performed on a Perkin Elmer 2400 CHN elemental analyzer at the regional center for mycology and biotechnology, Al-Azhar University, Nasr City, Cairo, Egypt.

#### 3.1.2. General Procedure for the Synthesis of Compounds **3a–d**

A mixture of the appropriate alkyl halide (**3a–d**, 1 mmol), *p*-hydroxy benzaldehyde **2a** (1 mmol, 122 mg), and potassium carbonate (1 mmol, 138 mg) was heated to reflux in acetonitrile (20 mL) for 2 h. The reaction progress was monitored via TLC, then the reaction mixture was concentrated under reduced pressure and the remaining mixture was extracted using ethyl acetate (3 × 30 mL) and washed with water (3 × 30 mL). The organic layer was collected and dried over anhydrous sodium sulfate, the solvent was filtered off and then evaporated to yield compounds **3a–d** that were used for further reactions without additional purification [31].

**4-(Benzyloxy) benzaldehyde (3a)**

White solid; 71% yield (160 mg); mp = 67–69 °C, reported mp: 68–70 °C; R*_f_* = 0.3 (pet.ether:EtOAc, 3:1) [32,33].

**4-(Pyridin-2-ylmethoxy) benzaldehyde (3b)**

White solid; 53% yield (110 mg); mp = 95–98 °C, reported mp: 93–95 °C; R*_f_* = 0.3 (pet.ether:EtOAc, 3:1) [34].

**4-(Pyridin-3-ylmethoxy) benzaldehyde (3c)**

Yellow solid; 53% yield (110 mg); mp = 75–78 °C, reported mp: 76–78 °C; R*_f_* = 0.3 (pet.ether:EtOAc, 3:1) [35].

**4-(Pyridin-4-ylmethoxy) benzaldehyde (3d)**

White solid; 53% yield (110 mg); mp = 99–103 °C, reported mp: 102–104 °C; R*_f_* = 0.3 (pet.ether:EtOAc, 3:1) [36].

#### 3.1.3. General Procedure for the Synthesis of Compounds **5a–p**

Indolin-2-ones (**4a–d**,1 mmol) were added to a solution of appropriate alkylated *p*-hydroxy benzaldehyde derivatives (**3a–d**, 1 mmol) in EtOH (10 mL). A catalytic amount of piperidine was added as base, and the reaction mixture was heated at reflux for 5 h. Reaction progress was monitored using TLC. After completion, the reaction mixture was allowed to cool. The formed precipitate was filtered and washed with cold EtOH then recrystallized using absolute ethanol to yield compounds **5a–p** [37].

**(*E*/*Z*)-3-[4-(Benzyloxy) benzylidene] indolin-2-one (5a)**

Yellow powder; 89% yield (125 mg); mp: 220–223 °C, R*_f_* = 0.42 (pet. ether:EtOAc, 1:1). ^1^H NMR (400 MHz, DMSO-*d_6_*) δ: *E*:*Z* ratio = 3:97

*Z*-diastereomer: 10.59 (s, 1H, NH), 8.49 (d, *J* = 8.9 Hz, 2H, Ar-H), 7.75 (s, 1H, C=CH), 7.69 (d, *J* = 7.5 Hz, 1H, Ar-H), 7.50 (d, *J* = 7.2 Hz, 2H, Ar-H), 7.44 (t, *J* = 7.3 Hz, 2H, Ar-H), 7.37 (t, *J* = 7.2 Hz, 1H, Ar-H), 7.20 (t, *J* = 7.3 Hz, 1H, Ar-H), 7.13 (d, *J* = 8.9 Hz, 2H, Ar-H), 7.00 (t, *J* = 7.4 Hz, 1H, Ar-H), 6.83 (d, *J* = 7.7 Hz, 1H, Ar-H), 5.21 (s, 2H, CH_2_).

*E*-diastereomer: 10.59 (s, 1H, NH), 8.47 (d, *J* = 8.9 Hz, 2H, Ar-H), 7.69 (t, *J* = 7.2 Hz,1H, Ar-H), 7.67 (d, *J* = 7.5 Hz, 1H, Ar-H), 7.48 (d, *J* = 7.2 Hz, 2H, Ar-H), 7.40 (t, *J* = 7.3 Hz, 2H, Ar-H), 7.34 (s, 1H, C=CH), 7.17 (t, *J* = 7.3 Hz, 1H, Ar-H), 7.11 (d, *J* = 8.9 Hz, 2H, Ar-H), 6.96 (t, *J* = 7.4 Hz, 1H, Ar-H), 6.81 (d, *J* = 7.7 Hz, 1H, Ar-H), 5.21 (s, 2H, CH_2_).

^13^C NMR (100 MHz, DMSO-*d_6_*) δ:

*Z*-diastereomer: 167.37, 160.25, 140.26, 136.75, 136.68, 134.38, 128.48, 128.25, 127.98, 127.84, 127.17, 125.33, 124.13, 120.89, 119.26, 114.55, 109.19, 69.38.

*E*-diastereomer: 167.37, 160.25, 140.26, 136.75, 136.68, 134.38, 128.48, 128.25, 127.98, 127.84, 127.17, 125.33, 124.13, 120.89, 119.26, 114.55, 109.19, 69.38. 

Anal. Calcd. For C_22_H_17_NO_2_ (327.13): C, 80.71; H, 5.23; N, 4.28, found; C, 80.36; H, 5.42; N, 4.31.

**(*E*/*Z*)-3-[4-(Benzyloxy) benzylidene]-5-fluoroindolin-2-one (5b)**

Yellow powder; 82% yield (91.3 mg); mp: 244–248 °C, R*_f_* = 0.41 (pet. ether:EtOAc, 1:1). ^1^H NMR (400 MHz, DMSO-*d*_6_) δ: *E*:*Z* ratio = 15:85

*Z*-diastereomer: 10.59 (s, 1H, NH), 8.50 (d, *J* = 8.9 Hz, 2H, Ar-H), 7.82 (s, 1H, C=CH), 7.62 (t, *J* = 9.1, 1H, Ar-H), 7.49–7.47 (m, 2H, Ar-H), 7.44–7.41 (m, 2H, Ar-H), 7.38–7.35 (m, 1H, Ar-H), 7.14 (d, *J* = 8.9 Hz, 2H, Ar-H), 7.04–7.01 (m, 1H, Ar-H), 6.80 (t, *J* = 8.5 Hz, 1H, Ar-H), 5.21 (s, 2H, CH_2_).

*E*-diastereomer: δ 10.47 (s, 1H, NH), 8.47 (d, *J* = 8.9 Hz, 2H, Ar-H), 7.82 (m, 1H, Ar-H), 7.59 (t, *J* = 9.1, 1H, Ar-H), 7.47–7.45 (m, 2H, Ar-H), 7.40–7.38 (m, 2H, Ar-H), 7.33 (s, 1H, C=CH), 7.12 (d, *J* = 8.9 Hz, 2H, Ar-H), 7.00–6.96 (m, 1H, Ar-H), 6.77 (t, *J* = 8.5 Hz, 1H, Ar-H), 5.21 (s, 2H, CH_2_).

^13^C NMR (100 MHz, DMSO-*d*_6_) δ:

*Z*-diastereomer: 167.49, 160.65, 160.00, 138.57, 137.76, 136.67, 136.48, 134.76, 131.68, 128.56, 128.07, 127.93, 127.91, 127.00, 126.46, 115.26, 114.71, 69.54.

*E*-diastereomer: 168.97, 159.17, 158.40, 156.84, 139.02, 134.76, 131.68, 128.56, 127.93, 123.83, 122.22, 115.90, 114.53, 110.73, 110.01, 109.00, 106.89, 69.48.

Anal. Calcd. For C_22_H_16_FNO_2_ (345.12): C, 76.51; H, 4.67; N, 4.06; found: C, 76.23; H, 4.98; N, 4.20.

**(*E*/*Z*)-3-[4-(Benzyloxy) benzylidene]-5-chloroindolin-2-one (5c)**

Yellow powder; 65% yield (87 mg); mp = 214–216 °C, R*_f_* = 0.44 (pet. ether:EtOAc, 1:1). ^1^H NMR (400 MHz, DMSO-*d*_6_) δ: *E*:*Z* ratio = 35:65

*Z*-diastereomer: 10.71 (s, 1H, NH), 8.49 (d, *J* = 8.9 Hz, 2H, Ar-H), 7.89 (s, 1H, C=CH), 7.65–7.54 (m, 1H, Ar-H), 7.49–7.45 (m, 2H, Ar-H), 7.39 (t, *J* = 7.2 Hz, 2H, Ar-H), 7.35–7.32 (m, 1H, Ar-H), 7.19 (t, *J* = 8.4 Hz, 2H, Ar-H), 7.14 (d, *J* = 8.9 Hz, 1H, Ar-H), 6.90–6.88 (t, *J* = 8.3 Hz, 1H, Ar-H), 5.20 (s, 2H, CH_2_).

*E*-diastereomer: 10.71 (s, 1H, NH), 8.62 (d, *J* = 8.9 Hz, 2H, Ar-H), 7.93–7.85 (m, 1H, Ar-H), 7.75–7.66 (m, 1H, Ar-H), 7.56–7.50 (m, 2H, Ar-H), 7.41 (t, *J* = 7.2 Hz, 2H, Ar-H), 7.33 (s, 1H, C=CH), 7.24 (t, *J* = 8.4 Hz, 2H, Ar-H), 7.12 (d, *J* = 8.9 Hz, 1H, Ar-H), 6.82–6.80 (t, *J* = 8.3 Hz, 1H, Ar-H), 5.20 (s, 2H, CH_2_).

^13^C NMR (101 MHz, DMSO-*d*_6_) δ:

*Z*-diastereomer: 167.14, 160.66, 138.87, 138.77, 136.61, 134.77, 131.62, 128.49, 128.00, 127.89, 127.86, 125.28, 124.87, 119.31, 115.17, 114.66, 110.53, 69.48.

*E*-diastereomer: 168.56, 159.98, 141.43, 137.90, 131.62, 129.97,128.49, 127.57, 127.32, 127.00, 126.40, 125.28, 124.84, 123.00, 122.81, 121.43, 111.39, 69.43.

Anal. Calcd. For C_22_H_16_ClNO_2_ (361.09): C, 73.03; H, 4.46; N, 3.87. found: C, 72.71; H, 4.17; N, 3.92.

**(*E*/*Z*)-3-[4-(Benzyloxy) benzylidene]-6-chloroindolin-2-one (5d)**

Yellow powder; 80% yield (94 mg); mp: 259–261 °C, R*_f_* = 0.43 (pet. ether:EtOAc, 1:1). ^1^H NMR (400 MHz, DMSO-*d*_6_) δ: *E*:*Z* ratio = 15:85

*Z*-diastereomer: 10.74 (s, 1H, NH), 8.47 (d, *J* = 8.9 Hz, 2H, Ar-H), 7.80 (s, 1H, C=CH), 7.70 (d, *J* = 8.2 Hz, 1H, Ar-H), 7.49–7.45 (d, *J* = 7.1 Hz, 2H, Ar-H), 7.41 (t, *J* = 7.3 Hz, 2H, Ar-H), 7.40–7.37 (t, *J* = 7.1 Hz, 1H, Ar-H), 7.14 (d, *J* = 8.9 Hz, 2H, Ar-H), 7.06 (d, *J* = 1.8 Hz, 1H, Ar-H), 6.83 (d, *J* = 1.8 Hz, 1H, Ar-H), 5.21 (s, 2H, CH_2_).

*E*-diastereomer: 10.74 (s, 1H, NH), 8.45 (d, *J* = 8.9 Hz, 2H, Ar-H), 7.80 (t, *J* = 7.1 Hz, 1H, Ar-H), 7.68 (d, *J* = 8.2 Hz, 1H, Ar-H), 7.44–7.42 (d, *J* = 7.1 Hz, 2H, Ar-H), 7.36 (t, *J* = 7.3 Hz, 2H, Ar-H), 7.30 (s, 1H, C=CH), 7.11 (d, *J* = 8.9 Hz, 2H, Ar-H), 7.02 (d, *J* = 1.8 Hz, 1H, Ar-H), 6.82 (d, *J* = 1.8 Hz, 1H, Ar-H), 5.21 (s, 2H, CH_2_).

^13^C NMR (100 MHz, DMSO-*d*_6_) δ:

*Z*-diastereomer: 167.24, 160.51, 141.32, 137.90, 136.62, 134.58, 132.24, 131.66, 128.48, 127.99, 127.85, 127.00, 124.32, 122.91, 120.66, 114.62, 109.13, 69.41.

*E*-diastereomer: 167.24, 141.32,137.90, 136.62, 134.58, 133.61, 132.24, 131.66, 128.48, 127.99,127.85, 124.32, 122.91, 120.66, 115.15, 114.62, 109.13, 69.41.

Anal. Calcd. For C_22_H_16_ClNO_2_ (361.09): C, 73.03; H, 4.46; N, 3.87. found: C, 72.76; H, 4.17; N, 3.68.

**(*E*/*Z*)-3-[4-(Pyridin-2-ylmethoxy] benzylidene) indolin-2-one (5e)**

Orange powder; 76% yield (90 mg); mp: 221–224 °C, R*_f_* = 0.42 (pet. ether:EtOAc, 1:1). ^1^H NMR (400 MHz, DMSO-*d*_6_) δ: *E*:*Z* ratio = 31:69

*Z*-diastereomer: 10.58 (s, 1H, NH), 8.60 (d, 1H, Ar-H), 8.48 (d, *J* = 8.9 Hz, 1H, Ar-H), 7.90–7.86 (m, 1H, Ar-H), 7.78–7.74 (m, 1H, Ar-H), 7.68 (t, *J* = 8.5 Hz, 1H, Ar-H), 7.58–7.56 (t, *J* = 6.4 Hz, 1H, Ar-H), 7.36 (s, 1H, C=CH), 7.26 –7.18 (m, 3H, Ar-H), 6.99–6.97 (t, *J* = 7.5 Hz, 1H, Ar-H), 6.91–6.88 (m, 1H, Ar-H), 6.82 (d, *J* = 7.7 Hz, 1H, Ar-H), 5.27 (s, 2H, CH_2_).

*E*-diastereomer: 10.56 (s, 1H, NH), 8.59 (d, 1H, Ar-H), 8.46 (d, *J* = 8.9 Hz, 1H, Ar-H), 7.85–7.81 (m, 1H, Ar-H), 7.73–7.71 (m, 3H, Ar-H), 7.64 (t, *J* = 8.5 Hz, 1H, Ar-H), 7.55–7.51 (t, *J* = 6.4 Hz, 1H, Ar-H), 7.35–7.31 (m, 1H, Ar-H), 7.22 ((s, 1H, C=CH), 6.97–6.95 (t, *J* = 7.5 Hz, 1H, Ar-H), 6.87–6.84 (m, 1H, Ar-H), 6.80 (d, *J* = 7.7 Hz, 1H, Ar-H), 5.27 (s, 2H, CH_2_).

^13^C NMR (100 MHz, DMSO-*d*_6_) δ:

*Z*-diastereomer: 168.89, 159.41, 156.31, 149.24, 142.70, 137.07, 135.87, 134.39, 131.53, 129.75, 127.04, 125.80, 123.11, 121.85, 121.06, 115.08, 110.05, 109.21, 70.57.

*E*-diastereomer: 168.89, 160.01, 159.41, 149.19, 142.70, 140.29, 137.05, 136.67, 128.30, 125.31, 124.28, 121.12, 120.91, 119.29, 114.54, 110.05, 109.21, 70.40, 56.04.

Anal. Calcd. For C_21_H_16_N_2_O_2_ (328.37) C, 76.81; H, 4.91; N, 8.53. found: C, 77.40; H, 5.21; N, 8.14.

**(*E*/*Z*)-5-fluoro-3-[4-(pyridin-2-ylmethoxy) benzylidene]indolin-2-one (5f)**

Yellow powder; 93% yield (170 mg); mp: 226–228 °C, R*_f_* = 0.40 (pet. ether:EtOAc, 1:1). ^1^H NMR (400 MHz, DMSO-*d*_6_) δ: *E*:*Z* ratio = 33:67

*Z*-diastereomer: 10.59 (s, 1H, NH), 8.61–8.59 (d, 1H, Ar-H), 7.90–7.86 (m, 2H, Ar-H), 7.73 (d, *J* = 8.6 Hz, 2H, Ar-H), 7.65 (s, 1H, C=CH), 7.43–7.36 (m, 2H, Ar-H), 7.22 (t, *J* = 8.9 Hz, 2H, Ar-H), 7.16–7.01 (m, 1H, Ar-H), 6.87–6.82 (t, *J* = 8.5 Hz, 1H), 5.29 (s, 2H, CH_2_). 

*E*-diastereomer: 10.58 (s, 1H, NH), 8.49–8.47 (d, 1H, Ar-H), 7.85–7.81 (m, 2H, Ar-H), 7.70 (d, *J* = 8.6 Hz, 2H, Ar-H), 7.58–7.53 (m, 2H, Ar-H), 7.38 (s, 1H, C=CH), 7.20 (t, *J* = 8.9 Hz, 2H, Ar-H), 7.00–6.96 (m, 1H, Ar-H), 6.80–6.77 (t, *J* = 8.5 Hz, 1H), 5.29 (s, 2H, CH_2_).

^13^C NMR (100 MHz, DMSO-*d*_6_) δ

*Z*-diastereomer: 168.86, 167.41, 160.37, 159.72, 156.28, 156.24, 149.20, 138.43, 137.60, 137.07, 134.70, 131.64, 127.14, 126.62, 123.12, 121.87, 115.22, 114.65, 70.49.

*E*-diastereomer: 158.34, 156.78, 156.00, 139.02, 137.43, 136.49, 134.70, 123.89, 122.06, 120.75, 116.10, 115.87, 114.26, 110.65, 109.93, 108.99, 106.89, 70.49, 52.16.

Anal. Calcd. For C_21_H_15_FN_2_O_2_ (346.36) C, 72.82; H, 4.37; N, 8.09. found: C, 73.13; H, 4.08; N, 8.20.

**(*E*/*Z*)-5-Chloro-3-[4-(pyridin-2-ylmethoxy) benzylidene] indolin-2-one (5g)**

Yellow powder; 89% yield (150 mg); mp: 219–220 °C, R*_f_* = 0.35 (pet. ether: EtOAc, 1:1). ^1^H NMR (400 MHz, DMSO-*d*_6_) δ: *E*:*Z* ratio = 23:77

*Z*-diastereomer: 10.71 (s, 1H, NH), 8.61–8.59 (d, 1H, Ar-H), 8.47 (d, 1H, Ar-H), 7.93–7.85 (m, 2H, Ar-H), 7.66 (s, 1H, C=CH), 7.59–7.55 (t, *J* = 8.8 Hz, 2H, Ar-H), 7.41–7.36 (s, 1H, Ar-H), 7.29–7.24 (t, *J* = 8.3 Hz, 1H, Ar-H), 7.16 (t, *J* = 8.9 Hz, 2H, Ar-H), 6.90–6.87 (t, *J* = 8.3 Hz, 1H, Ar-H), 5.28 (s, 2H, CH_2_).

*E*-diastereomer: 10.71 (s, 1H, NH), 8.50–8.47 (t, *J* = 8.8 Hz, 2H, Ar-H), 7.84–7.80 (m, 2H, Ar-H), 7.69–7.63 (m, 2H, Ar-H), 7.57 (s, 1H, C=CH), 7.35–7.32 (s, 1H, Ar-H), 7.23–7.20 (t, *J* = 8.3 Hz, 1H, Ar-H), 7.14 (t, *J* = 8.9 Hz, 2H, Ar-H), 6.82–6.80 (t, *J* = 8.3 Hz, 1H, Ar-H), 5.28 (s, 2H, CH_2_).

^13^C NMR (100 MHz, DMSO-*d*_6_) δ:

*Z*-diastereomer: 187.44, 176.19, 166.09, 148.62, 142.12, 137.19, 134.07, 132.62, 131.88, 130.74, 129.99, 123.89, 122.72, 118.88, 111.21, 107.14, 106.09, 52.05, 49.10.

*E*-diastereomer: 187.44, 176.19, 176.19, 166.09, 148.62, 142.12, 137.19, 134.07, 132.62, 131.88, 130.74, 129.99, 123.89, 122.72, 118.88, 111.21, 107.14, 106.09, 52.05, 49.10.

Anal. Calcd. For C_21_H1_5_ClN_2_O_2_ (362.81) C, 69.52; H, 4.17; N, 7.72. found: C, 69.24; H, 4.02; N, 7.58.

**(*E*/*Z*)-6-Chloro-3-[4-(pyridin-2-ylmethoxy) benzylidene]indolin-2-one (5h)**

Yellow powder; 90% yield (213 mg); mp: 260–263 °C, R*_f_* = 0.38 (pet. ether:EtOAc, 1:1). ^1^H NMR (400 MHz, DMSO-*d*_6_) δ: *E*:*Z* ratio = 47:53

*Z*-diastereomer: 10.74 (s, 1H, NH), 8.64–8.60 (m, 2H, Ar-H), 8.47 (d, *J* = 8.9 Hz, 2H, Ar-H), 7.81 (s, 1H, C=CH), 7.72 (t, *J* = 7.7 Hz, 1H, Ar-H), 7.57 (t, *J* = 6.3 Hz, 1H, Ar-H), 7.41–7.37 (m, 1H, Ar-H), 7.15 (d, *J* = 9.0 Hz, 2H, Ar-H), 7.04 (dd, *J* = 1.9 Hz, 1H, Ar-H), 6.83 (d, *J* = 1.9 Hz, 1H, Ar-H), 5.28 (s, 2H, CH_2_).

*E*-diastereomer: 10.74 (s, 1H, NH), 8.59 (t, *J* = 6.3 Hz, 1H, Ar-H), 8.45 (d, *J* = 8.9 Hz, 2H, Ar-H), 7.84–7.79 (m, 2H, Ar-H), 7.71 (t, *J* = 7.7 Hz, 1H, Ar-H), 7.59 (s, 1H, C=CH), 7.36–7.33 (m, 1H, Ar-H), 7.13 (d, *J* = 9.0 Hz, 2H, Ar-H), 7.02 (dd, *J* = 1.9 Hz, 1H, Ar-H), 6.82 (d, *J* = 1.9 Hz, 1H, Ar-H), 5.28 (s, 2H, CH_2_).

^13^C NMR (101 MHz, DMSO-*d*_6_) δ:

*Z*-diastereomer: 167.69, 160.09, 149.66, 144.45, 138.29, 137.55, 137.27, 135.05, 134.11, 132.15, 127.64, 127.23, 125.08, 123.58, 123.52, 122.33, 115.64, 115.09, 70.96.

*E*-diastereomer: 162.08, 160.09, 149.66, 144.45, 138.29, 137.52, 137.27, 135.05, 132.75, 123.80, 123.52, 121.29, 121.15, 120.53, 115.09, 115.41, 110.44, 109.62, 70.89.

Anal. Calcd. For C_21_H1_5_ClN_2_O_2_ (362.81) C, 69.52; H, 4.17; N, 7.72. found: C, 69.23; H, 4.09; N, 7.53.

**(*E*/*Z*)-3-[4-(Pyridin-3-ylmethoxy)benzylidene]indolin-2-one (5i)**

Yellow powder; 66% yield (57.5 mg); mp: 163–161 °C, R*_f_* = 0.32 (pet. ether:EtOAc, 1:1). ^1^H NMR (400 MHz, DMSO-*d*_6_) δ: *E*:*Z* ratio = 44:56

*Z*-diastereomer: 10.59 (s, 1H, NH), 8.58 (m, 3H, Ar-H), 7.92 (d, *J* = 7.8 Hz, 1H, Ar-H), 7.75–7.71 (m, 3H, Ar-H), 7.58 (s, 1H, C=CH), 7.28–7.18 (m, 3H, Ar-H), 7.02–6.82 (m, 2H, Ar-H), 5.25 (s, 2H, CH_2_). 

*E*-diastereomer: 10.56 (s, 1H, NH), 8.56 (m, 3H, Ar-H), 7.90 (d, *J* = 7.8 Hz, 1H, Ar-H), 7.68–7.57 (m, 3H, Ar-H), 7.44–7.41 (m, 3H, Ar-H), 7.13 (s, 1H, C=CH), 6.81–6.73 (m, 2H, Ar-H), 5.25 (s, 2H, CH_2_).

^13^C NMR (100 MHz, DMSO*-d*_6_) δ:

*Z*-diastereomer: 168.89, 159.39, 149.31, 149.21, 142.71, 135.87, 135.85, 134.39, 132.29, 131.51, 129.76, 127.07, 125.85, 123.66, 122.09, 121.13, 121.05, 115.08, 114.56, 110.07, 67.16.

*E*-diastereomer: 167.38, 165.80, 159.98, 159.39, 140.30, 136.67, 128.30, 127.38, 125.31, 124.31, 120.92, 119.29, 116.77, 109.52, 109.22, 67.10, 64.79, 59.76, 54.90, 48.60, 14.08.

Anal. Calcd. For C_21_H_16_N_2_O_2_ (328.37) C, 76.81; H, 4.91; N, 8.53. found: C, 76.53; H, 5.21; N, 8.24.

**(*E*/*Z*)-5-Fluoro-3-[4-(pyridin-3-ylmethoxy)benzylidene]indolin-2-one (5j)**

Yellow powder; 75% yield (133 mg); mp: 187–189 °C, R*_f_* = 0.42 (pet. ether:EtOAc, 1:1). ^1^H NMR (400 MHz, DMSO-*d*_6_) δ: *E*:*Z* ratio = 38:62

*Z*-diastereomer: 10.59 (s, 1H, NH), 8.98–8.67 (m, 2H, Ar-H), 8.16–7.66 (m, 3H, Ar-H), 7.57 (m, 2H, Ar-H), 7.45 (s,1H, C=CH), 7.13 (m, 3H, Ar-H), 6.85 (d, *J* = 8.9 Hz, 1H), 5.26 (s, 2H, CH_2_).

*E*-diastereomer: 10.59 (s, 1H, NH), 8.66–8.28 (m, 2H, Ar-H), 8.14–7.66 (m, 3H, Ar-H), 7.55 (m, 2H, Ar-H 7.40 (m, 2H, Ar-H), 7.12 (m, 1H, Ar-H), 7.00 (s, 1H, C=CH), 6.83 (d, *J* = 8.9 Hz, 1H), 5.26 (s, 2H, CH2).

^13^C NMR (100 MHz, DMSO*-d*_6_) δ:

*Z*-diastereomer: 168.89, 167.44, 160.34, 159.71, 149.33, 149.23, 139.04, 138.44, 137.62, 136.50, 135.89, 134.72, 131.64, 127.18, 123.96, 123.70, 116.13, 115.22, 114.67, 106.89, 67.21.

*E*-diastereomer: 156.03, 136.49, 132.25, 126.67, 123.70, 122.17, 115.89,110.77, 109.89, 109.23, 108.98, 67.15, 54.91, 40.15, 39.93, 39.73, 39.51, 39.31, 39.10, 38.89, 14.10.

Anal. Calcd. For C_21_H_15_FN_2_O_2_ (346.36) C, 72.82; H, 4.37; N, 8.09. found: C, 72.51; H, 4.18; N, 8.24.

**(*E*/*Z*)-5-Chloro-3-[4-(pyridin-3-ylmethoxy)benzylidene] indolin-2-one (5k)**

Yellow powder; 84% yield (120 mg); mp: 199–201 °C, R*_f_* = 0.43 (pet. ether:EtOAc, 1:1). ^1^H NMR (400 MHz, DMSO-*d*_6_) δ: *E*:*Z* ratio = 41:59

*Z*-diastereomer: 10.71 (s, 1H, NH), 8.72 (d, *J* = 9.0 Hz, 1H, Ar-H), 8.64 (d, *J* = 8.2 Hz, 2H, Ar-H), 8.54 (m, 1H, Ar-H), 7.93–7.89 (m, 2H, Ar-H), 7.67 (s, 1H, C=CH), 7.55 (d, *J* = 1.9 Hz, 1H, Ar-H), 7.37–7.20 (m, 3H, Ar-H), 6.85 (d, *J* = 8.3 Hz, 1H, Ar-H), 5.27 (s, 2H, CH_2_).

*E*-diastereomer: 10.71 (s, 1H, NH), 8.70 (d, *J* = 9.0 Hz, 1H, Ar-H), 8.55–8.47 (s, 1H, Ar-H), 7.84–7.81 (d, *J* = 8.2 Hz, 2H, Ar-H), 7.70–7.63 (m, 2H, Ar-H), 7.67 (m, 3H, Ar-H), 7.54 (d, *J* = 1.9 Hz, 1H, Ar-H), 7.29 (s, 1H, C=CH), 6.84 (d, *J* = 8.3 Hz, 1H, Ar-H), 5.24 (s, 2H, CH_2_).

^13^C NMR (100 MHz, DMSO*-d*_6_) δ:

*Z*-diastereomer: 160.38, 149.31, 149.20, 138.89, 138.67, 135.86, 135.83, 134.76, 132.21, 131.60, 129.22, 127.19, 126.62, 125.29, 124.88, 123.63, 123.15, 115.17, 114.66, 110.54, 108.84, 67.12.

*E*-diastereomer: 161.97, 160.38, 159.72, 149.31, 149.20, 141.44, 138.89, 138.67, 137.81, 131.17, 128.79, 127.61, 121.44, 119.33, 114.22, 111.39, 67.18, 64.26, 59.74, 54.89, 45.93, 20.74.

Anal. Calcd. For C_21_H_15_ClN_2_O_2_ (362.81) C, 69.52; H, 4.17; N, 7.72. found: C, 69.33; H, 4.02; N, 7.84. 

**(*E*/*Z*)-6-Chloro-3-[4-(pyridin-3-ylmethoxy) benzylidene] indolin-2-one (5l)**

Yellow powder; 79% yield (125 mg); mp: 216–218 °C, R*_f_* = 0.38 (pet. ether:EtOAc, 1:1). ^1^H NMR (400 MHz, DMSO-*d*_6_) δ: *E*:*Z* ratio = 39:61

*Z*-diastereomer: 10.74 (s, 1H, NH), 8.71 (s, 1H, Ar-H), 8.57–8.55 (t, *J* = 5.9 Hz, 2H, Ar-H), 7.91 (d, *J* = 7.7 Hz, 1H, Ar-H), 7.80 (s,1H, C=CH), 7.50 (m, 2H, Ar-H), 7.46 (t, *J* = 7.6 Hz, 1H, Ar-H), 7.17 (t, *J* = 8.7 Hz, 2H, Ar-H), 7.08–6.79 (m, 2H, Ar-H), 5.26 (s, 2H, CH_2_).

*E*-diastereomer: δ 10.74 (s, 1H, NH), 8.71 (s, 1H, Ar-H), 8.49–8.46 (t, *J* = 5.9 Hz, 2H, Ar-H), 7.90 (d, *J* = 7.7 Hz, 1H, Ar-H), 7.63 (s,1H, C=CH), 7.46 (m, 2H, Ar-H), 7.44 (t, *J* = 7.6 Hz, 1H, Ar-H), 7.20 (t, *J* = 8.7 Hz, 2H, Ar-H), 7.08–6.92 (m, 2H, Ar-H), 5.26 (s, 2H, CH_2_).

^13^C NMR (100 MHz, DMSO*-d*_6_) δ:

*Z*-diastereomer: 168.83, 159.58, 149.31, 149.20, δ 144.00, 136.78, 135.83, 134.57, 133.66, 132.23,131.65, 126.80, 124.65, 123.65, 120.79, 120.70, 120.71, 115.15, 114.62,67.18, 67.12.

*E*-diastereomer: 167.23, 167.55, 161.89, 160.23, 160.06, 141.36, 137.79, 132.90, 132.30, 127.21, 124.29, 123.27, 123.08, 120.07, 109.98, 109.16, 64.41, 59.74, 54.89, 46.30, 14.07.

Anal. Calcd. For C_21_H_15_ClN_2_O_2_ (362.81) C, 69.52; H, 4.17; N, 7.72. found: C, 69.23; H, 4.37; N, 7.64.

**(*E*/*Z*)-3-[4-(Pyridin-4-ylmethoxy) benzylidene] indolin-2-one (5m)**

Yellow powder; 87% yield (160 mg); mp: 238–240 °C, R*_f_* = 0.33 (pet. ether:EtOAc, 1:1). ^1^H NMR (400 MHz, DMSO-*d*_6_) δ: *E*:*Z* ratio = 37:63

*Z*-diastereomer: 10.56 (s, 1H, NH), 8.61 (d, *J* = 4.9 Hz, 2H, Ar-H), 7.73 (d, *J* = 5.1 Hz, 2H, Ar-H), 7.68–7.59 (m, 3H, Ar-H), 7.58 (s, 1H, C=CH), 7.31–7.20 (m, 3H, Ar-H), 6.88 (t, *J* = 7.3 Hz, 2H), 5.29 (s, 2H, CH_2_).

*E*-diastereomer: 10.56 (s, 1H, NH), 8.59 (d, *J* = 4.9 Hz, 2H, Ar-H), 7.71 (m, 3H, Ar-H), 7.58–7.55 (m, 3H, Ar-H), 7.47 (d, *J* = 5.1 Hz, 2H, Ar-H), 7.20 (s, 1H, C=CH), 6.86 (t, *J* = 7.3 Hz, 2H), 5.29 (s, 2H, CH_2_).

^13^C NMR (100 MHz, DMSO*-d*_6_) δ:

*Z*-diastereomer: 169.34, 159.63, 150.26, 146.28, 143.19, 137.07, 136.28, 134.85, 132.00, 130.25, 127.67, 126.38, 122.57, 122.35, 121.58, 121.52, 121.39, 115.56, 115.03, 110.54, 68.17.

*E*-diastereomer: 166.27, 160.21, 150.26, 145.87, 136.73,136.02, 134.85, 133.96,131.17, 129.90, 128.81, 127.96, 126.68, 122.35, 120.04, 119.78, 118.42, 112.21, 109.69, 68.17, 51.50.

Anal. Calcd. For C_21_H_16_N_2_O_2_ (328.37) C, 76.81; H, 4.91; N, 8.53. found C, 77.19; H, 4.72; N, 8.38.

**(*E*/*Z*)-5-Fluoro-3-[4-(pyridin-4-ylmethoxy)benzylidene] indolin-2-one (5n)**

Yellow powder; 71% yield (116 mg); mp: 233–235 °C, R*_f_* = 0.29 (pet. ether:EtOAc, 1:1). ^1^H NMR (400 MHz, DMSO-*d_6_*) δ: *E*:*Z* ratio = 5:95

*Z*-diastereomer: 10.59 (s, 1H, NH), 8.61–8.59 (m, 2H, Ar-H), 7.73 (d, *J* = 8.6 Hz, 2H), 7.58 (s, 1H, C=CH), 7.48 (d, *J* = 6.0 Hz, 2H, Ar-H), 7.35 (t, *J* = 9.4 Hz, 1H), 7.21 (d, *J* = 8.8 Hz, 2H, Ar-H), 7.07 (t, *J* = 5.8 Hz, 1H, Ar-H), 6.85 (t, *J* = 4.7 Hz, 1H, Ar-H), 5.29 (s, 2H, CH_2_).

*E*-diastereomer: 10.59 (s, 1H, NH), 8.50–8.47 (m, 2H, Ar-H), 7.65 (s, 1H, C=CH), 7.58 (d, *J* = 8.8 Hz, 2H, Ar-H), 7.46 (d, *J* = 6.0 Hz, 2H, Ar-H), 7.33 (t, *J* = 9.4 Hz, 1H), 7.20 (d, *J* = 8.6 Hz, 2H), 7.05 (t, *J* = 5.8 Hz, 1H, Ar-H), 6.87 (t, *J* = 4.7 Hz, 1H, Ar-H), 5.29 (s, 2H, CH_2_).

^13^C NMR (100 MHz, DMSO*-d*_6_) δ:

*Z*-diastereomer: 169.32, 160.55, 159.92, 158.82, 150.25, 146.23, 138.81, 137.99, 136.97, 135.15, 132.10, 127.74, 127.25, 122.36, 116.36, 115.68, 115.12, 110.42, 109.45, 107.36, 68.19.

*E*-diastereomer:168.61, 162.23, 156.47, 143.28, 139.51, 138.39, 137.99, 135.15, 132.86,127.25, 126.29, 124.51, 123.08, 122.59, 118.52, 116.59, 111.80, 111.21, 110.10, 109.70, 26.21.

Anal. Calcd. For C_21_H_15_FN_2_O_2_ (346.36) C, 72.82; H, 4.37; N, 8.09. found: C, 72.50; H, 4.58; N, 8.26.

**(*E*/*Z*)-5-Chloro-3-[4-(pyridin-4-ylmethoxy)benzylidene] indolin-2-one (5o)**

Yellow powder; 81% yield (140 mg); mp: 238–240 °C, R*_f_* = 0.30 (pet. ether:EtOAc, 1:1). ^1^H NMR (400 MHz, DMSO-*d*_6_) δ: *E*:*Z* ratio = 57:43

*Z*-diastereomer: 10.71 (s, 1H, NH), 8.68–8.59 (m, 2H, Ar-H), 7.88 (s, 1H, C=CH), 7.66 (t, *J* = 5.3 Hz, 2H, Ar-H), 7.53 (d, *J* = 1.9 Hz, 1H, Ar-H), 7.46 (t, *J* = 6.0 Hz, 2H, Ar-H), 7.28 (t, *J* = 8.3 Hz, 1H, Ar-H), 7.17 (t, *J* = 8.8 Hz, 2H, Ar-H), 6.89 (t, *J* = 8.3 Hz, 1H, Ar-H), 5.29 (s, 2H, CH_2_).

*E*-diastereomer: 10.71 (s, 1H, NH), 8.51–8.47 (m, 2H, Ar-H), 7.66 (s, 1H, C=CH), 7.64 (t, *J* = 5.3 Hz, 2H, Ar-H), 7.48 (d, *J* = 1.9 Hz, 1H, Ar-H), 7.46 (t, *J* = 6.0 Hz, 2H, Ar-H), 7.19 (t, *J* = 8.3 Hz, 1H, Ar-H), 7.17 (t, *J* = 8.8 Hz, 2H, Ar-H), 6.85 (t, *J* = 8.3 Hz, 1H, Ar-H), 5.29 (s, 2H, CH_2_).

^13^C NMR (100 MHz, DMSO*-d_6_*) δ:

*Z*-diastereomer: 160.61, 150.25, 146.20, 139.38, 139.08, 138.23, 135.23, 132.09, 129.72, 127.78, 127.73, 127.24, 125.79, 123.73, 122.37, 122.35, 119.83, 115.66, 111.03, 68.19.

*E*-diastereomer: 159.96, 150.25,141.93, 139.08, 135.23, 129.72, 128.13, 127.73, 127.24, 125.54, 125.38, 123.73, 123.24, 122.37, 119.83, 116.45, 115.13, 111.88, 68.19, 68.13, 30.56.

Anal. Calcd. For C_21_H_15_ClN_2_O_2_ (362.81) C, 69.52; H, 4.17; N, 7.72. found: C, 69.10; H, 4.48; N, 7.57.

**(*E*/*Z*)-6-Chloro-3-[4-(pyridin-4-ylmethoxy)benzylidene]indolin-2-one (5p)**

Yellow powder; 86% yield (154 mg); mp: 233–235 °C, R*_f_* = 0.28 (pet. ether:EtOAc, 1:1). ^1^H NMR (400 MHz, DMSO-*d*_6_) δ: *E*:*Z* ratio = 17:82

*Z*-diastereomer: 10.72 (s, 1H, NH), 8.61–8.59(t, *J* = 7.0 Hz, 2H, Ar-H), 7.84 (s,1H, Ar-H), 7.67 (d, *J* = 8.3 Hz, 2H, Ar-H), 7.62 (s, 1H, C=CH), 7.47 (d, *J* = 5.1 Hz, 2H, Ar-H), 7.15 (t, *J* = 8.6 Hz, 2H, Ar-H), 7.07–6.84 (m, 2H, Ar-H), 5.29 (s, 2H, CH_2_).

*E*-diastereomer: 10.72 (s, 1H, NH), 8.47–8.45 (t, *J* = 7.0 Hz, 2H, Ar-H), 7.70 (s,1H, Ar-H), 7.63 (d, *J* = 8.3 Hz, 2H, Ar-H), 7.59 (s, 1H, C=CH), 7.46 (d, *J* = 5.1 Hz, 2H, Ar-H), 7.12 (t, *J* = 8.6 Hz, 2H, Ar-H), 6.82 (m, 2H, Ar-H), 5.29 (s, 2H, CH_2_).

^13^C NMR (100 MHz, DMSO*-d*_6_) δ:

*Z*-diastereomer: 169.30, 159.83, 150.26, 146.24, 144.48, 138.21, 137.20, 135.05, 134.17, 132.15, 127.40, 125.19, 123.78, 122.35, 121.28, 121.21, 120.52, 115.65, 115.10, 110.47, 68.19.

*E*-diastereomer: 168.53, 167.70, 162.97, 160.46, 145.54142.91, 141.85, 137.72,136.97, 133.99, 132.81, 127.79, 127.57, 126.62, 124.88, 124.74, 123.65, 122.10,112.84, 109.65, 68.19, 68.12.

Anal. Calcd. For C_21_H_15_ClN_2_O_2_ (362.81) C, 69.52; H, 4.17; N, 7.72. found: C, 69.83; H, 4.38; N, 7.53.

### 3.2. Biological Assays

#### 3.2.1. Screening of Antiproliferative Activity by NCI

A total of 16 novel oxindole derivatives were sent to the National Cancer Institute (Germantown, MD, USA) for comprehensive anticancer screening [38]. Anticancer activity was tested against 60 cancer cell lines (NCI-60) after 48h treatments with a single dosage of 10 μM of compounds by using sulforhodamine B assay [39] on the traditional 96-well platform. The NCI-60 panel covers nearly 60 cancer cell models of leukemia, non-small cell lung cancer (NSCLC), colon, central nervous system (CNS), melanoma, ovarian, renal, prostate and breast cancers. The human cancer cell lines of the NCI-60 panel are grown in RPMI 1640 medium supplemented with 5% FBS (fetal bovine serum) and 2 mM L-glutamine. The detailed cell line list, cellular doubling times, and inoculation densities can be found on https://dtp.cancer.gov/discovery_development/nci-60/cell_list.htm (accessed on 10 November 2023). For further analysis of promising compounds, five dose screens were performed against the NCI-60 panel. Drug treatments are performed in 5 doses using 10-fold or ½ log dose dilutions. The relevant protocol can be accessed in full detail at https://dtp.cancer.gov/discovery_development/nci-60/methodology.htm (accessed on 10 November 2023). 

#### 3.2.2. Kinase Screening of Compound **5l**

The inhibition percentages of the target compound **5l** at a concentration of 10 µM were assessed using DMSO as a solvent against ten kinases, namely PTK2B, JAK1, CDK2, FGFR1, IGF1R, VEGFR-2, PDGFRα, PDGFRβ, FLT3, and Src. The Z’-LYTE^®^ technology, which relies on FRET (Fluorescence Resonance Energy Transfer) from Invitrogen/Life Technologies, was employed for this evaluation for all kinases except VEGFR-2. The VEGFR-2 kinase assay was conducted in a 96-well streptavidin-coated plate using a recombinant human VEGFR-2/KDR ELISA kit, following the manufacturer’s instructions. Given the heightened activity of **5l** against CDK2 and FLT3, the IC_50_ values of **5l**, sunitinib, and FN-1501 against these two kinases were determined, with sunitinib and FN-1501 serving as the reference drugs. Statistical significance was assessed using one-way analysis of variance (ANOVA), followed by the Dunnett test; data are represented as means ± SD.

### 3.3. Molecular Modelling

#### 3.3.1. Molecular Docking

Molecular docking commenced with the importation of the target protein structures from the Protein Data Bank (PDB), specifically using PDB IDs: 3TI1 for CDK2, and 6JQR for FLT3. These structures underwent preparation in the Maestro v13.3 interface, which included the removal of water molecules, the addition of hydrogen atoms, and the assignment of appropriate charge states to ensure an accurate physiological representation. Ligand preparation involved optimizing compound **5l**’s geometry and confirming its tautomeric and protonation states, crucial for interaction fidelity. A grid representing the active site was created around the known binding pockets, using the co-crystallized ligands as a reference for CDK2 and FLT3. The Glide XP docking [40] protocol then systematically searched for the optimal conformation of compound **5l** within these grids, utilizing an empirical scoring function to evaluate each potential binding mode. Validation was performed through the re-docking of the co-crystallized ligands, ensuring that the docking protocol could reliably reproduce known binding poses. Post-docking interaction analyses were facilitated by PLIP [41], which provided detailed insights into the binding interactions such as hydrogen bonds and hydrophobic contacts. The docked conformations were visualized using the PyMOL Molecular Graphics System v2.4, offering a three-dimensional perspective on the spatial arrangement of the interactions within the active sites.

#### 3.3.2. MD Simulations

The MD simulations for the protein–ligand complexes of CDK2-**5l** and sunitinib, and of FLT3-**5l** and sunitinib were conducted using Gromacs 2021.2 [42], with the system setup prepared on the CHARMM-GUI [43,44] server using the CHARMM36m [45] force fields. The van der Waals interactions were managed using a cutoff type with a force-switch modifier. Here, a switching distance was set at 1.0 nm where the force-switching function begins, and the cutoff distance was set at 1.2 nm where the van der Waals interaction effectively becomes zero. This setup ensures a smooth transition in the computation of van der Waals forces, minimizing potential artifacts in the simulation. For electrostatic interactions, the Particle Mesh Ewald (PME) method was employed with a cutoff distance of 1.2 nm, ensuring accurate computation of long-range electrostatic forces. Temperature regulation was achieved via the Nose–Hoover thermostat, with separate temperature coupling groups for the solute and solvent at a target of 303.15 K. Pressure was maintained using the Parrinello–Rahman barostat with isotropic coupling, a compressibility setting of 4.5 × 10^−5^ bar^−1^, and a reference pressure of 1.0 atm. Constraints on hydrogen bonds were applied using the LINCS algorithm, facilitating a more stable and efficient simulation. The communication between solute and solvent was managed linearly with updates every 100 steps to maintain the center of mass. The ensuing 100 ns MD simulations captured the temporal evolution of the compound’s interaction within the active sites. For post-simulation analysis, the MM/PBSA method [46] was employed to calculate binding free energies, offering insights into the affinity of compound **5l** with the kinase targets. MM/PBSA calculations were performed on 200 frames extracted between 80 and 100 ns, providing a comprehensive view of the binding energetics. Graphical representations of RMSD, PCA, and hydrogen bond dynamics were generated using QtGrace v0.2.6. Additionally, animations showcasing the dynamic molecular interactions within the MD simulations were created with PyMOL Molecular Graphics System v2.4.

#### 3.3.3. Prediction of Physicochemical and Pharmacokinetic Properties

To predict the ADME parameters, pharmacokinetic properties, and the drug-likeness of the novel compound **5l**, the SwissADME online tool by the SIB was utilized [47]. The molecular structures of the compounds were encoded into SMILES notations and inputted into the server. Toxicity profiles were ascertained using the pkCSM [48] pharmacokinetics website, facilitating a comprehensive assessment of the safety and efficacy potential of compound **5l**. This predictive analysis forms the cornerstone of a prudent drug development strategy, allowing for the identification of favorable physicochemical characteristics and a manageable safety profile early in the drug development process.

## 4. Conclusions

A new series of oxindole-based derivatives linked to various pyridyl groups were synthesized and evaluated for their antiproliferative potential against the NCI 60 cancer cell panel. A subpanel of leukemia was selectively inhibited by compound **5l**, which has nanomolar IC_50_ values for FLT3 (IC_50_ = 36.21 ± 1.07 nM), and CDK2 (IC_50_ = 8.17 ± 0.32 nM). A structure–activity–relationship investigation demonstrated that in order to inhibit FLT3 and CDK simultaneously, a 3-pyridyl group should be added to the linker’s 4-hydroxy group in addition to a chloro group at the 6-position of the oxindole ring. Molecular docking and dynamics have shown that compound **5l** has a strong binding affinity and is very stable in the FLT3 and CDK2 binding pockets. These results point to the possibility of developing this novel scaffold into a medication for leukemia.

## Figures and Tables

**Table 1:** *E*:*Z* diastereomeric ratios of the synthesized compounds (**5a–p**).

Compound	Substituents		Chemical Shifts (Vinyl H, δ ppm)		Chemical Shifts (2′ and 6′-Hs, δ ppm)		*E*:*Z* Ratio ^a^
	R	Ar	*E*	*Z*	*E*	*Z*	
**5a**	H	phenyl	7.34	7.75	7.36	7.68	3:97
**5b**	5-F	phenyl	7.33	7.82	7.35	7.61	15:85
**5c**	5-Cl	phenyl	7.33	7.89	7.35	7.81	35:65
**5d**	6-Cl	phenyl	7.30	7.80	7.36	7.69	15:85
**5e**	H	2-pyridyl	7.22	7.36	7.58	7.73	31:69
**5f**	5-F	2-pyridyl	7.38	7.65	7.60	7.73	33:67
**5g**	5-Cl	2-pyridyl	7.57	7.66	7.29	7.72	23:77
**5h**	6-Cl	2-pyridyl	7.59	7.81	7.38	7.71	47:53
**5i**	H	3-pyridyl	7.13	7.58	7.63	7.72	44:56
**5j**	5-F	3-pyridyl	7.00	7.45	7.36	8.57	38:62
**5k**	5-Cl	3-pyridyl	7.29	7.67	7.19	7.76	41:59
**5l**	6-Cl	3-pyridyl	7.63	7.80	7.62	8.47	39:61
**5m**	H	4-pyridyl	7.20	7.58	7.64	7.24	37:63
**5n**	5-F	4-pyridyl	7.65	7.58	7.00	7.73	5:95
**5o**	5-Cl	4-pyridyl	7.66	7.88	7.73	7.80	57:43
**5p**	6-Cl	phenyl	7.59	7.62	7.04	7.73	17:82

^a^ *E*:*Z* ratio was calculated according to the ratio between the relative integration of vinylic H for *E* and *Z* isomers in ^1^H NMR.

**Table 2:** Growth inhibition (%) induced by 10 µM of compounds **5a–p** against NCI-60 cell lines panel (9 different cancer types).

		5a	5b	5c	5d	5e	5f	5g	5h	5i	5j	5k	5l	5m	5n	5o	5p
**Leukemia**	**CCRF-CEM**	0.52	−10.84	0.21	−2.18	57.72	5.79	16.80	53.72	52.44	75.00	36.38	83.93	40.51	40.63	53.85	34.36
	**HL-60(TB)**	11.51	7.92	−3.22	−13.14	38.75	13.93	14.99	5.33	49.87	81.68	14.82	95.54	55.26	57.75	66.51	52.44
	**K-562**	11.80	7.19	−0.34	6.91	59.21	14.63	48.95	31.35	75.91	86.13	38.76	95.05	62.67	79.32	72.45	59.07
	**MOLT-4**	2.73	−0.05	3.28	0.86	62.52	3.55	53.15	61.47	69.98	88.53	52.62	98.08	78.47	76.73	81.22	79.00
	**RPMI-8226**	1.13	2.59	−3.85	−7.36	63.99	19.38	26.10	32.41	45.37	61.23	29.67	95.51	71.47	64.51	70.50	74.88
	**SR**	14.63	−0.14	−0.06	−4.93	57.72	ND	45.10	42.78	72.04	72.99	66.11	84.20	73.29	85.44	88.88	72.56
**Non-Small Cell Lung Cancer**	**A549/ATCC**	−3.26	3.90	−0.99	−4.59	29.20	−1.04	35.64	62.53	26.18	39.36	16.13	58.80	34.51	29.79	39.94	27.89
	**EKVX**	0.69	0.65	0.85	−0.31	47.31	15.77	34.26	49.37	31.70	41.04	16.06	60.93	63.53	52.16	37.61	45.07
	**HOP-62**	−4.06	−1.20	3.77	3.85	25.62	−7.71	54.44	18.92	37.24	45.44	0.56	72.90	23.04	17.04	19.68	21.38
	**HOP-92**	−7.00	4.77	9.75	−8.67	68.98	32.93	27.95	35.77	35.39	25.82	−1.40	56.06	40.19	36.92	16.30	49.65
	**NCI-H226**	4.45	9.53	11.09	7.13	40.80	6.67	98.74	48.64	38.16	55.56	11.80	62.93	42.40	44.48	38.30	49.48
	**NCI-H23**	−2.42	1.73	−1.96	5.71	21.37	28.01	37.77	47.43	42.43	58.20	16.74	75.60	49.83	58.56	55.31	57.17
	**NCI-H322M**	0.44	2.81	−0.07	0.49	29.48	3.29	35.75	24.71	ND	ND	ND	ND	20.85	27.56	40.64	22.52
	**NCI-H460**	−8.64	−0.94	−3.66	−4.48	14.97	11.60	84.01	56.72	21.71	53.94	14.33	77.73	40.54	44.40	57.75	36.76
	**NCI-H522**	2.18	5.91	2.10	0.19	24.56	68.08	33.95	31.58	49.90	89.53	19.75	103.96	32.70	81.98	86.03	45.21
**Colon Cancer**	**COLO 205**	−13.05	1.92	−6.62	−9.74	−14.73	−10.80	10.90	−3.47	26.13	46.06	5.66	96.36	38.26	22.07	19.21	39.71
	**HCC-2998**	−13.07	−9.12	−3.96	−1.79	1.60	−20.25	−4.51	16.74	−2.08	23.37	−9.21	50.88	20.64	25.74	12.39	20.13
	**HCT-116**	4.24	4.15	3.41	2.33	41.10	57.83	71.55	47.82	56.52	84.67	34.64	92.99	40.51	56.12	60.51	61.51
	**HCT-15**	0.04	3.51	−4.25	5.00	45.93	10.70	64.27	45.50	60.55	84.77	46.54	97.24	62.31	78.75	79.14	76.92
	**HT29**	−6.64	2.38	−2.95	−5.27	16.23	−0.51	32.53	19.66	32.66	84.09	18.10	90.33	11.25	36.44	39.69	37.78
	**KM12**	−3.15	2.42	−1.02	4.92	61.27	18.82	21.25	21.65	87.44	97.33	52.80	99.70	66.27	69.85	60.64	47.83
	**SW-620**	−7.33	−2.34	1.08	0.60	ND	−2.79	52.53	ND	19.95	70.10	2.62	92.73	21.97	41.10	48.33	49.50
**CNS Cancer**	**SF-268**	−1.74	5.43	3.26	5.55	27.33	29.87	20.68	23.17	−19.59	11.10	5.10	45.36	22.71	38.83	55.71	16.72
	**SF-295**	−6.78	1.11	−11.14	−14.66	27.03	1.98	15.52	14.28	31.20	54.81	4.51	63.31	58.27	51.70	44.17	32.18
	**SF-539**	−3.40	4.67	4.12	5.48	35.34	47.67	26.50	57.68	36.20	63.28	20.23	59.26	24.88	41.74	43.95	94.23
	**SNB-19**	2.17	1.26	−1.05	−3.09	39.30	12.95	49.26	19.99	37.12	62.06	22.92	56.18	43.13	60.46	73.78	28.72
	**SNB-75**	14.60	5.15	13.07	26.70	34.73	10.24	−12.56	63.32	−1.11	−5.67	−10.24	18.28	35.24	26.57	21.24	14.90
	**U251**	−3.49	0.40	−1.54	−4.25	46.45	41.77	76.30	61.58	53.17	83.65	27.33	76.38	38.92	61.27	66.89	64.39
**Melanoma**	**LOX IMVI**	−7.76	−6.24	0.14	7.32	ND	12.60	43.51	ND	54.99	76.91	10.09	91.00	41.37	67.26	58.74	65.24
	**MALME-3M**	−7.59	7.33	−12.99	−15.38	25.40	7.24	61.53	18.04	ND	ND	ND	ND	17.41	26.79	43.03	12.53
	**M14**	5.67	3.39	7.34	3.83	8.30	−5.09	10.07	20.00	37.62	67.06	12.17	77.08	23.70	32.49	33.05	16.86
	**MDA-MB-435**	−2.33	5.59	−3.79	0.59	10.09	−2.77	23.10	23.86	60.93	98.28	29.29	121.06	49.62	61.67	58.73	29.23
	**SK-MEL-2**	−4.59	ND	−0.35	−11.32	−12.23	−3.45	−11.36	−10.28	24.10	50.38	0.36	66.23	27.19	28.05	4.23	27.64
	**SK-MEL-28**	−7.21	2.92	−8.75	−2.97	−0.76	−8.53	−0.48	−8.02	−2.65	28.18	−20.47	46.27	−4.73	−9.39	14.48	−10.92
	**SK-MEL-5**	1.96	−0.59	1.90	2.83	48.36	−5.85	10.49	23.98	59.40	112.65	11.41	152.58	90.02	93.11	62.56	64.97
	**UACC-257**	−9.45	2.53	−6.58	−16.07	12.60	−4.06	−18.19	−2.10	30.52	39.79	2.42	76.69	35.79	23.43	23.69	17.22
	**UACC-62**	6.11	4.38	5.69	4.09	40.38	9.94	33.28	49.62	43.48	57.78	23.29	64.31	31.09	28.35	37.60	34.70
**Ovarian Cancer**	**IGROV1**	−9.53	2.92	−5.14	8.41	36.84	6.35	45.40	26.16	ND	ND	ND	ND	25.35	64.65	84.73	67.88
	**OVCAR-3**	ND	ND	ND	ND	18.16	8.17	42.68	6.16	−9.24	9.05	−10.93	61.78	21.49	39.24	67.36	53.00
	**OVCAR-4**	−7.99	0.82	−7.18	3.65	30.83	5.38	101.81	75.83	37.83	47.23	21.23	68.41	42.67	43.43	47.34	31.80
	**OVCAR-5**	−2.24	−0.99	−2.92	−0.55	5.53	−9.45	11.76	15.63	−27.58	−11.18	−34.52	21.73	−33.20	−27.63	−14.36	-29.38
	**OVCAR-8**	2.34	−0.84	−0.47	−0.41	29.68	3.52	54.06	13.60	47.19	65.13	23.45	88.63	35.02	56.25	61.47	61.29
	**NCI/ADR-RES**	−0.55	0.01	1.88	−4.24	46.72	21.70	85.88	38.98	54.97	85.57	26.04	112.40	61.81	74.16	67.62	59.29
	**SK-OV-3**	−5.78	0.16	−1.70	−3.01	16.83	3.75	10.80	4.39	13.15	13.15	5.36	32.24	9.38	13.69	11.41	15.54
**Renal Cancer**	**786-0**	3.67	0.43	−2.86	−4.97	17.97	16.63	74.30	18.66	17.25	24.20	1.47	47.25	14.60	19.37	32.37	23.86
	**A498**	7.90	ND	−2.37	6.98	16.42	32.51	54.92	−11.54	−17.75	−29.11	−31.56	8.90	2.17	12.87	1.01	−1.39
	**ACHN**	−4.27	−7.30	−1.45	5.18	33.62	23.75	43.04	9.61	37.54	51.84	16.38	55.13	24.10	70.27	70.23	34.02
	**CAKI-1**	2.00	8.23	13.42	10.70	40.75	33.47	38.44	28.79	56.73	67.60	51.03	69.80	45.63	62.32	38.71	52.78
	**RXF 393**	−16.17	0.31	−5.38	−14.25	44.78	24.20	ND	38.18	24.86	37.26	6.84	63.42	43.88	47.62	73.84	67.40
	**SN12C**	6.16	0.27	0.70	4.03	47.67	4.04	55.48	24.90	27.14	46.15	9.17	66.06	21.40	41.32	34.41	34.65
	**TK-10**	−8.25	4.82	−12.74	−53.98	12.67	4.47	9.89	−0.24	−15.67	−1.92	−37.80	29.45	17.94	29.95	17.72	−0.51
	**UO-31**	14.90	2.55	23.92	23.83	61.16	26.54	40.45	38.05	ND	ND	ND	ND	44.72	54.75	51.15	48.99
**Prostate Cancer**	**PC-3**	5.85	−2.77	12.86	11.75	44.33	8.70	37.93	54.69	29.76	43.60	24.30	67.11	45.96	47.39	52.41	44.98
	**DU-145**	−4.38	0.40	−5.61	−1.25	20.29	7.73	12.18	39.56	39.82	46.09	0.75	58.61	33.71	38.44	49.28	29.35
**Breast Cancer**	**MCF7**	15.76	7.57	16.00	38.42	50.78	37.75	71.08	63.66	57.95	91.04	55.46	90.45	71.59	91.77	92.81	71.58
	**MDA-MB-231**	−10.01	2.43	−5.30	−3.65	ND	14.92	78.27	ND	35.36	42.04	9.08	67.52	16.63	20.73	24.93	32.73
	**HS 578T**	−6.13	−0.32	−2.25	3.27	ND	21.88	85.17	ND	17.58	37.36	3.67	62.66	17.94	28.85	27.03	34.99
	**BT-549**	−5.74	−6.03	−2.68	−1.23	41.23	13.15	21.20	33.81	53.20	65.71	14.50	95.71	ND	ND	ND	ND
	**T-47D**	0.85	3.79	4.11	4.32	54.90	60.74	31.01	37.95	51.02	66.72	23.23	68.95	65.12	64.35	61.00	67.13
	**MDA-MB-468**	0.75	1.56	−0.96	1.52	61.73	28.22	49.57	60.78	51.24	58.51	12.49	85.71	91.12	61.82	44.85	65.63

Cells shaded **gray** for non-determined activity; cells shaded **orange** for growth inhibition >60%; cells shaded **pink** for growth inhibition >90%.

**Table 3:** An overview of 10 µM single dose screening NCI-60 anticancer assay.

Compound	Mean Growth Percent	Range of Growth Inhibition on All Cells	The Most Sensitive Cell Line	Positive Cytostatic Effect ^b^	Positive Cytotoxic Effect ^c^	Sensitive/Total Cell Lines
**5a**	−1.00	−16.17 to 15.76	-	0/59	0/59	0/59
**5b**	1.62	−10.84 to 9.53	-	0/57	0/57	0/57
**5c**	0.10	−12.99 to 23.92	-	0/59	0/59	0/59
**5d**	0.13	−53.98 to 38.42	-	0/59	0/59	0/59
**5e**	33.37	−14.73 ^a^ to 68.98	HOP-92	11/56	0/56	11/56
**5f**	13.74	−20.25 ^a^ to 68.87	NCI-H522	3/59	0/59	3/59
**5g**	38.63	−18.19 ^a^ to 101.81	OVCAR-4	17/59	1/59	18/59
**5h**	30.77	−11.54 ^a^ to 75.83	OVCAR-4	11/56	0/56	11/56
**5i**	34.95	−27.57 ^a^ to 87.44	KM12	17/56	0/56	17/56
**5j**	53.95	−29.10 ^a^ to 112.65	SK-MEL-5	32/56	1/56	33/56
**5k**	14.49	−37.79 ^a^ to 66.11	SR	5/56	0/56	5/56
**5l**	72.81	8.89 to 152.57	SK-MEL-5	44/56	4/56	48/56
**5m**	38.04	−33.20 ^a^ to 91.11	MDA-MB-468	14/59	0/59	14/59
**5n**	46.02	−27.62 ^a^ to 93.11	SK-MEL-5	26/59	0/59	26/59
**5o**	47.19	−14.36 ^a^ to 92.81	MCF7	28/59	0/59	28/59
**5p**	41.27	−29.37 ^a^ to 94.23	SF-539	21/59	0/59	21/59

[a] Negative (−) indicates the cell killed. [b] The ratio between number of cell lines with percent growth from 0 to 50 and total number of cell lines. [c] The ratio between number of cell lines with percent growth of <0 and total number of cell lines.

**Table 4:** NCI in vitro testing results of GI_50_, TGI and LC_50_ of compound **5l**.

Subpanel/Tumor Cell Lines	Compound		
	5l		
	GI_50_ (µM)	TGI (µM)	LC_50_ (µM)
**Leukemia**			
CCRF-CEM	3.72	>100	>100
HL60(TB)	4.08	>100	>100
K-562	3.08	>100	>100
MOLT-4	2.99	ND	>100
RPMI-8226	3.09	>100	>100
**Non-small cell lung cancer**			
A549/ATCC	15.50	>100	>100
EKVX	6.02	>100	>100
HOP-62	12.9	>100	>100
HOP-92	6.51	62.70	>100
NCI-H226	9.00	70.00	>100
NCI-H23	5.93	>100	>100
NCI-H322M	23.20	>100	>100
NCI-H460	14.00	>100	>100
NCI-H522	12.30	82.20	>100
**Colon cancer**			
COLO 205	9.62	>100	>100
HCC-2998	13.70	>100	>100
HCT-116	6.97	>100	>100
HCT-15	3.46	25.20	>100
HT29	6.45	37.80	>100
KM12	5.30	>100	>100
SW-620	2.76	>100	>100
**CNS cancer**			
SF-268	10.80	88.10	>100
SF-295	10.60	39.90	>100
SF-539	13.60	>100	>100
SNB-19	12.40	>100	>100
U251	7.55	39.40	>100
**Melanoma**			
LOX IMVI	4.70	24.80	96.60
MELME-3M	18.90	75.70	>100
M14	29.00	>100	>100
MDA-MB-435	4.44	76.90	>100
SK-MEL-2	9.67	65.90	>100
SK-MEL-28	12.30	28.50	65.60
SK-MEL-5	3.46	14..20	71.50
UACC-257	8.06	43.80	>100
**UACC-62**	7.70	43.50	>100
Ovarian cancer			
IGROV1	10.70	3.32	>100
OVCAR-3	57.70	2.26	>100
OVCAR-5	25.00	>100	>100
OVCAR-8	7.96	45.20	>100
NCI/ADR-RES	3.45	23.60	>100
SK-OV-3	71.80	>100	>100
**Renal cancer**			
786-0	26.60	>100	>100
A498	18.00	85.20	>100
ACHN	10.80	75.30	>100
CAKI-1	4.44	26.90	>100
RXF393	9.07	90.20	>100
SN12C	9.74	66.60	>100
TK-10	77.10	>100	>100
UO-31	2.70	>100	>100
**Prostate cancer**			
PC-3	6.37	>100	>100
DU-145	13.20	54.90	>100
**Breast cancer**			
MCF7	2.90	30.90	>100
MDA-MB-231/ATCC	15.80	>100	>100
HS 578T	12.80	98.10	>100
BT-549	10.40	46.30	>100
T-47D	3.18	>100	>100
MDA-MB-468	3.76	43.00	>100

**Table 5:** Median growth inhibitory concentrations ^a^ (GI_50_, µM) of in vitro subpanel tumor cell lines for compound **5l**.

Subpanel Tumor Cell Line	5l	
	MG-MID ^a^	Selectivity Index
Leukemia	3.39	3.61
NSCL cancer	11.70	1.05
Colon cancer	5.97	2.05
CNS cancer	10.99	1.11
Melanoma	10.91	1.12
Ovarian cancer	29.43	0.42
Renal cancer	19.80	0.62
Prostate cancer	9.78	1.25
Breast cancer	8.14	1.50
Full panel MG-MID	12.23	

## Data Availability

Dataset available on request from the authors.

## Supplementary Materials

The following supporting information can be downloaded at: https://www.mdpi.com/article/10.3390/ph17050659/s1, Figure S1: RMSD profiles for compound **5l** and sunitinib for each target CDK2 and FLT3 complexes, indicating deviations from initial conformations.; Figures S2–S34: ^1^H and ^13^C NMR spectrums of compounds **5a–p**; Video S1: MD Simulation animation of 200 snapshots between 0 and 100 ns of CDK2 with compound **5l** (PDB ID: 3TI1); Video S2: MD Simulation animation of 200 snapshots between 0 and 100 ns of CDK2 with sunitinib (PDB ID: 3TI1); Video S3: MD Simulation animation of 200 snapshots between 0 and 100 ns of FLT3 with compound **5l** (PDB ID: 6JQR); Video S4: MD Simulation animation of 200 snapshots between 0 and 100 ns of FLT3 with sunitinib (PDB ID: 6JQR).
