# Supplementary material for: Discovery and Anticancer Screening of Novel Oxindole-Based Derivative Bearing Pyridyl Group as Potent and Selective Dual FLT3/CDK2 Kinase Inhibitor
Source: Pharmaceuticals (Basel). 2024 May 20;17(5):659. doi: 10.3390/ph17050659 (PMC11124822; doi:10.3390/ph17050659)
Supplement: Supplementary file 1 [file pharmaceuticals-17-00659-s001.zip › Supplementary Files/Supporting_Information_Figures_S1_S34.docx]

**Supporting information**

**Discovery and anticancer screening of novel oxindole-based derivative bearing pyridyl group as potent and selective dual FLT3/CDK2 kinase inhibitor**

Aya Soudi ^1^, Onur Bender ^2,^*, Ismail Celik ^3,4^, Amer Ali Abd El-Hafeez ^5^, Rumeysa Dogan ^2^, Arzu Atalay ^2^, Eslam B. Elkaeed ^6^, Aisha A. Alsfouk ^7^, El-Shimaa M. N. Abdelhafez ^1^, Omar M. Aly ^8^, Wolfgang Sippl ^4^, Taha F. S. Ali ^1,^*

*Correspondence: [taha.ali@mu.edu.eg](mailto:taha.ali@mu.edu.eg) (Taha F.S. Ali), [onur.bender@ankara.edu.tr](mailto:onur.bender@ankara.edu.tr) (Onur Bender)

**Contents:**

- **Figure S1:** RMSD profiles for compound **5l** and sunitinib for each target CDK2 and FLT3 complexes, indicating deviations from initial conformations.
- **Figure S2-34:** ^1^H and ^13^C NMR spectrums of compounds **5a-p**


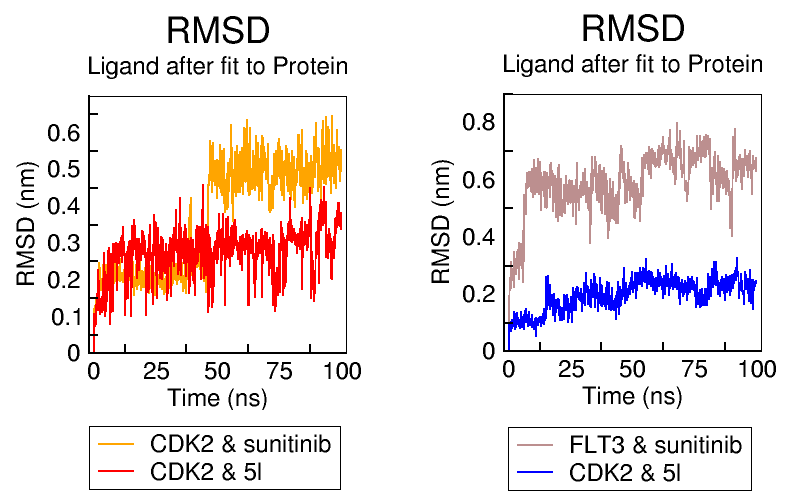


**Figure S1.** RMSD profiles for compound **5l** and sunitinib for each target CDK2 and FLT3 complexes, indicating deviations from initial conformations.


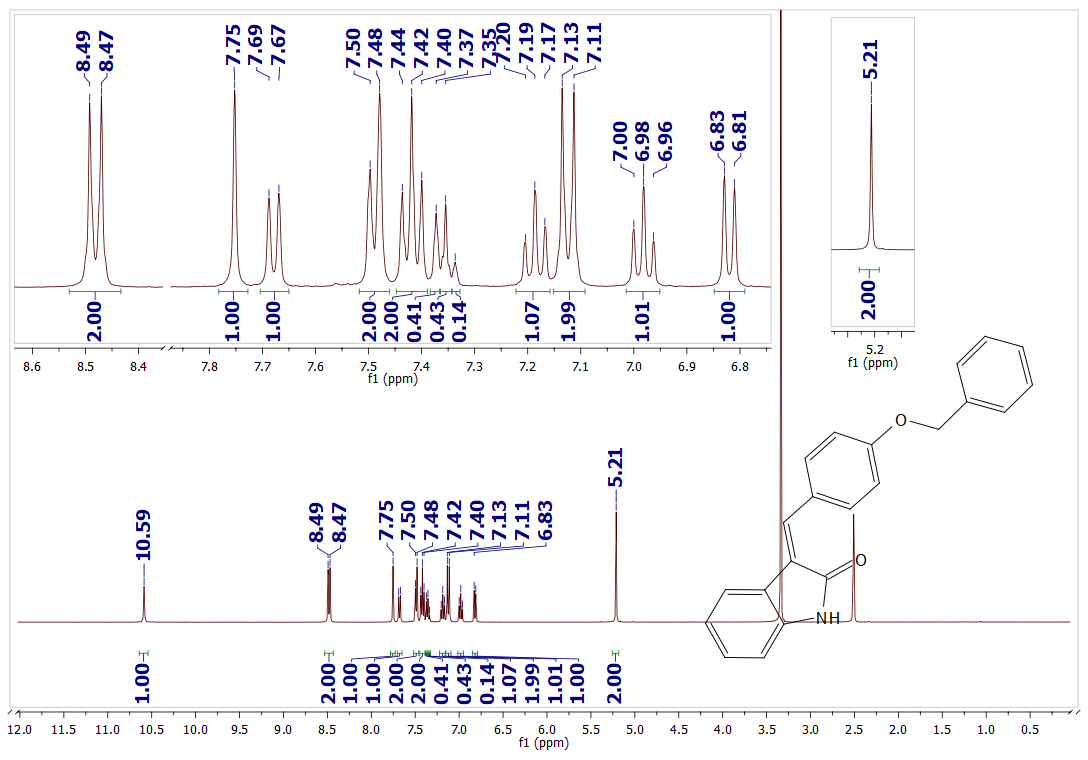


Figure S2. ^1^H NMR spectrum of compound (***E/Z***)-**5a**


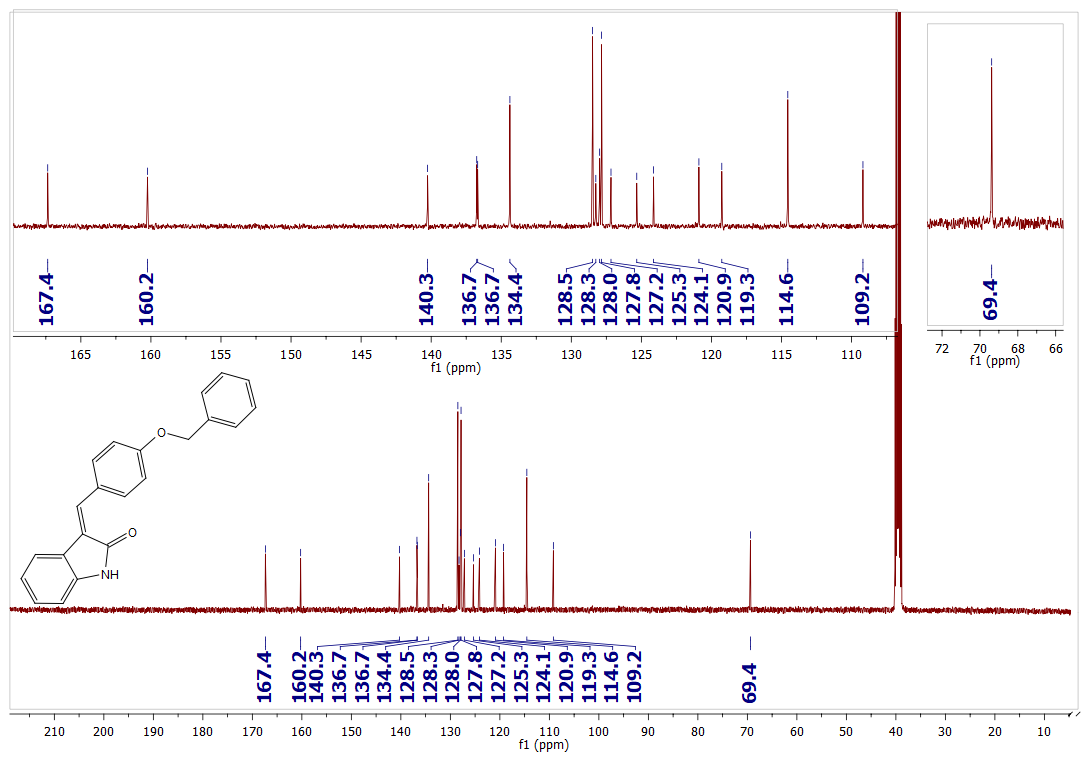


Figure S3. ^13^C NMR spectrum of compound (***E/Z***)-**5a**.


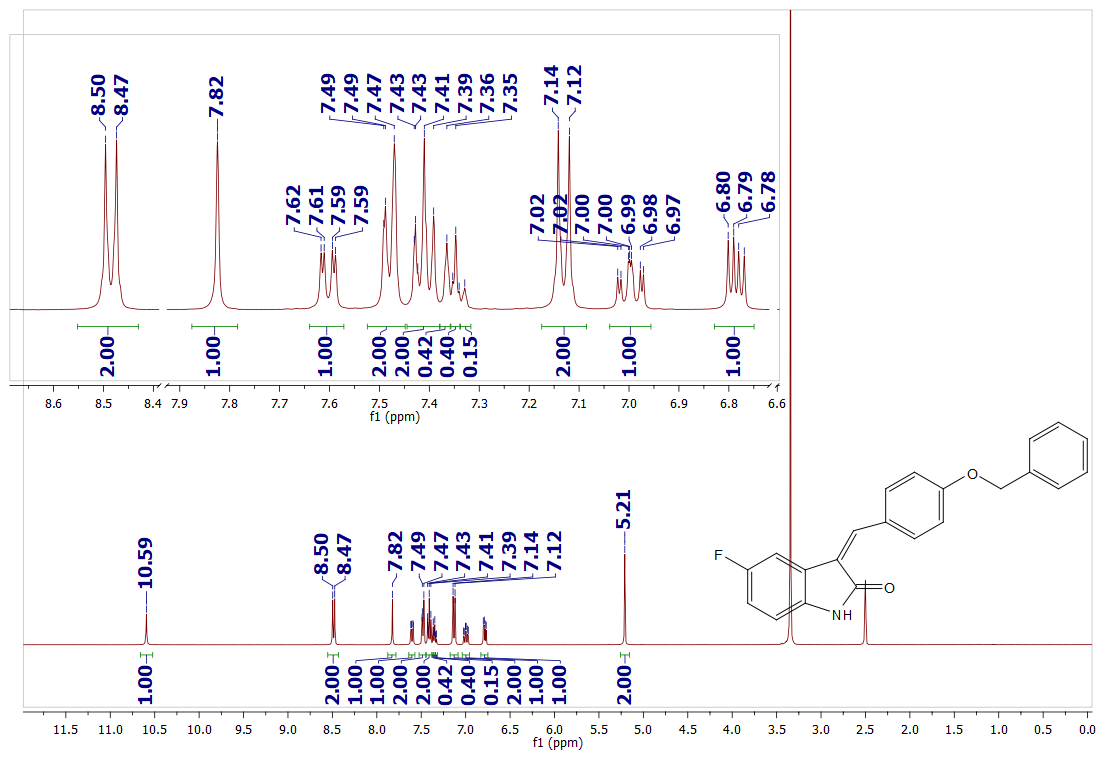


Figure S4. ^1^H NMR spectrum of compound (***E/Z***)-**5b**.


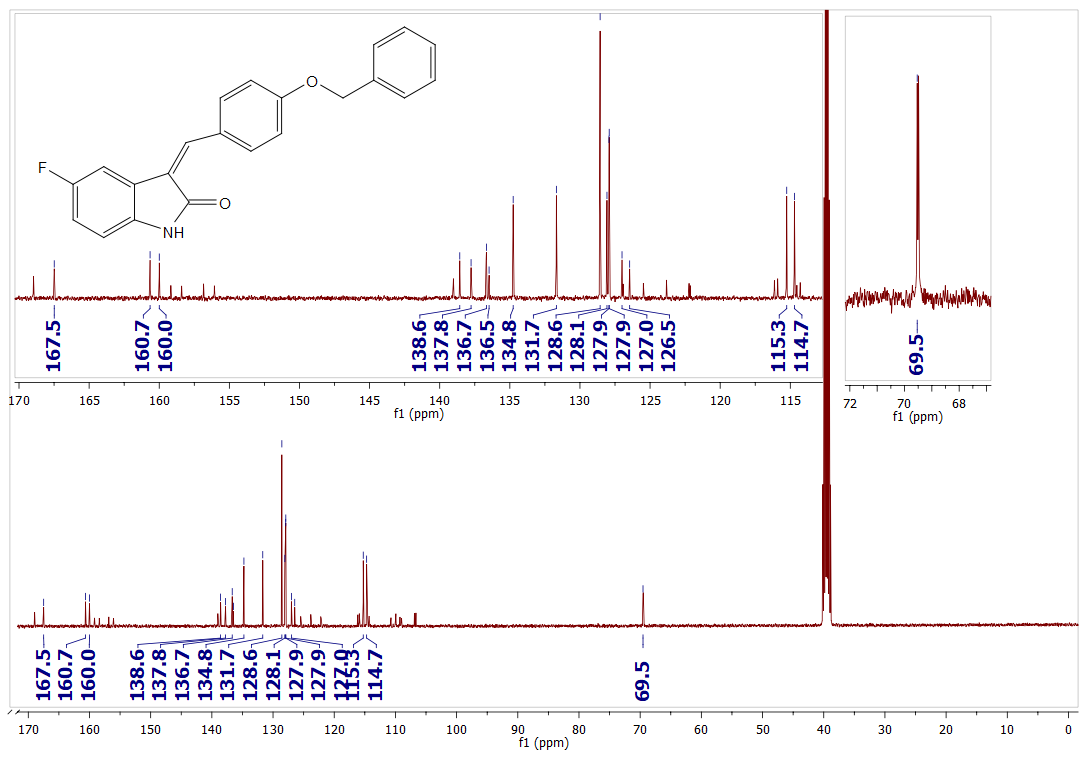


Figure S5. ^13^C NMR spectrum of compound (***E/Z***)-**5b.**


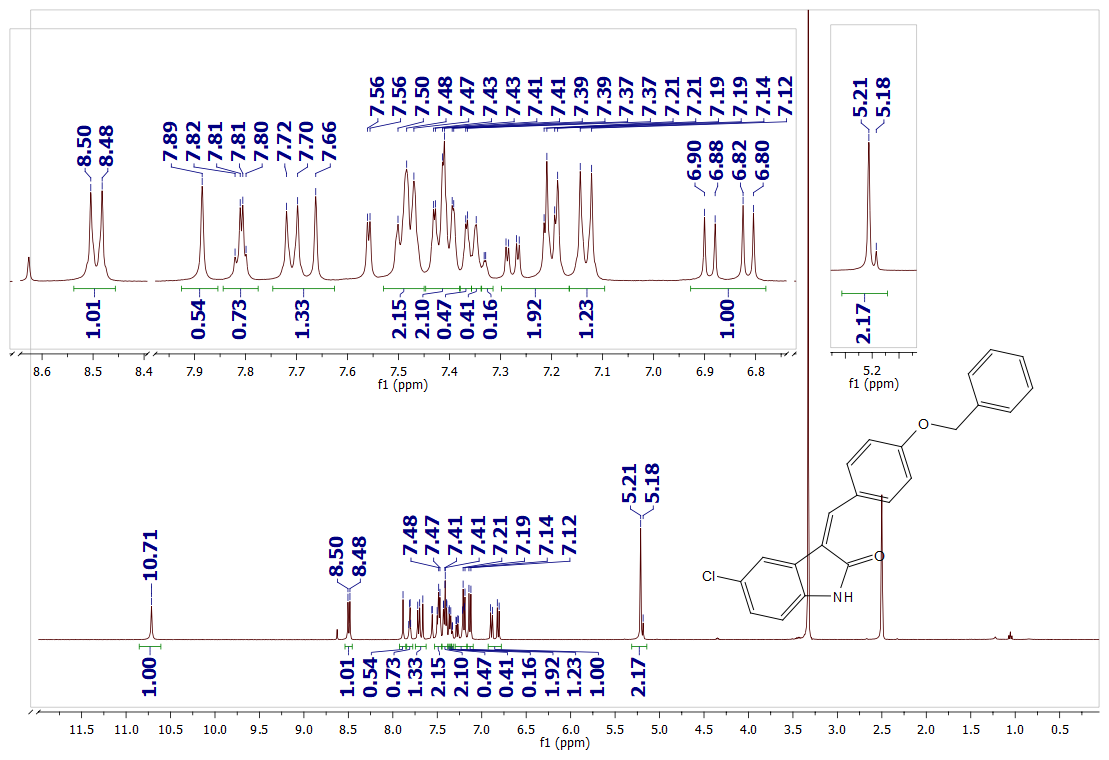


Figure S6. ^1^H NMR spectrum of compound (***E/Z***)-**5c**.


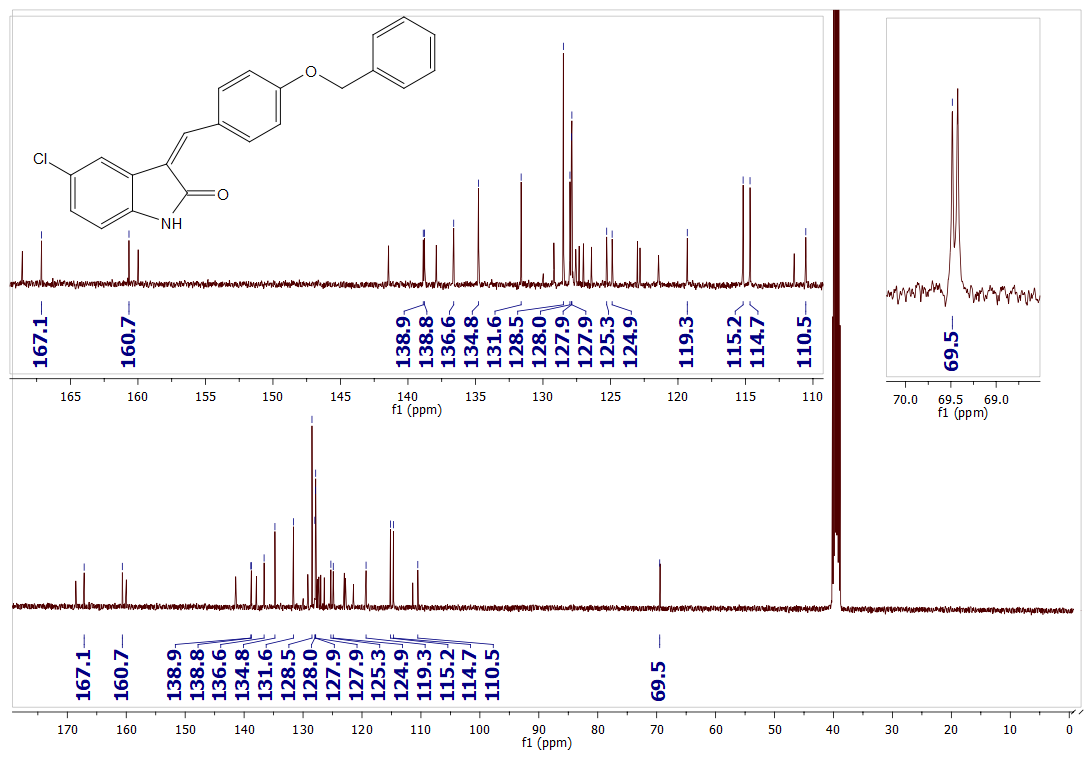


Figure S7. ^13^C NMR spectrum of compound (***E/Z***)-**5c.**


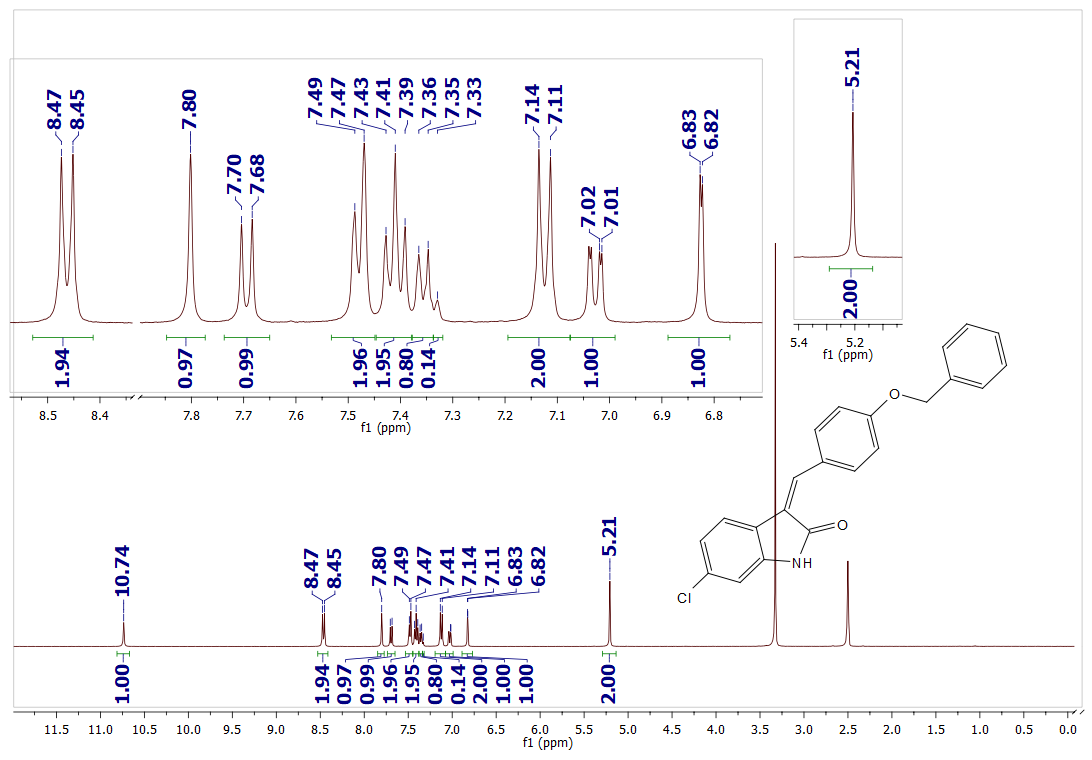


Figure S8. ^1^H NMR spectrum of compound (***E/Z***)-**5d**.


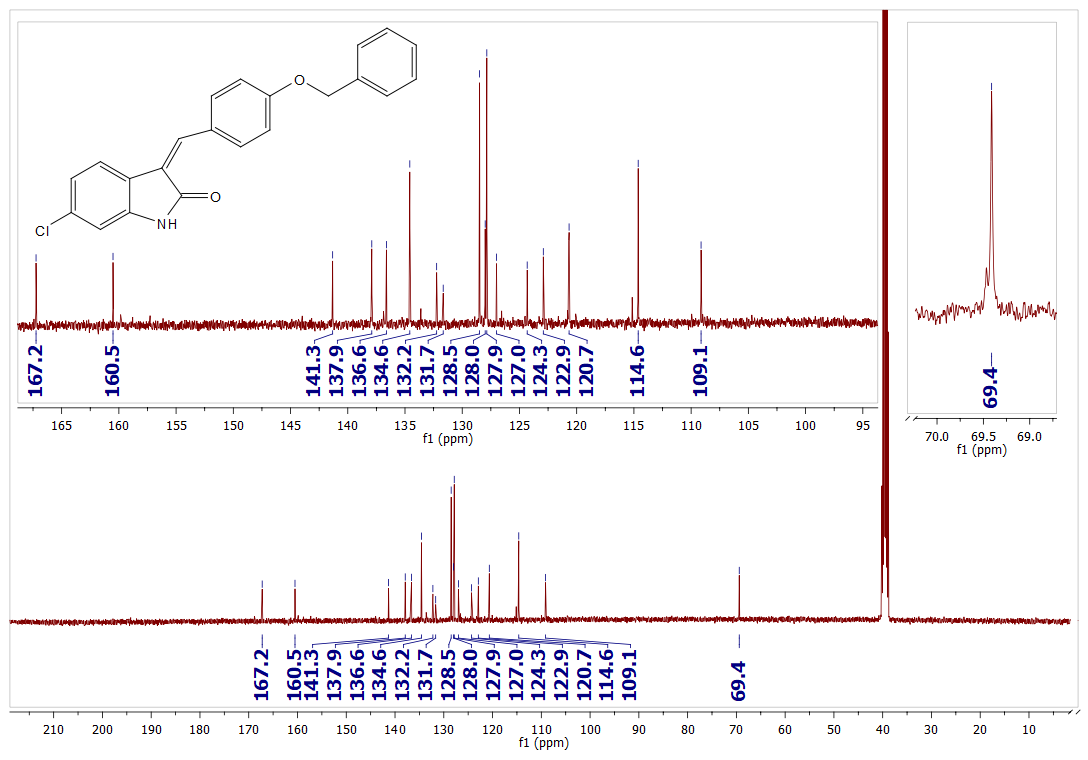


Figure S9. ^13^C NMR spectrum of compound (***E/Z***)-**5d.**


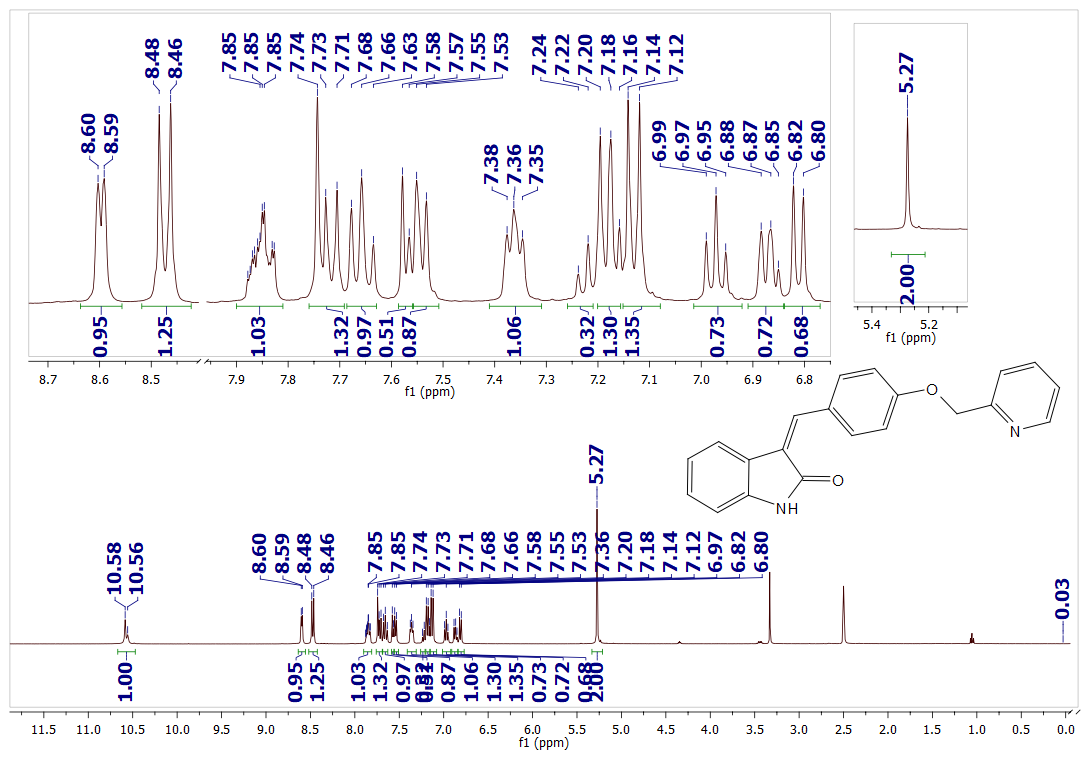


Figure S10. ^1^H NMR spectrum of compound (***E/Z***)-**5e**.


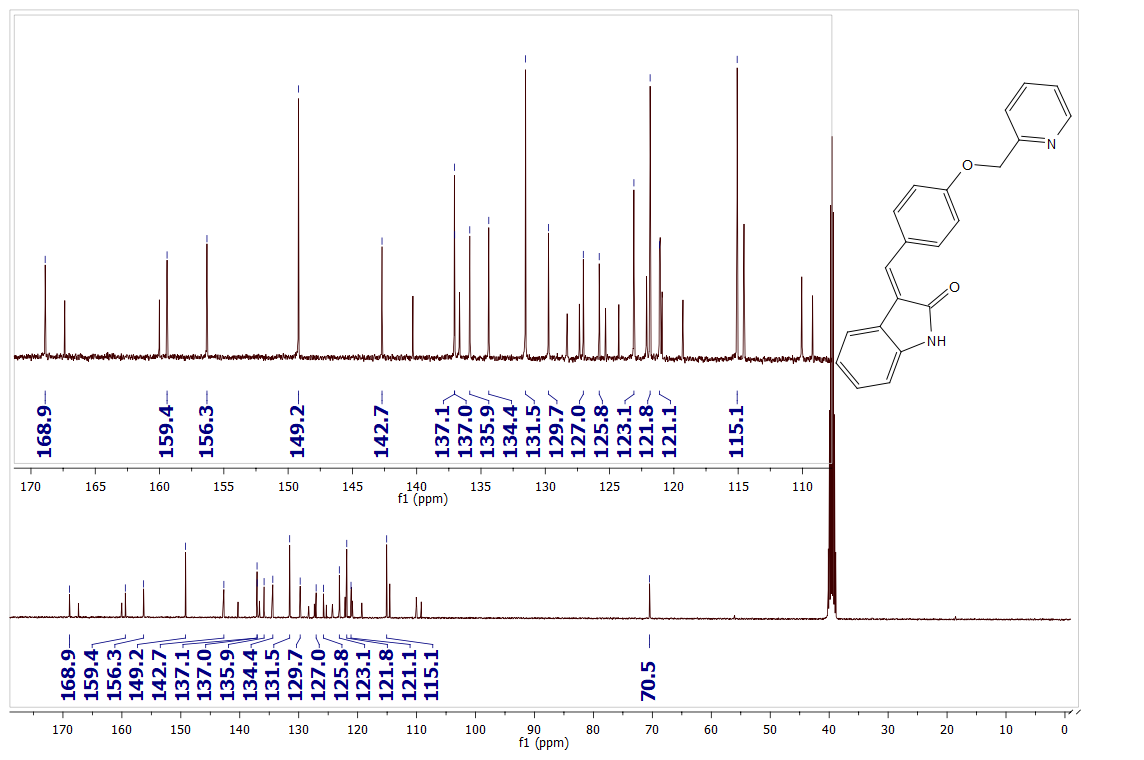


Figure S11. ^13^C NMR spectrum of compound (***E/Z***)-**5e.**


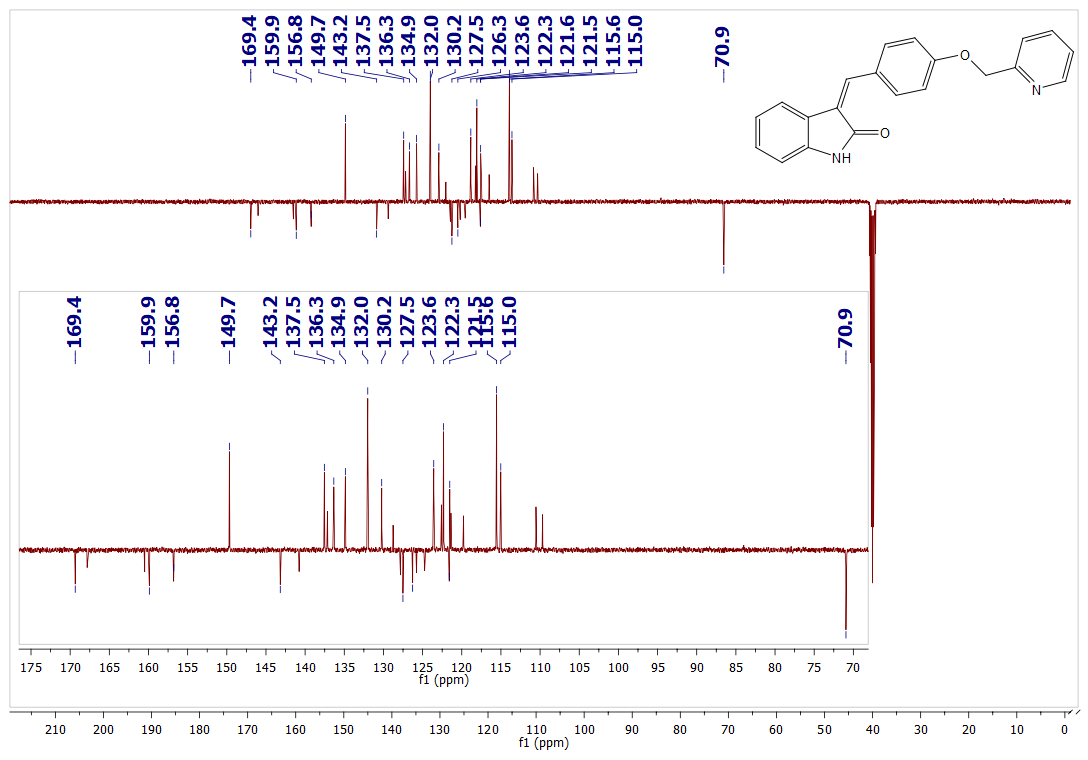


Figure S12. DEPTQ ^13^C NMR spectrum of compound (***E/Z***)-**5e.**


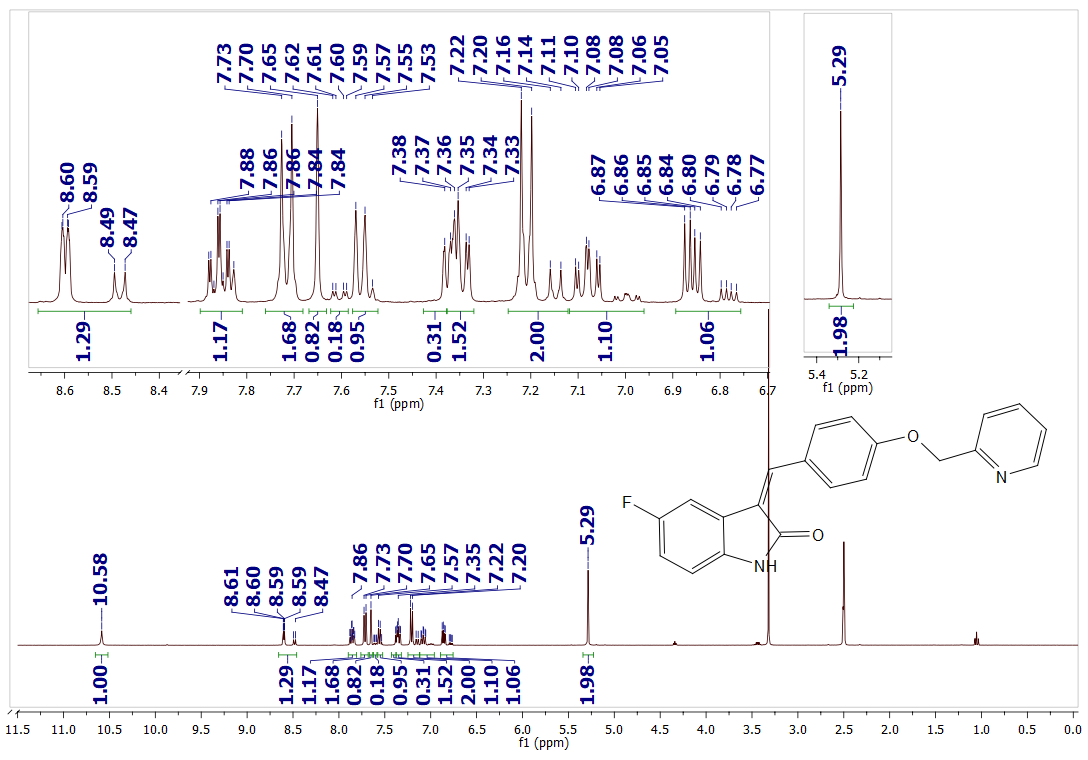


Figure S13. ^1^H NMR spectrum of compound (***E/Z***)-**5f**


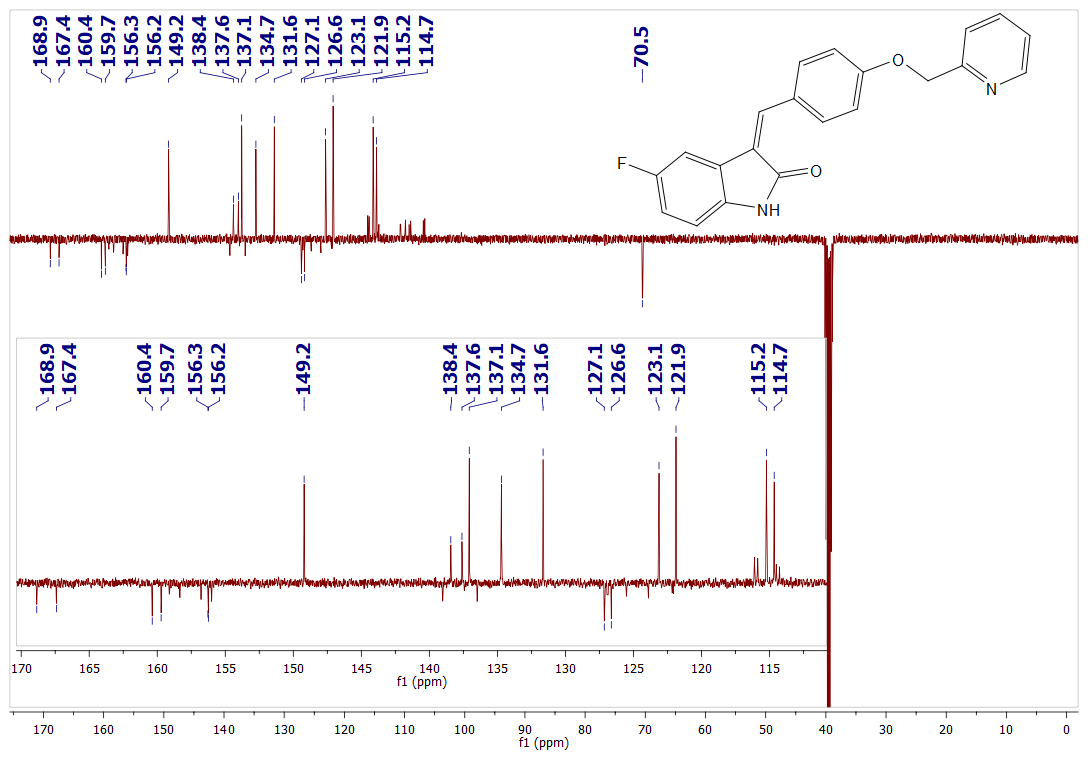


Figure S14. DEPTQ ^13^C NMR spectrum of compound (***E/Z***)-**5f.**


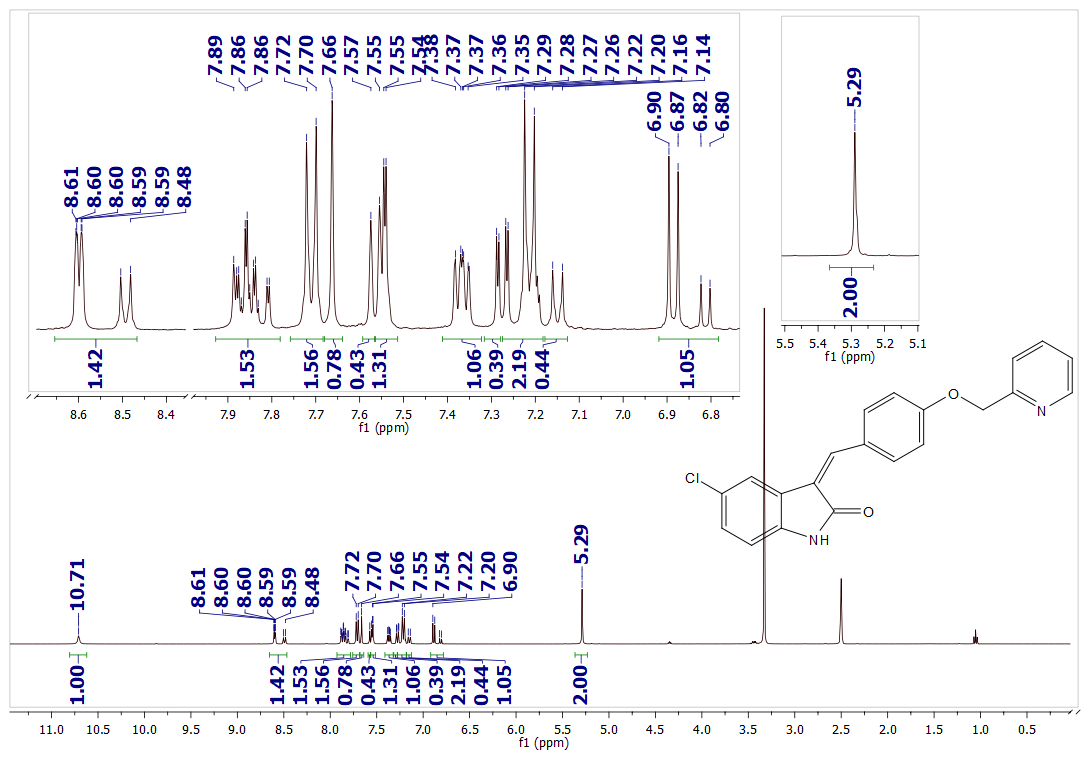


Figure S15. ^1^H NMR spectrum of compound (***E/Z***)-**5g**


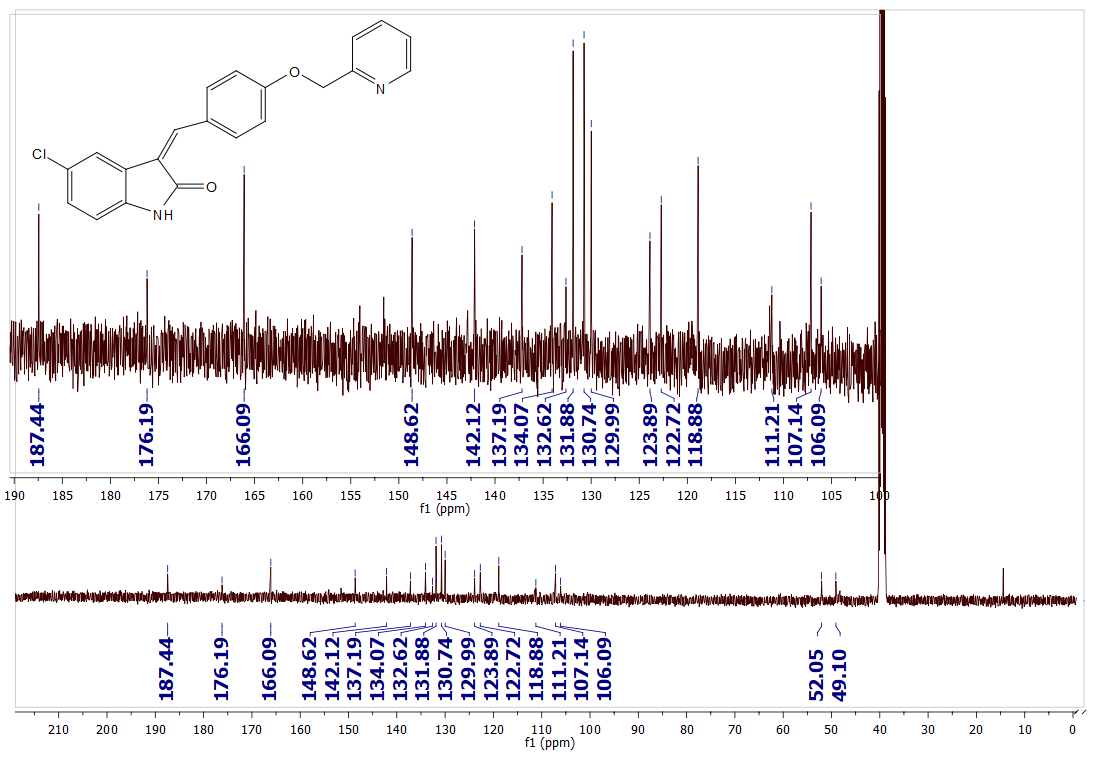


Figure S16. ^13^C NMR spectrum of compound (***E/Z***)-**5g.**


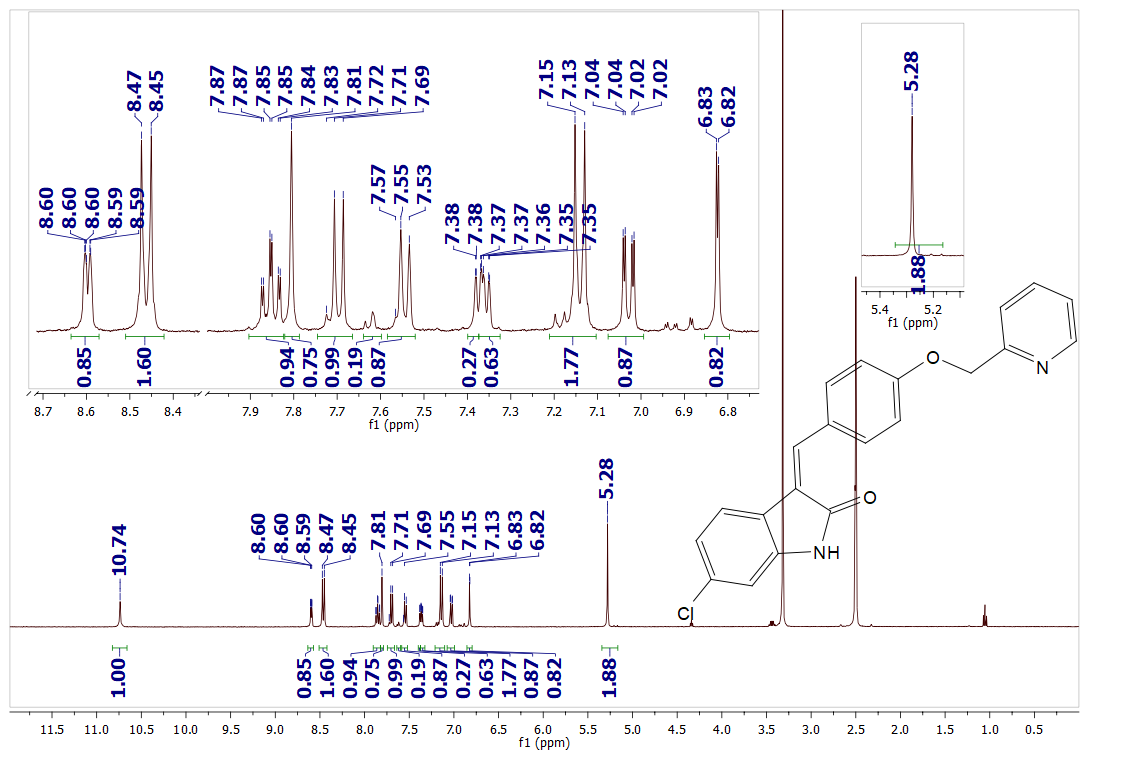


Figure S17. ^1^H NMR spectrum of compound (***E/Z***)-**5g**


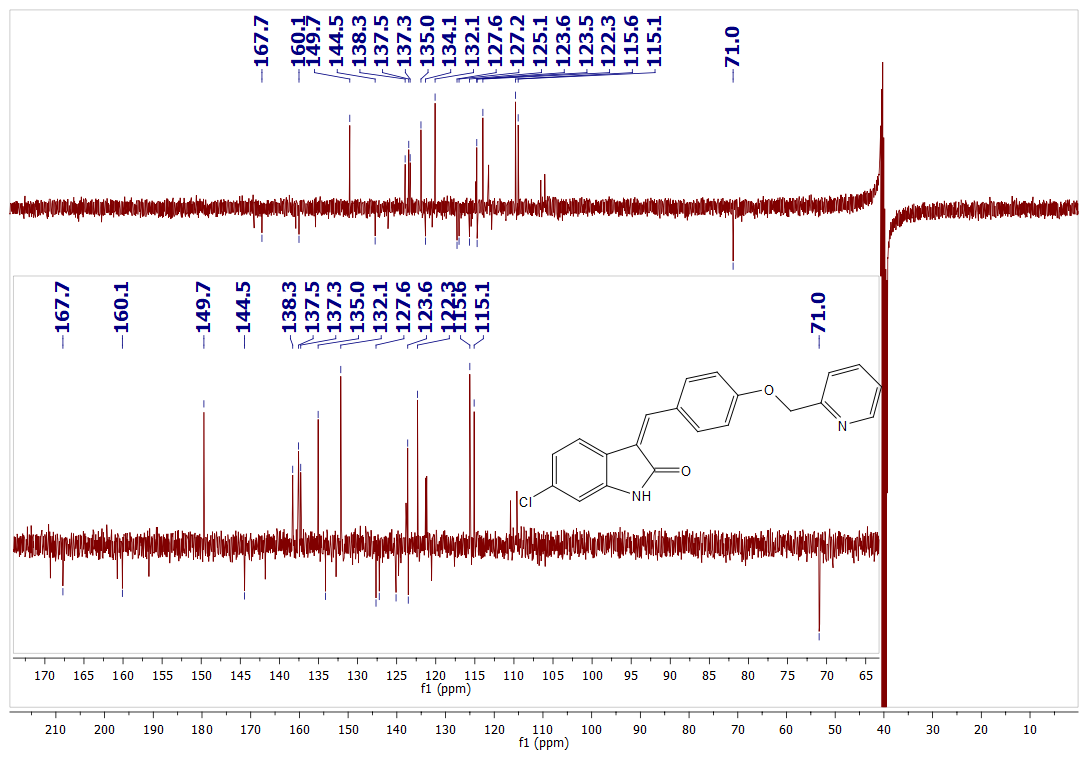


Figure S18. DEPTQ ^13^C NMR spectrum of compound (***E/Z***)-**5h.**


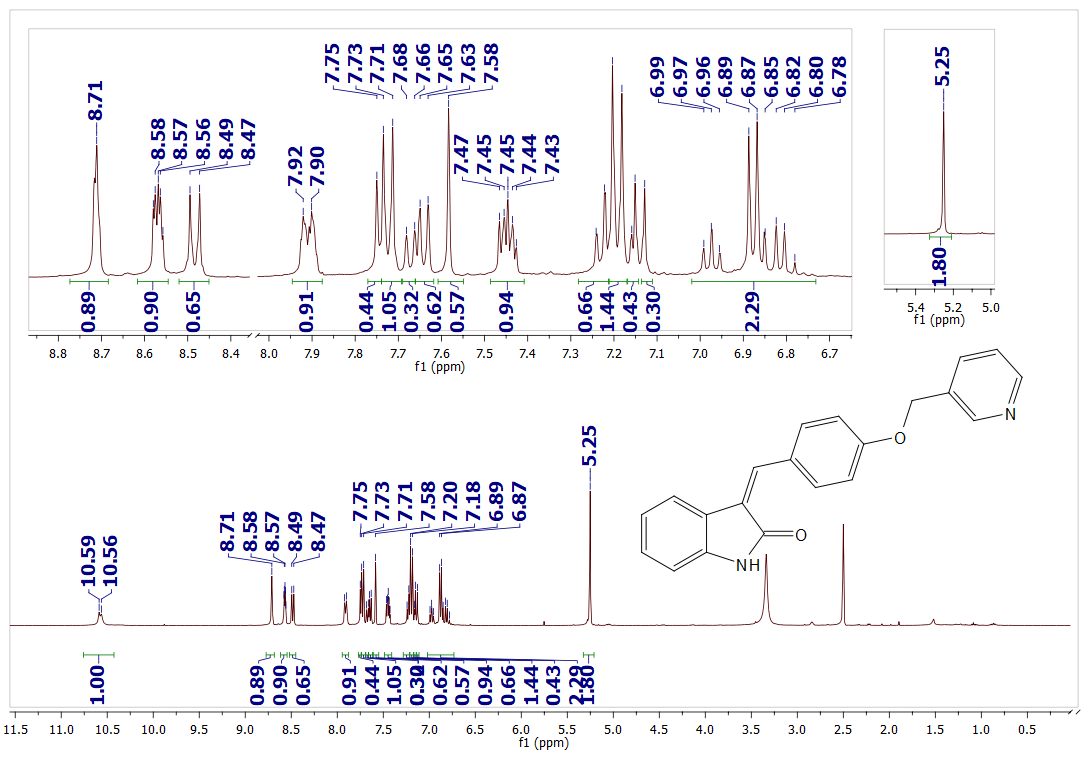


Figure S19. ^1^H NMR spectrum of compound (***E/Z***)-**5i.**


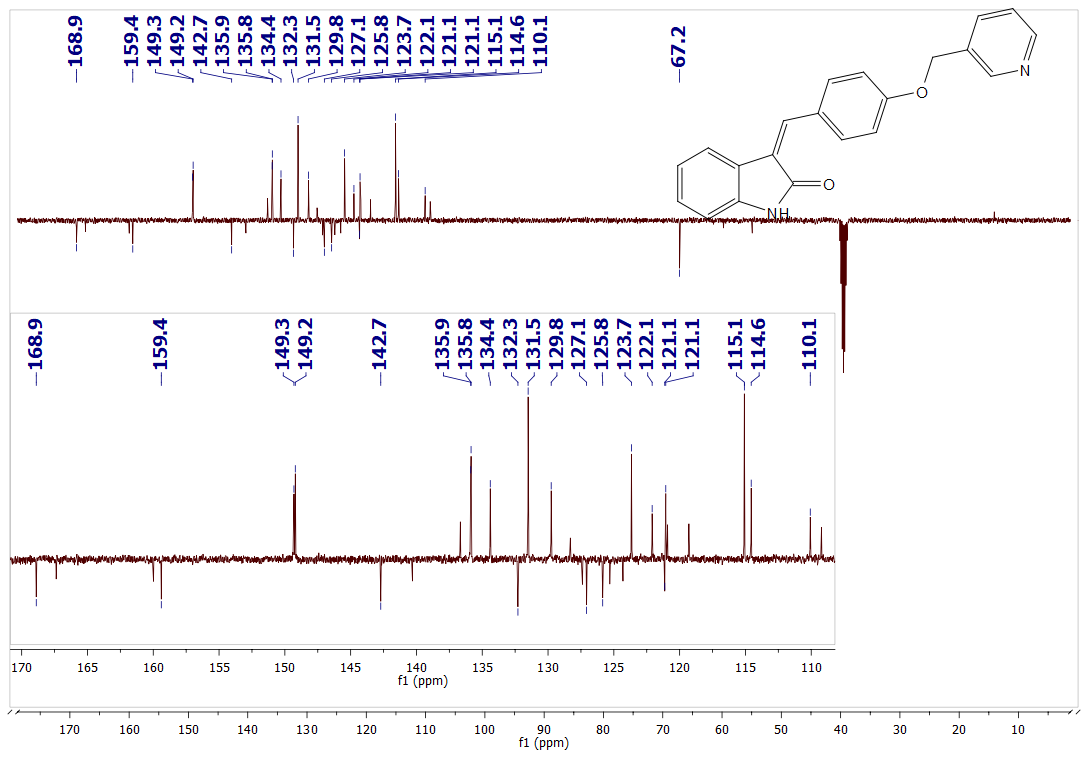


Figure S20. DEPTQ ^13^C NMR spectrum of compound (***E/Z***)-**5i.**


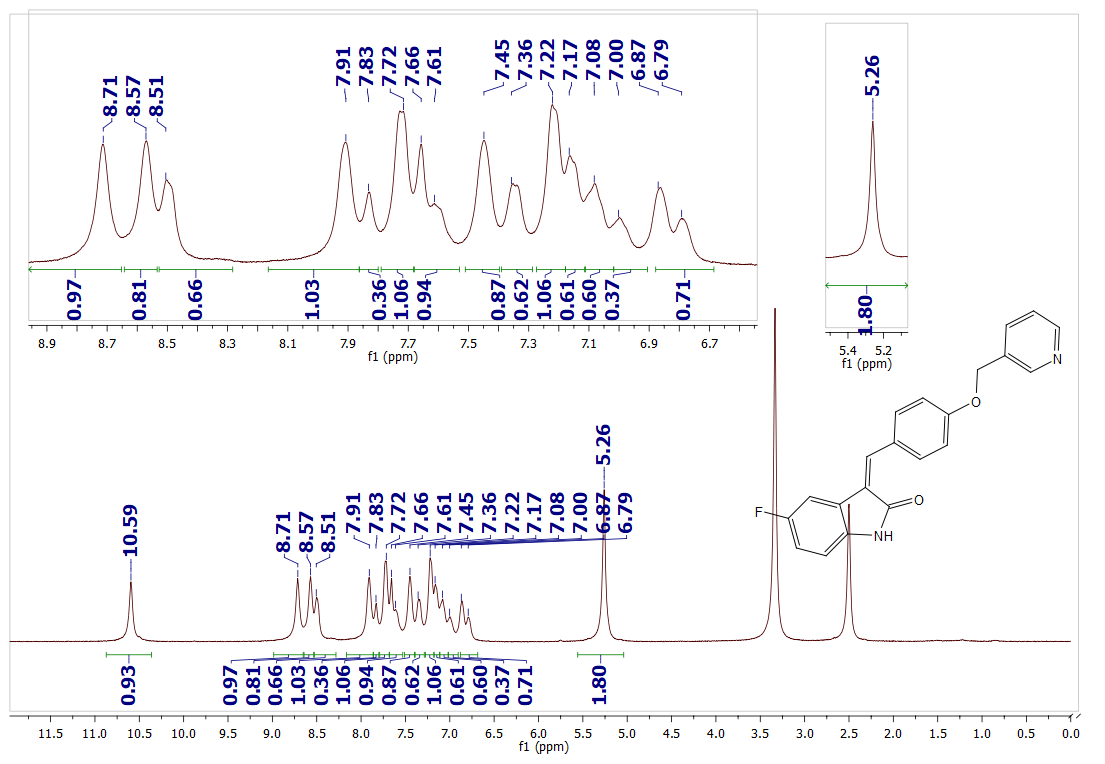


Figure S21. ^1^H NMR spectrum of compound (***E/Z***)-**5j.**


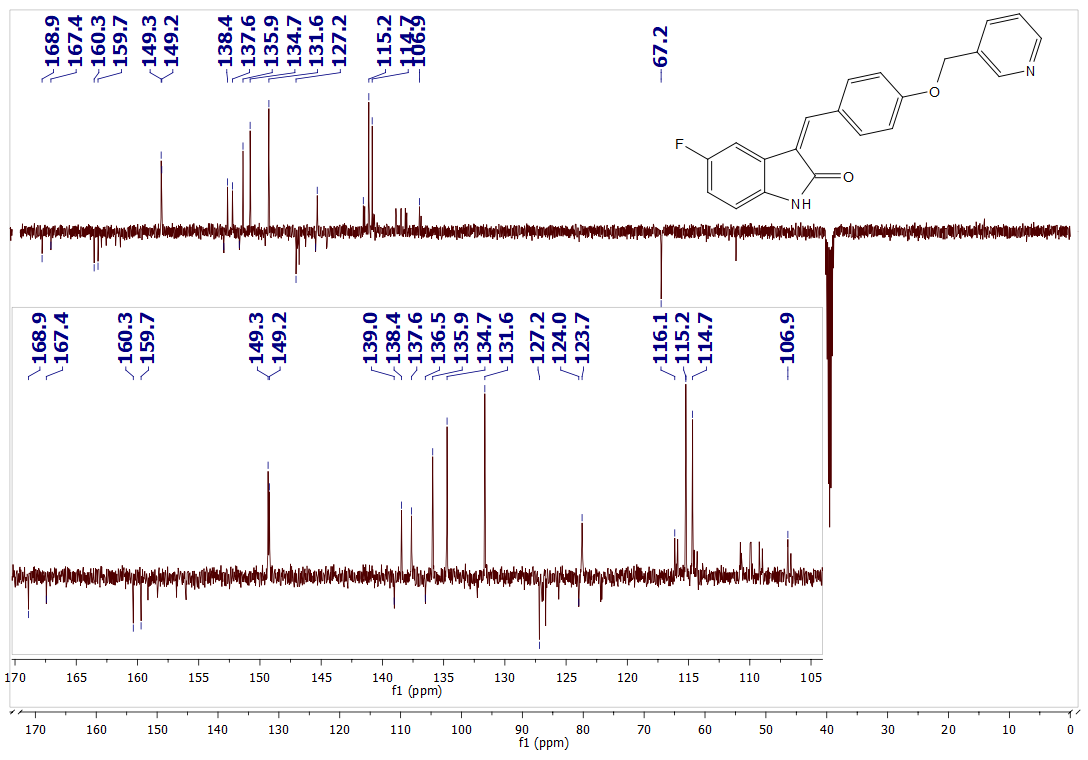


Figure S22. DEPTQ ^13^C NMR spectrum of compound (***E/Z***)-**5j.**


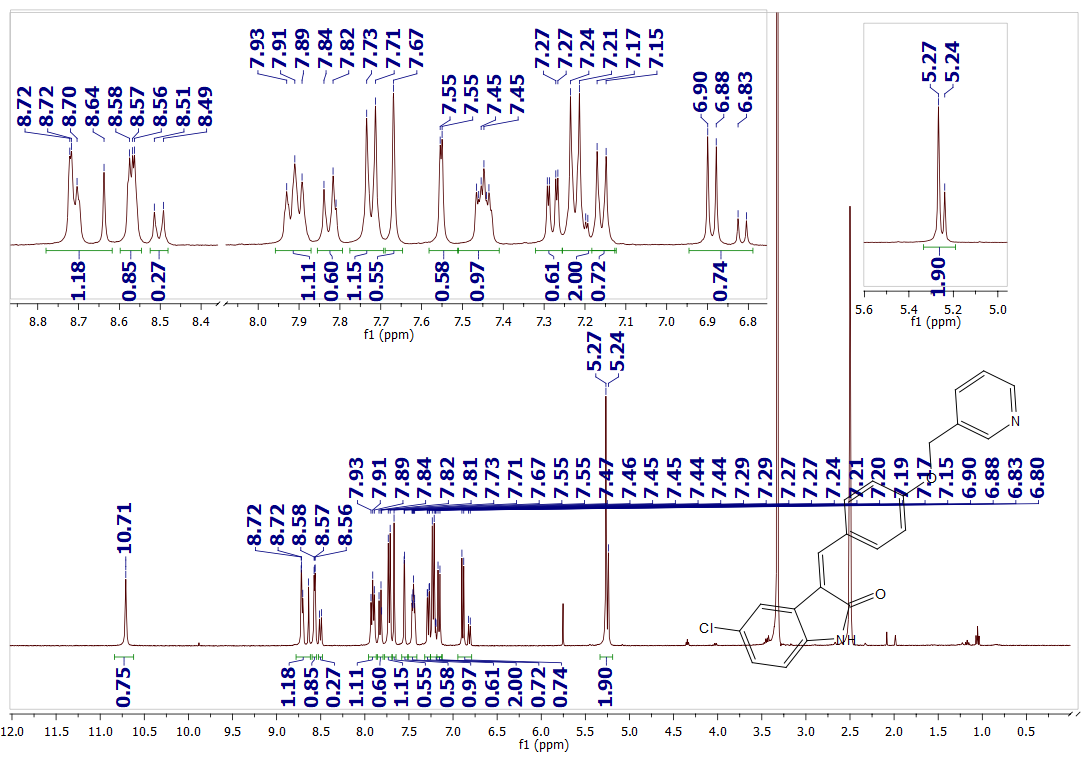


Figure S23. ^1^H NMR spectrum of compound (***E/Z***)-**5k.**


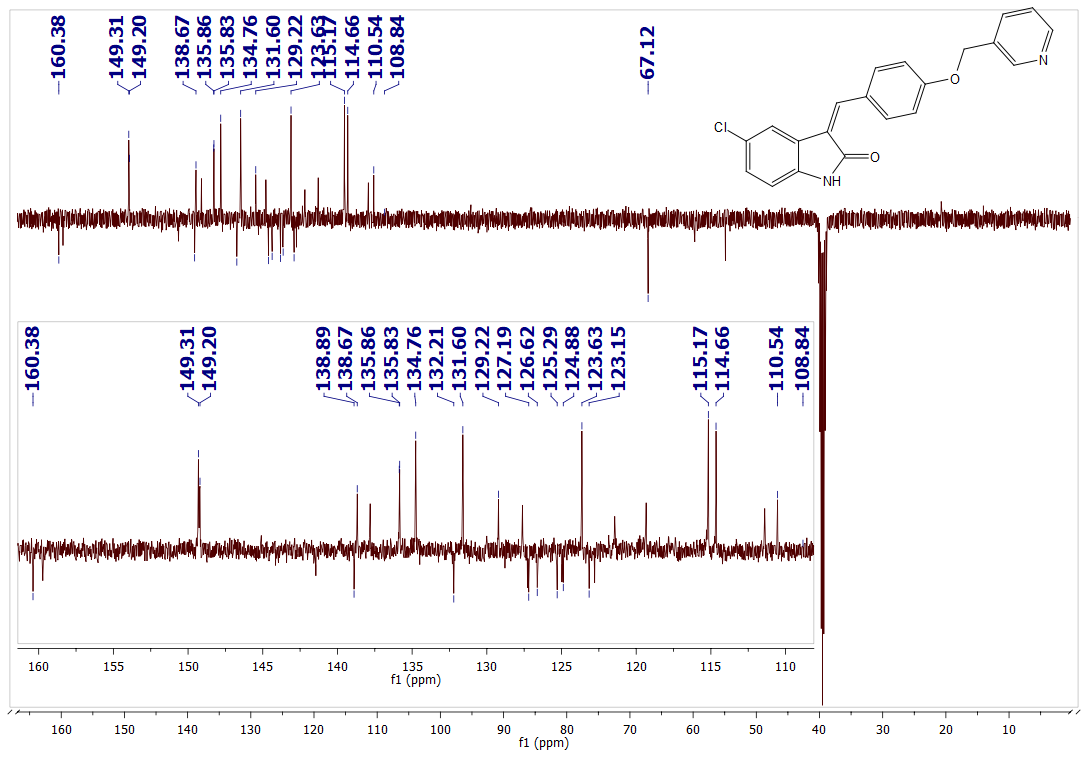


Figure S24. DEPTQ ^13^C NMR spectrum of compound (***E/Z***)-**5k.**


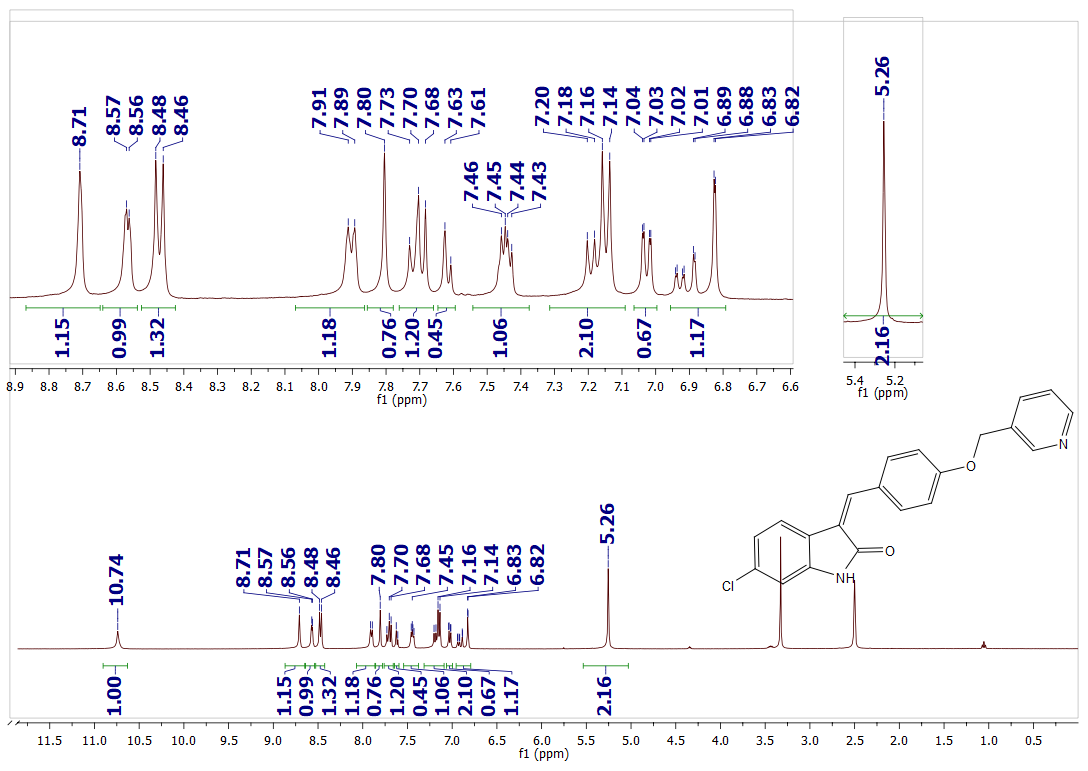


Figure S25. ^1^H NMR spectrum of compound (***E/Z***)-**5l.**


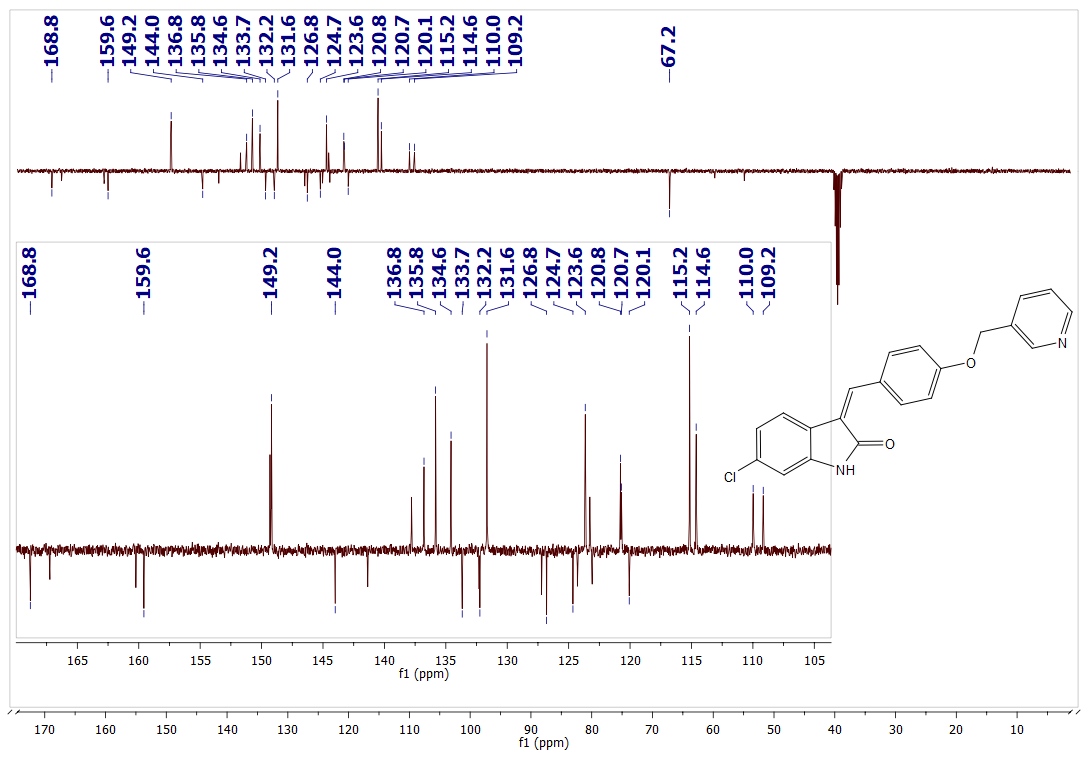


Figure S26. DEPTQ ^13^C NMR spectrum of compound (***E/Z***)-**5l.**


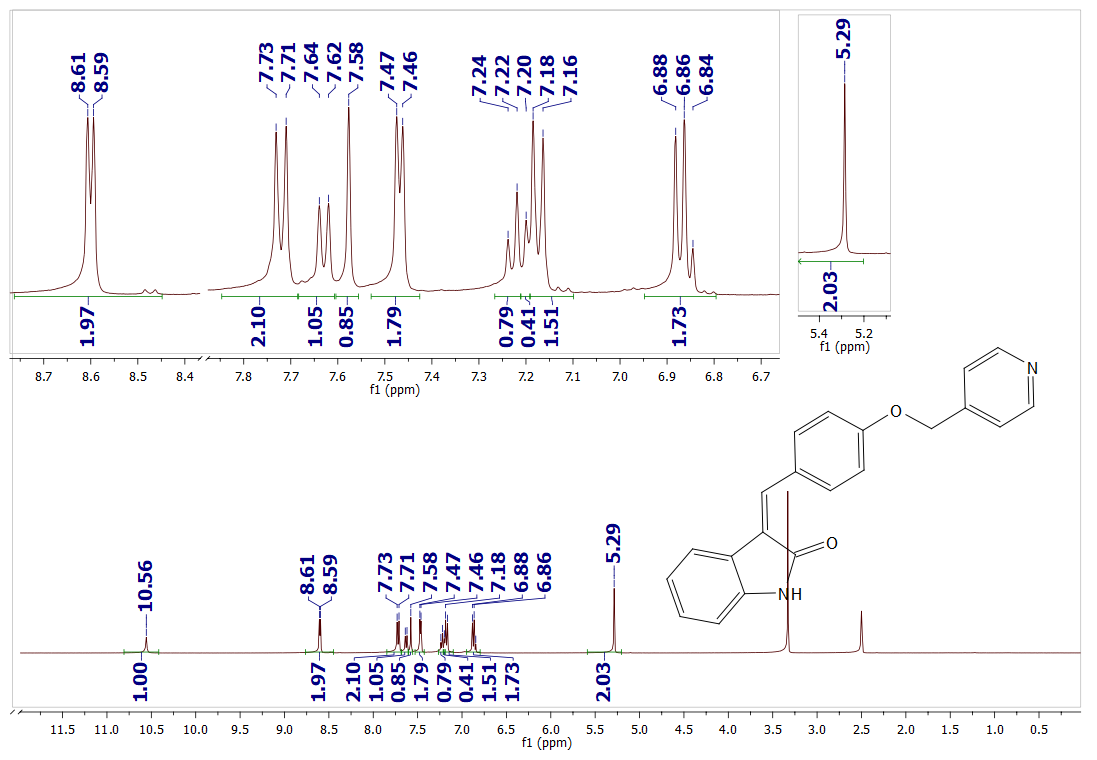


Figure S27. ^1^H NMR spectrum of compound (***E/Z***)-**5m.**


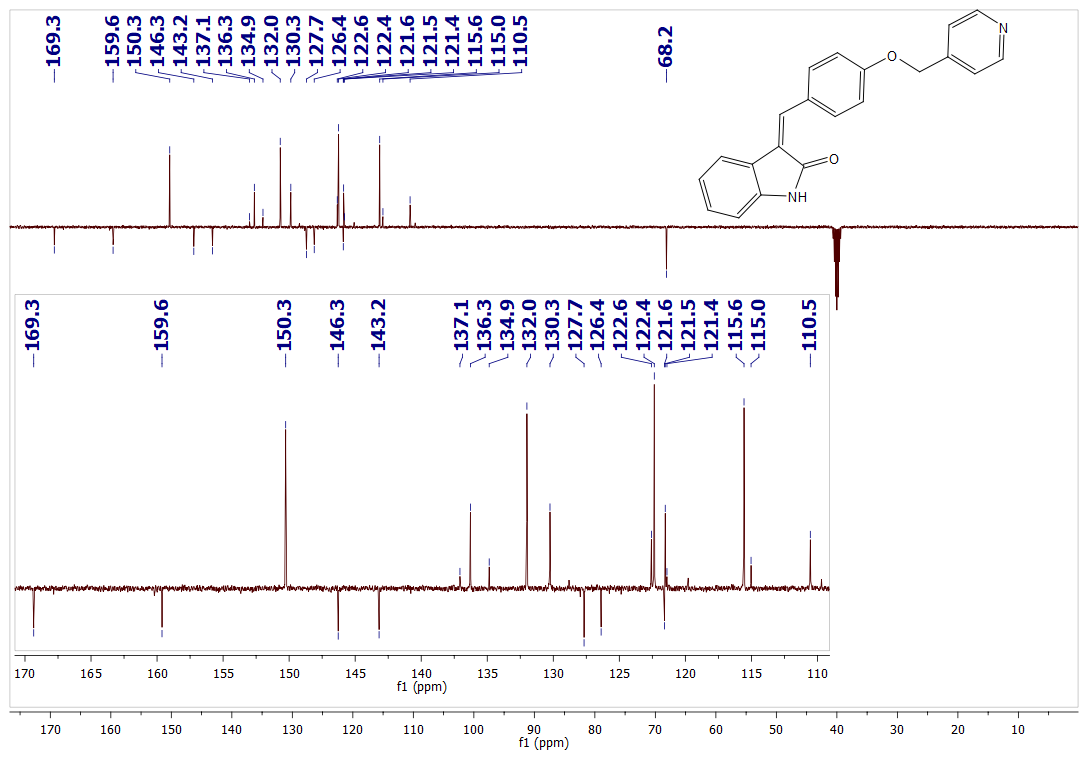


Figure S28. DEPTQ ^13^C NMR spectrum of compound (***E/Z***)-**5m.**


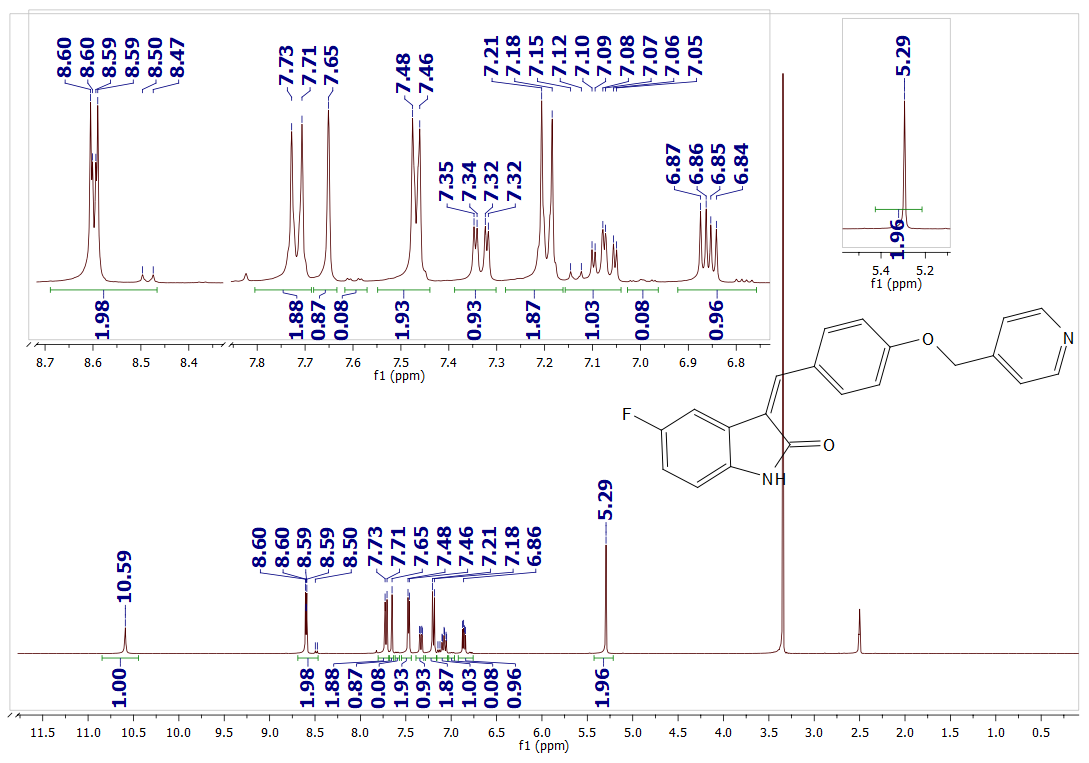


Figure S29. ^1^H NMR spectrum of compound (***E/Z***)-**5n.**


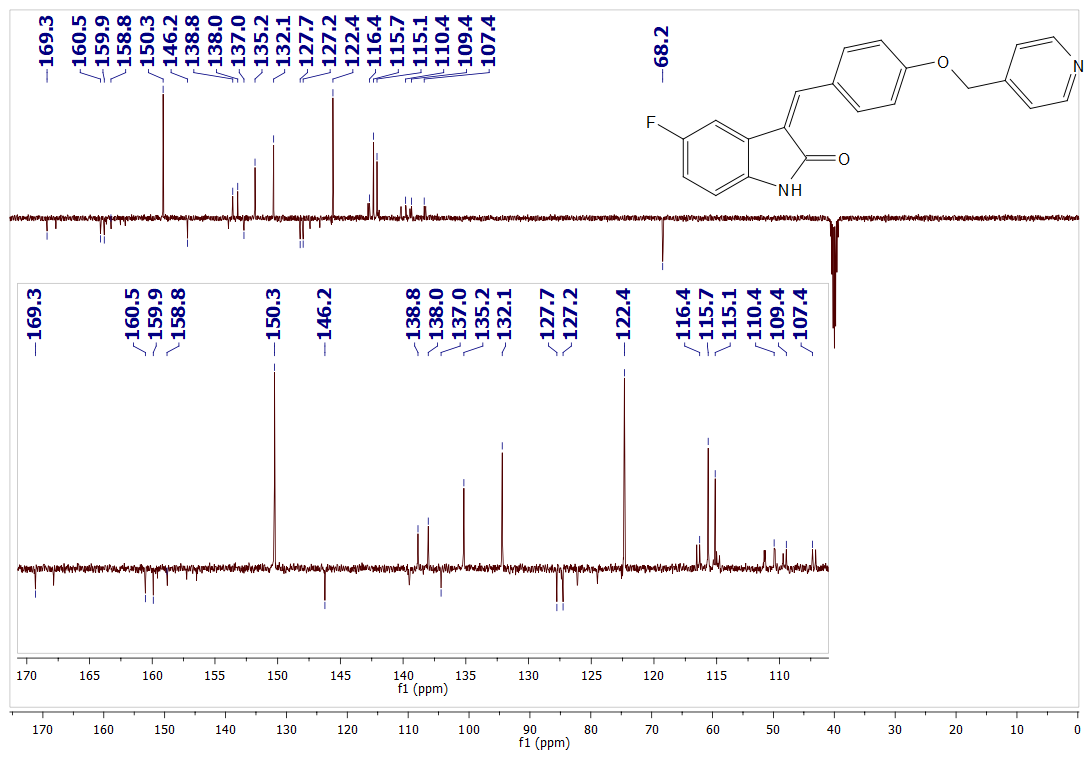


Figure S30. DEPTQ ^13^C NMR spectrum of compound (***E/Z***)-**5n.**


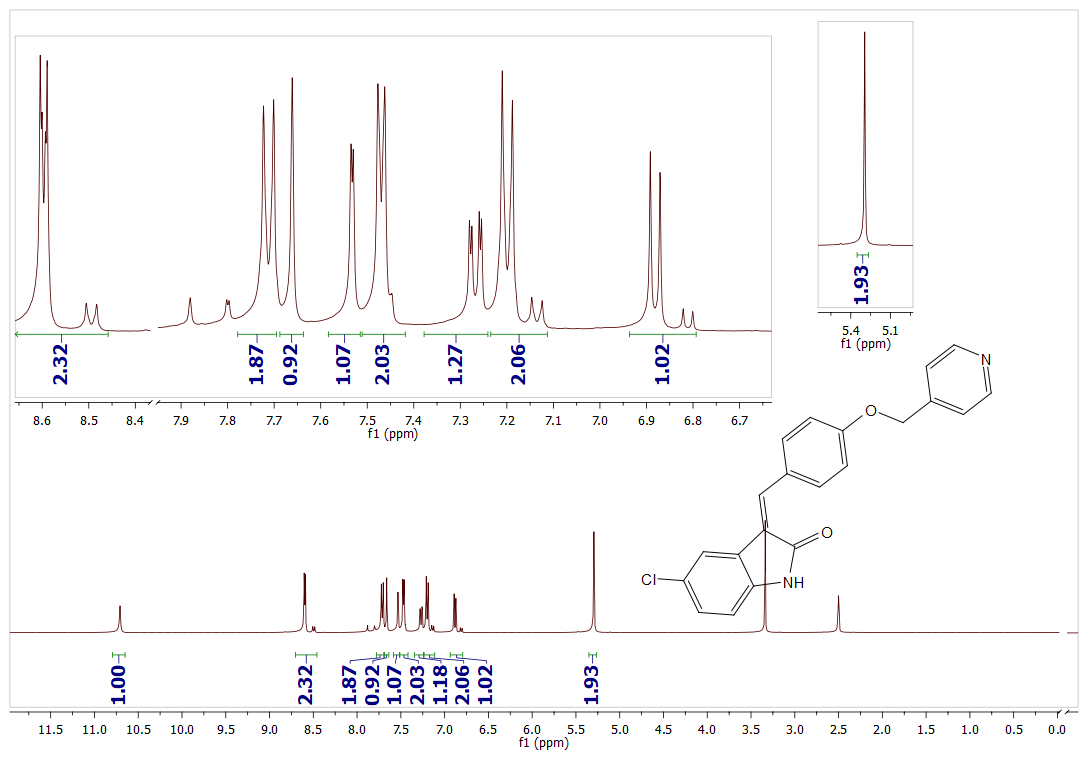


Figure S31. ^1^H NMR spectrum of compound (***E/Z***)-**5o.**


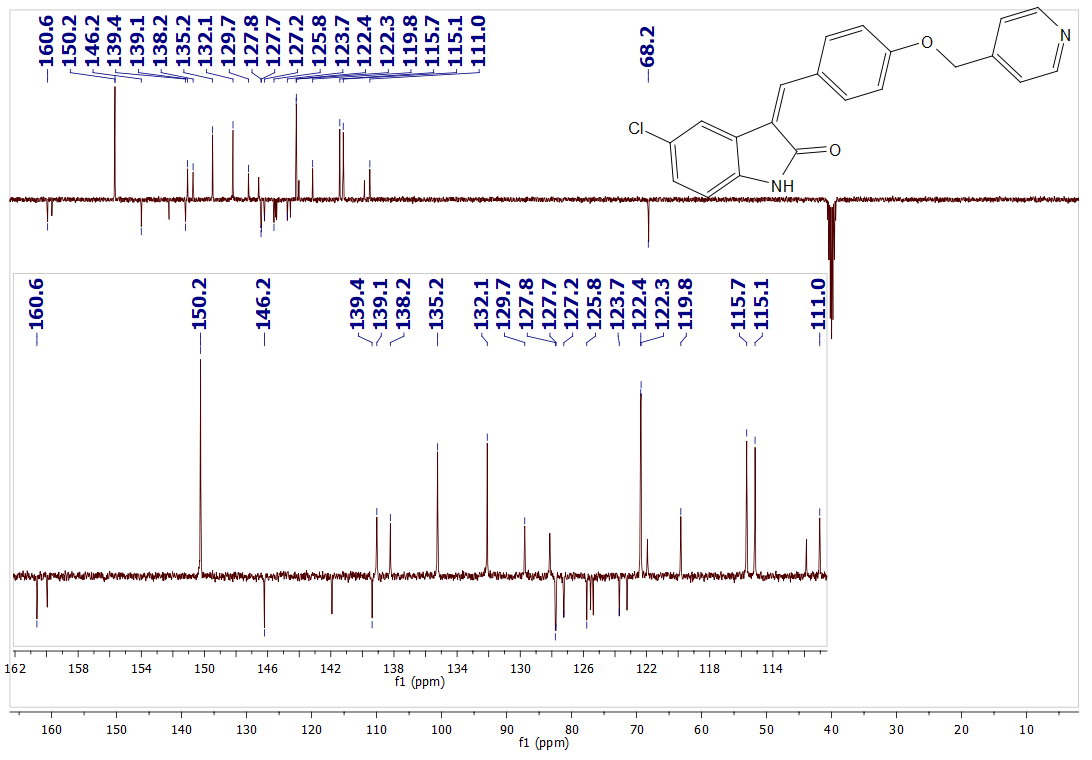


Figure S32. DEPTQ ^13^C NMR spectrum of compound (***E/Z***)-**5o.**


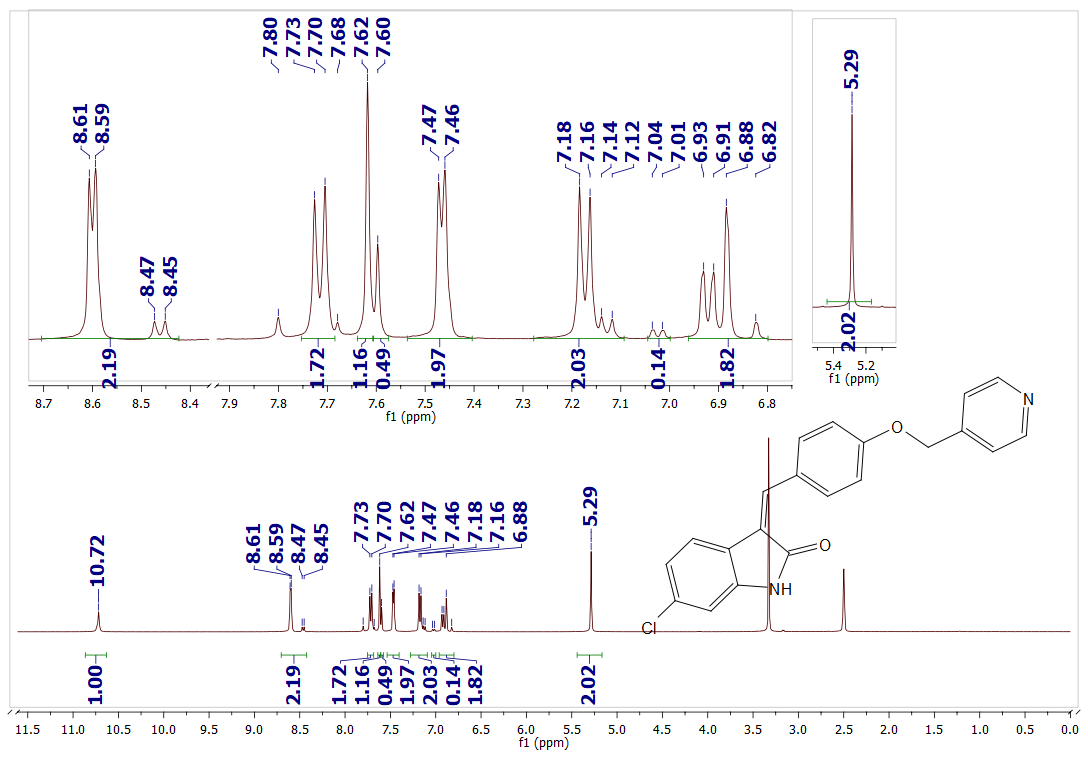


Figure S33. ^1^H NMR spectrum of compound (***E/Z***)-**5p.**


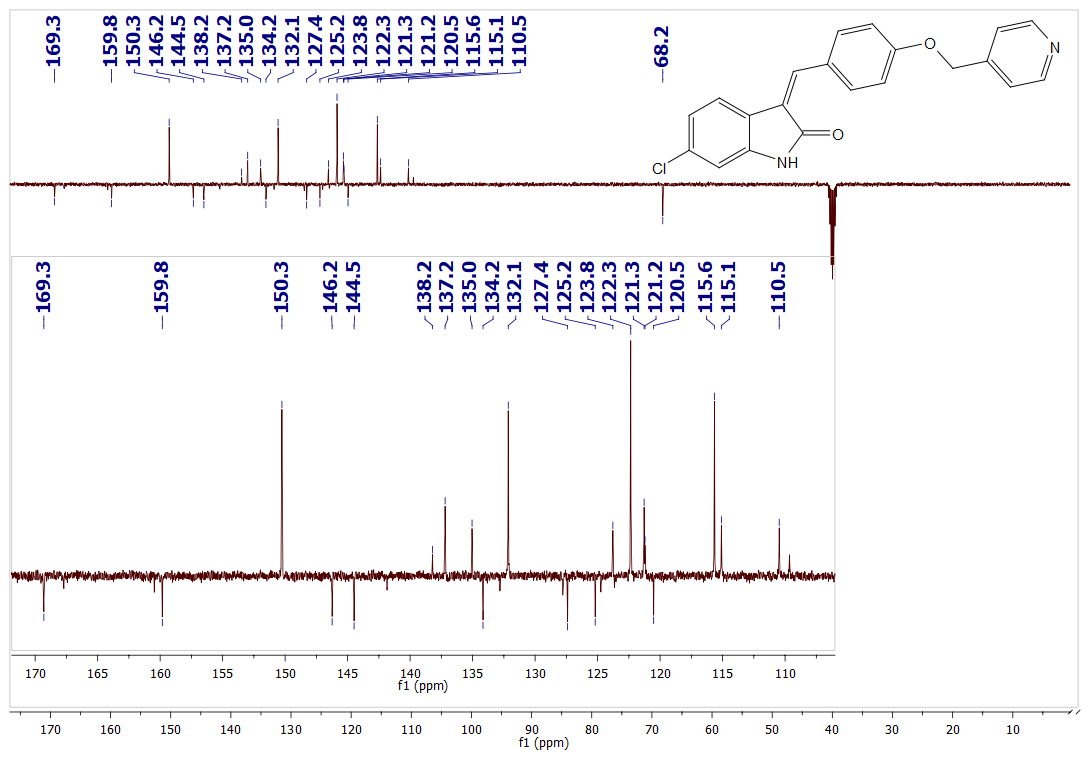


Figure S34. DEPTQ ^13^C NMR spectrum of compound (***E/Z***)-**5p.**
