# Supplementary material for: Discovery and Anticancer Screening of Novel Oxindole-Based Derivative Bearing Pyridyl Group as Potent and Selective Dual FLT3/CDK2 Kinase Inhibitor
Source: Pharmaceuticals (Basel). 2024 May 20;17(5):659. doi: 10.3390/ph17050659 (PMC11124822; doi:10.3390/ph17050659)
Supplement: Supplementary file 1 [file pharmaceuticals-17-00659-s001.zip › Supplementary Files/Supportting_Information_Videos_S1_S4.pptx]

## Slide 1
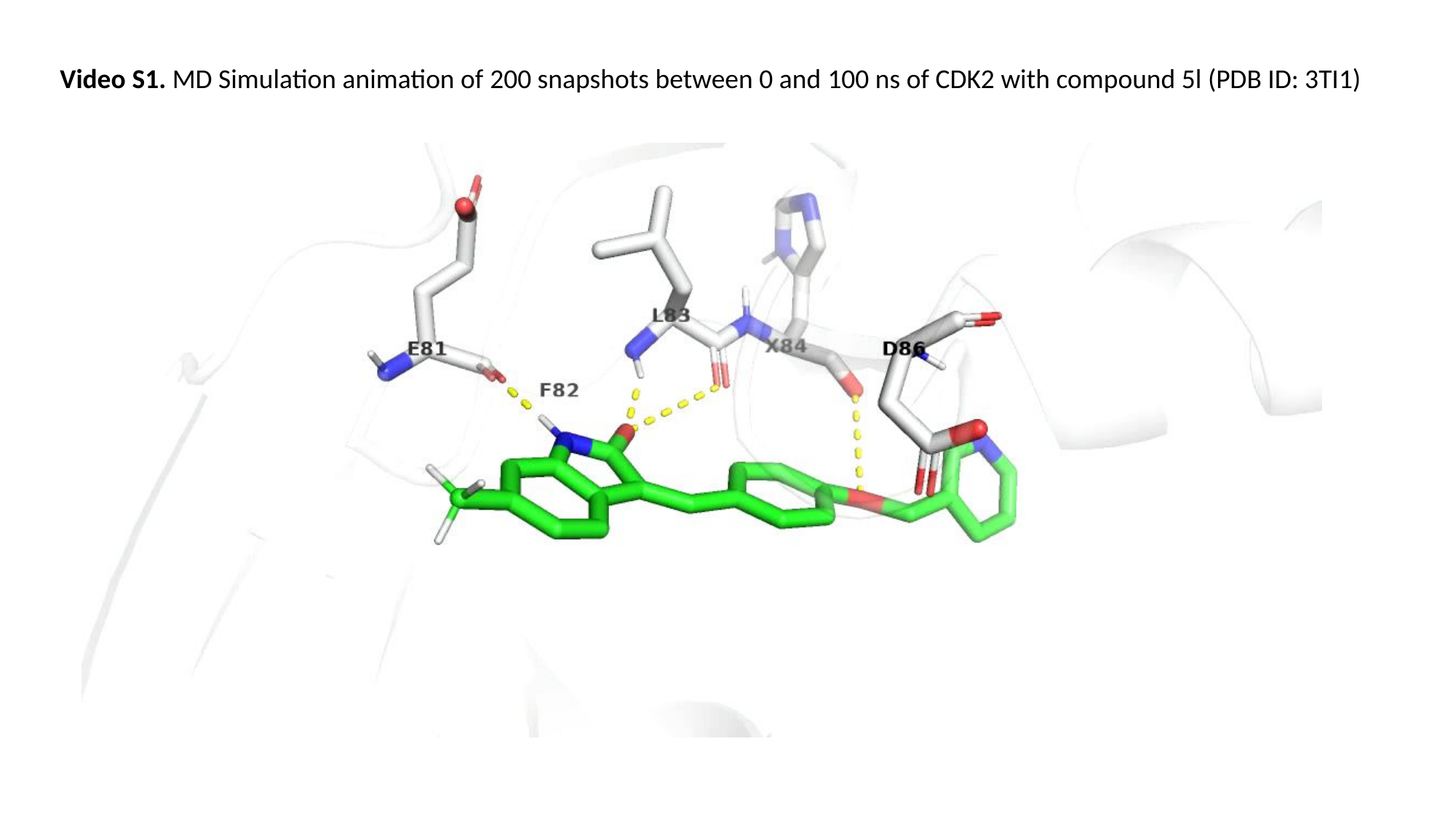

# Video S1. MD Simulation animation of 200 snapshots between 0 and 100 ns of CDK2 with compound 5l (PDB ID: 3TI1)

## Slide 2
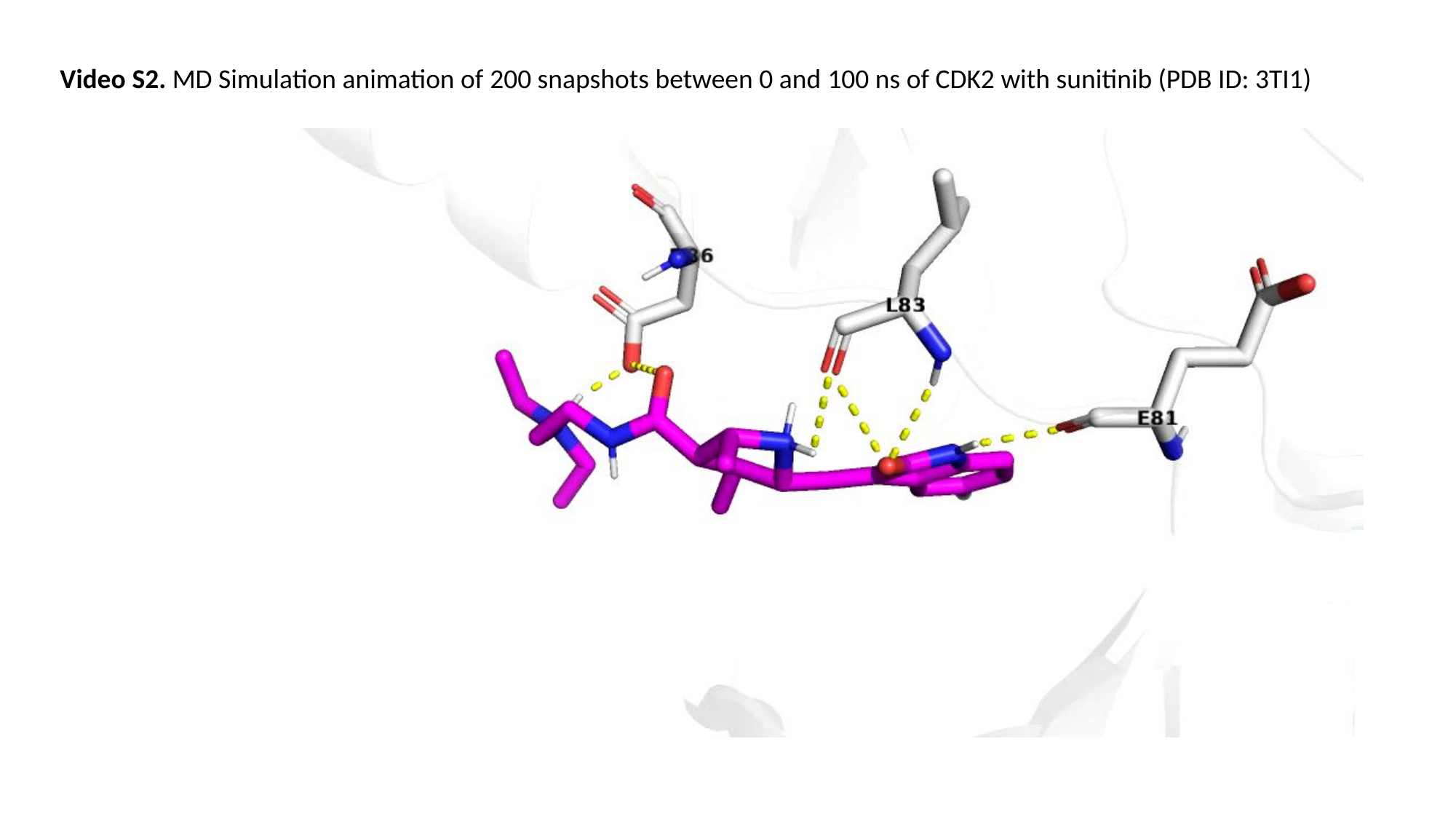

# Video S2. MD Simulation animation of 200 snapshots between 0 and 100 ns of CDK2 with sunitinib (PDB ID: 3TI1)

## Slide 3
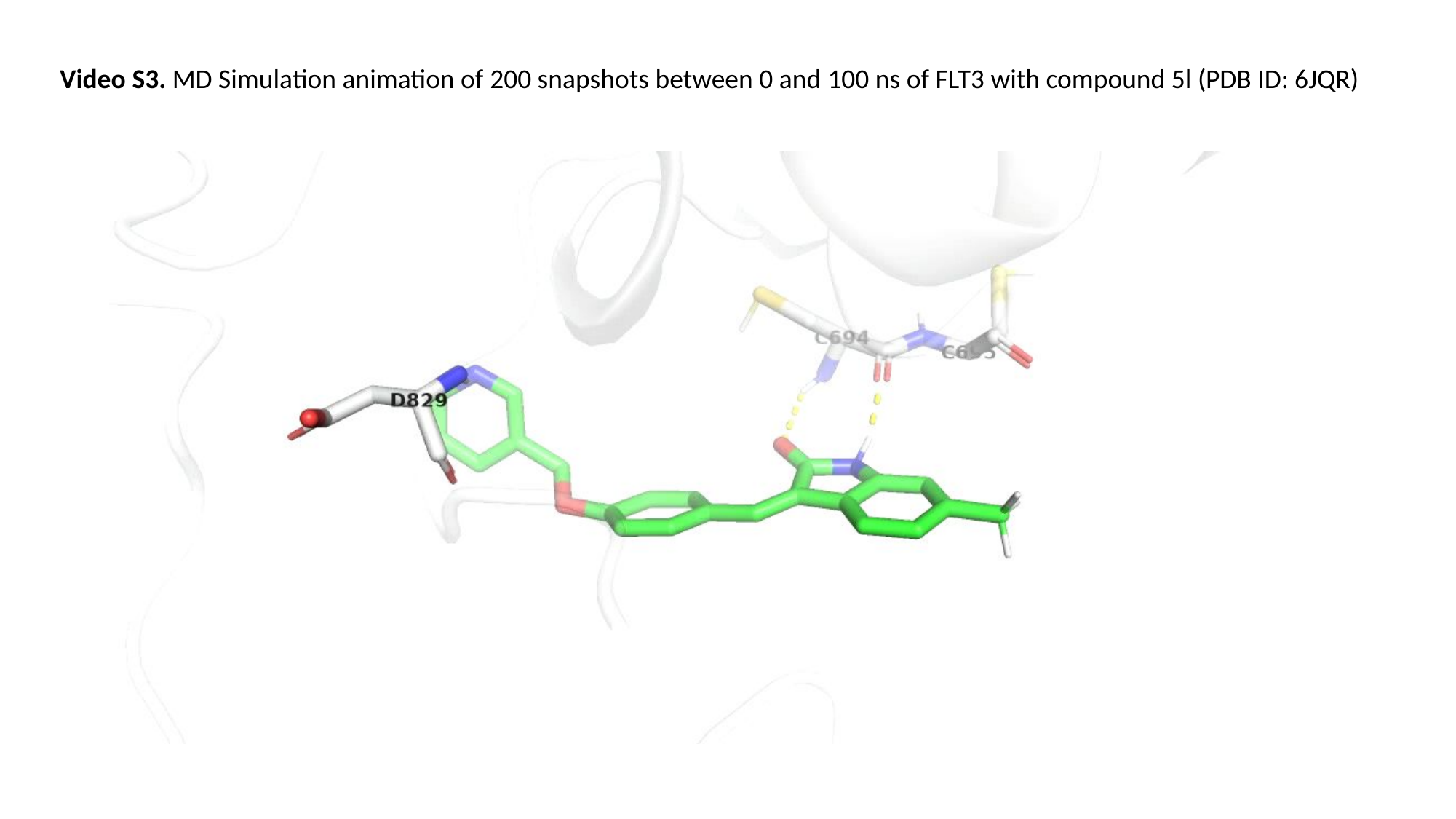

# Video S3. MD Simulation animation of 200 snapshots between 0 and 100 ns of FLT3 with compound 5l (PDB ID: 6JQR)

## Slide 4
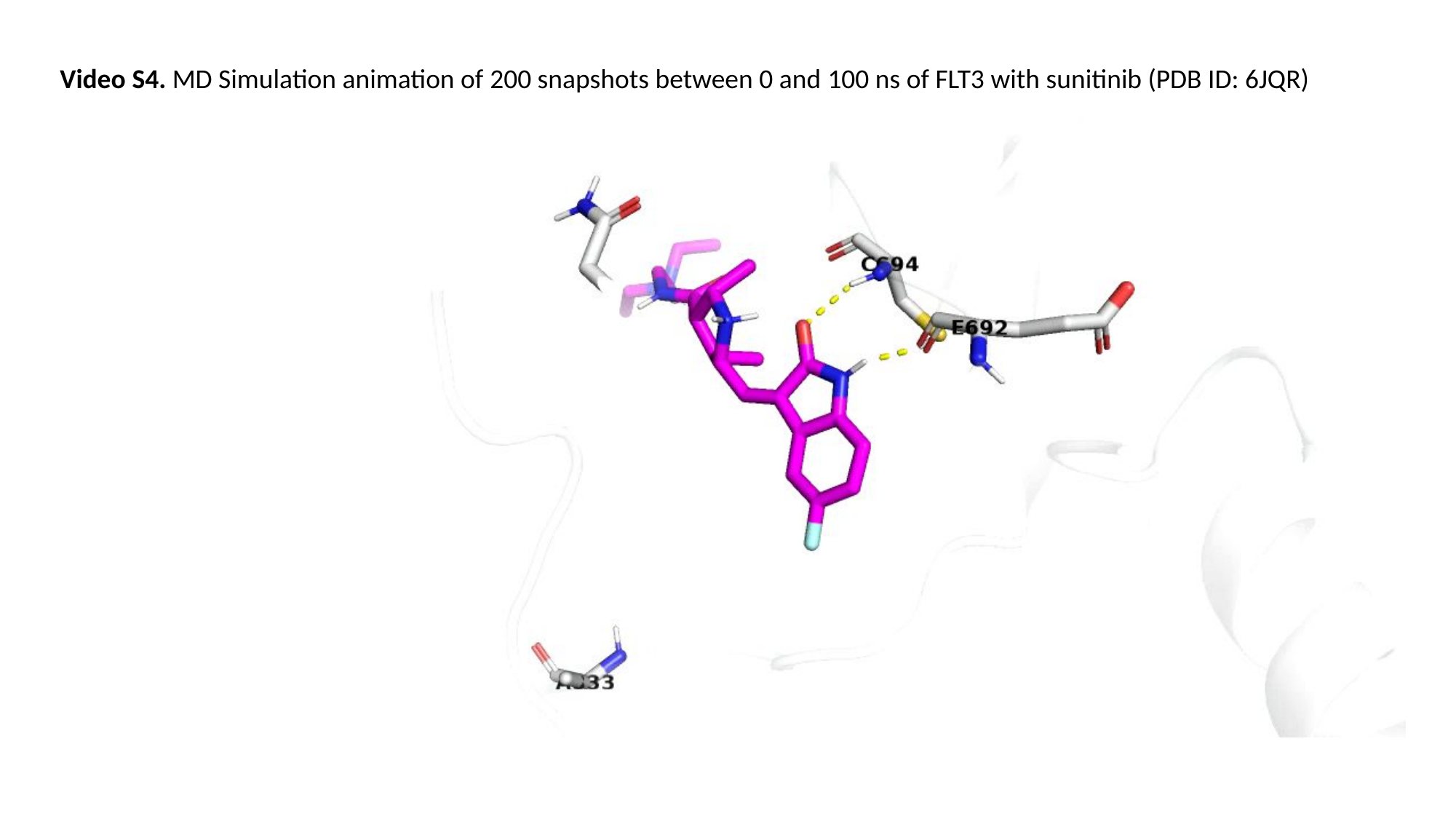

# Video S4. MD Simulation animation of 200 snapshots between 0 and 100 ns of FLT3 with sunitinib (PDB ID: 6JQR)
